# Blood test trend versus single threshold abnormality to discriminate cancer from non-cancer in patients with unexpected weight loss: A retrospective cohort study

**DOI:** 10.1371/journal.pmed.1004956

**Published:** 2026-09-17

**Authors:** Brian D. Nicholson, Clare R. Bankhead, Sufen Zhu, Jason Lee Oke, Cynthia Wright Drakesmith, Rafael Perera, Frederick D. Richard Hobbs, Pradeep S. Virdee

**Affiliations:** 1 Nuffield Department of Primary Care Health Sciences, University of Oxford, Oxford, United Kingdom; 2 Oxford Institute of Digital Health, Oxford, United Kingdom; undefined, UNITED KINGDOM OF GREAT BRITAIN AND NORTHERN IRELAND

## Abstract

**Background:**

Unexpected weight loss (UWL) is a non-specific cancer symptom. Abnormalities in the most recent blood test contribute to cancer referral decisions in primary care. Interpreting historical changes (trends) in blood tests could enhance discrimination for undiagnosed cancer. We aimed to assess the discrimination of blood test trend for undiagnosed cancer and compare this to that of blood test abnormality in patients presenting to primary care with UWL.

**Methods and findings:**

We conducted a retrospective cohort study of patients presenting to English primary care with UWL aged ≥18 years between 01/01/2000 and 31/12/2018 in the Clinical Practice Research Datalink. We extracted all quantitative results for 26 blood tests prior to the date of UWL. Overall and site-specific cancer diagnosis was ascertained using linked National Cancer Registrations Data. Cox models, unadjusted and adjusted for age and sex, estimated cancer risk based on blood test abnormalities (such as low albumin or raised platelets) co-occurring with UWL and joint models assessed trends in each blood test over 1-, 3-, 5-, and 10-year periods prior to UWL. The area under the curve (AUC) was used to assess discrimination, with 95% confidence intervals (CIs). Amongst 275,205 UWL patients, 5.0% (*n* = 13,798) with cancer, the median (interquartile range (IQR)) number of blood tests per person over the 10 years ranged from 2 (IQR [12–3]) for 3 types of tests to 4 (IQR [2–7]) for 12 types of tests. The median (IQR) time between the first and last blood test per person was 5.2 (IQR [1.5–8.2]) years cases and 4.3 (IQR [1.0–7.7]) years for those cancer-free. The median time between the last blood test and UWL per person was 0.1 (IQR [0.0–0.6]) years for cases and 0.3 (IQR [0.0–1.1]) years for those cancer-free. Adjusted blood test abnormalities and trends were more discriminative than the unadjusted equivalent. Adjustment resulted in higher AUCs for trend compared to the equivalent blood test abnormality on 34 occasions, with highest AUC of 0.82 (95% CI [0.81, 0.83]). This included 26 trends for cancer overall, mean cell volume trend for bowel cancer and lymphoma, white blood count and neutrophil trend for lung cancer, and aspartate aminotransferase, red blood cell count, haematocrit, and platelet-to-lymphocyte ratio trend for prostate cancer. A limitation of our modelling strategy is that it did not take into account the bias in who was tested and our trend analysis was also restricted to patients with 2 or more tests in each trend window.

**Conclusions:**

Blood test abnormalities should be interpreted after age and sex adjustment to improve triage for cancer investigation in patients attending primary care with UWL. Evaluating historical trend offers further discrimination after adjustment for some test-cancer combinations.

## Author summary

**Why was this study done?:**

- Abnormal blood test results, such as low haemoglobin or raised platelets, support cancer risk assessment in primary care.
- Monitoring trends (changes over time) in blood test results could enhance cancer risk assessment.
- No study has assessed the potential of blood test trends in comparison to blood test abnormalities in patients who present with non-specific symptoms, such as unexpected weight loss (UWL).

**What did the researchers do and find?:**

- We conducted a cohort study using electronic health records data for 275,205 patients with UWL attending English primary care.
- We compared the discrimination of trends in 26 blood tests prior to UWL for a new cancer diagnosis with the abnormality for the same blood test that is used in clinical practice.
- We found that age- and sex-adjusted blood test abnormalities and trends were more discriminative than unadjusted blood test trends and abnormalities, and for 34 test-cancer combinations adjusted trends were more discriminative than the equivalent adjusted blood test abnormality, including 26 trends for cancer overall, mean cell volume trend for bowel cancer and lymphoma, white blood count and neutrophil trend for lung cancer, and aspartate aminotransferase, red blood cell count, haematocrit, and platelet-to-lymphocyte ratio trend for prostate cancer.

**What do these findings mean?:**

- Blood test results should be adjusted by age and sex to improve patient selection for cancer investigation in patients attending primary care with UWL.
- Blood test trends over repeat tests could further enhance patient selection in some test-cancer combinations.
- Our analysis uses routinely collected data so is limited to when patients had blood tests in clinical practice and to patients who had blood tests.

## Abstract 3

In this retrospective cohort study, Brian D. Nicholson and colleagues evaluate the potential of blood test trends in comparison to blood test abnormalities for cancer risk assessment in patients who present with non-specific symptoms, such as unexpected weight loss.

## Introduction

The current evidence base for using non-specific blood tests to risk stratify for cancer in primary care is almost entirely limited to unadjusted blood test abnormalities of single tests [1–3]. For most blood test abnormalities, the associated risk of a single cancer is too low to warrant urgent cancer investigation. Low albumin, raised platelets, raised calcium, and raised inflammatory markers increase the risk of cancer overall above the 3% threshold recommended by the National Institute for Health and Care Excellence (NICE) for urgent investigation for cancer in England and Wales [1]. Our recent work identified the blood test abnormalities associated with increased risk of cancer overall in patients with unexpected weight loss (UWL) [4,5], who often experience diagnostic challenges [6–8], but blood tests are common [9]. For non-specific symptoms such as UWL, these abnormalities give general practitioners (GPs) an indication that cancer may be present, but uncertainty remains over which cancer(s) should be investigated.

Current primary care clinical guidelines focus on selecting patients for cancer investigation based on combinations of risk factors, signs, symptoms, and blood test abnormalities [1]. Monitoring temporal changes (or trends) in blood tests may enhance cancer risk stratification over and above acting on the only most recent blood test result [10–12]. A patient with a low-normal haemoglobin may not be considered high-risk if the haemoglobin result is interpreted in relation to a binary threshold, but a low-normal result following a period of high-normal results may represent an opportunity for cancer investigation. However, we have found no study that compared the cancer risk associated with blood test trends to the current standard of using the most recent blood test abnormality and no study exploring the role of blood test trend in patients presenting with non-specific symptoms in primary care, such as UWL [13].

Our aim was to explore the discrimination of blood test trends for a new diagnosis of cancer compared to the equivalent blood test abnormality in patients presenting with UWL to primary care, by cancer type and time period of trend.

## Methods

Study reporting follows the STrengthening the Reporting of OBservational studies in Epidemiology (STROBE), REporting of studies Conducted using Observational Routinely-collected health Data (RECORD), and Standards for Reporting of Diagnostic Accuracy Studies (STARD) guidelines. Populated checklists for each are in the Supplementary files.

### Study design

A retrospective cohort study was performed using data from 01/01/2000 to 31/12/2018 in the English primary care Clinical Practice Research Datalink (CPRD) Aurum database. The data were linked at a patient-level to the National Cancer Registration and Analysis Service (NCRAS) Cancer Registration, Office of National Statistics (ONS) Death Registration, Hospital Episode Statistics (HES), and Small Area Index of Multiple Deprivation (IMD) databases. The end date of 2018 coincided with the most recent NCRAS data download for cancer outcomes.

### Participants

We included patients with UWL, with their first coded UWL selected. Patients were aged at least 18 years at UWL, were eligible for linkage to NCRAS, HES, and ONS, and had at least 12 months of registration with their practice and at least one blood test within the 10-year period prior to UWL. Patients with a prescription of weight-reducing medication (orlistat) or bariatric surgery in the six months prior to UWL or a history of cancer prior to UWL were excluded. Systematized Nomenclature of Medicine-Clinical Terms (SNOMED-CT) codes are in https://github.com/PradeepVirdee/CPRD_UWL_TrendVsAbno_Cancer [14].

### Test methods

#### Outcome.

The primary outcome was any primary cancer diagnosed within six months following the UWL. Six months was chosen because previous research demonstrated that the risk of cancer diagnosis after six months following UWL returned to the background levels expected at the patient’s age and sex [15]. Non-melanoma skin cancer, in situ, benign, ill-defined, or uncertain cancers were excluded. Cancers were also categorised into 20 groupings based on cancer site. Cancer cases were determined from the NCRAS database, with additional cases identified as the earliest diagnosis among CPRD, HES, and ONS. Cancer International Classification of Diseases, 10th Revision (ICD10) codes were in the format ‘C###’ and SNOMED-CT codes are in https://github.com/PradeepVirdee/CPRD_UWL_TrendVsAbno_Cancer [14].

Time to diagnosis was defined as the number of days from UWL to cancer diagnosis within 6 months. Patients without a diagnosis in this 6-month period were censored at the earliest of death, de-registration with the practice, data cut date for the practice, 31/12/2018 (study end date), or six months following UWL.

#### Clinical features.

Sociodemographic features closest recorded prior to UWL were extracted for each patient. Pre-existing comorbidities were identified. Co-occurring signs and symptoms, included in NICE guidance [1], closest to UWL in the three months before to one month after the UWL date were identified. We extracted blood tests commonly available in primary care: components of the full blood count, liver function tests, and inflammatory markers (Table A in S1 Appendix). These tests were decided a priori, as our goal was to study commonly available tests, to maximise uptake and impact of abnormality and trend, and tests that are triggered by UWL presentation. Continuous results of these blood tests available over 10 years prior to UWL were obtained, with values less than zero dropped, as these were data entry errors, and standardised to the same unit of measure following the approach used in previous work [16]. Previous research has highlighted the potential of inflammatory marker scores to improve cancer risk stratification in patients with UWL, so we also derived the neutrophil-to-lymphocyte ratio (NLR), platelet-to-lymphocyte ratio (PLR), and modified Glasgow Prognostic ratio (mGPR) [17]. Each continuous blood test result was converted into a categorical result (normal/abnormal) using reference ranges used in clinical practice (Table A in S1 Appendix), as the categorical score is used in clinical practice: neutrophil-to-lymphocyte score (NLS), platelet-to-lymphocyte score (PLS), and modified Glasgow Prognostic Score (mGPS), referring to them as tests hereafter.

### Analysis

#### Data analysis.

Analyses were planned a priori and performed using RStudio (R version 4.5.2). Mean with standard deviation (SD) or median with range/interquartile range (IQR) were used to describe continuous data and counts and proportions were used to describe categorical data. Locally Weighted Scatterplot Smoothing (LOWESS) smoothing was used to describe trends in blood tests over time by age and sex graphically.

Joint modelling of longitudinal and time-to-event data was used to assess trends for cancer diagnosis using the JoineRML package. Joint modelling uses mixed-effects modelling for trends and a linked Cox model to provide hazard ratios (HRs) for outcomes [18,19]. A model was developed for each blood test. Mixed-effects models included time from blood test to UWL (years), age at UWL (years), and sex as fixed effects. An interaction between age and time and between age and sex was included to allow for differences in blood test trends between age groups and sex. Each patient was modelled using a random intercept and time to UWL using a random slope, with an unstructured covariance matrix to account for correlation in repeated measures and restricted maximum likelihood estimation. Time to UWL and age at UWL were modelled using piecewise linear splines, with knots at 6 months and 1, 2, and 3 years before UWL for each test. Linear splines and knot locations were chosen based on visual inspection of LOWESS plots and comparison of Akaike Information Criterion (AIC) and Bayesian Information Criterion (BIC) of models using fractional polynomials, restricted cubic splines, linear splines, and different knot locations. The linked Cox model then included the blood test trend (as latent shared random effects from the mixed-effects component) as a predictor for cancer diagnosis.

Univariable analysis included only the trend in the joint model, using blood test trends measured over 1, 3, 5, and 10 years before UWL to assess how the association and discrimination change based on how much pre-UWL trend was used. Our LOWESS plots suggest that cancer-related trends begin to diverge from those in patients without cancer around 3–5 years before diagnosis, so we opted to investigate 1-, 3-, and 5-year trend windows prior to UWL. We expected that earlier blood tests were unlikely to be helpful and chose to assess this also by including a 10-year trend window. In each longitudinal window, we included patients with at least two blood tests to derive a trend, where at least one was co-occurring with UWL (measured in the 3 months prior). The multivariable analysis subsequently adjusted cancer risk for age at UWL (years) and sex, as these are easily accessible in primary care systems. Age and sex were available for all patients. Other data, such as ethnicity and BMI, were considered only to describe the sample, with patients with missing data included in a “Missing” category.

Cox modelling was used to assess the association between blood test abnormality co-occurring with UWL and subsequent cancer, for comparison with trend. Univariable Cox models included blood test abnormality (high = yes/no or low = yes/no, depending on the test) and multivariable models adjusted for age at UWL (years) and sex. Blood test abnormality on the co-occurring test was defined using reference ranges reported in the Oxford Handbook of Clinical Medicine (Table A in S1 Appendix) [20]. For each blood test in the 1-year trend models and abnormality models, we examined the proportional hazards assumption using plots of Schoenfeld residuals—no model violated the proportional hazards assumption (plots are available from the authors).

Performance of trend and abnormality was assessed using the fixed-time area under the curve (AUC) at 6 months following UWL as a high-level summary metric of discrimination across multiple comparisons. As exploratory analyses, we looked at the AUC of abnormality and trend by cancer site and by the number of repeat tests used to derive the trend.

#### Sample size.

We performed a sample size calculation for an AUC for five blood tests (albumin, AST, haemoglobin, CRP, and plasma viscosity—AST and plasma viscosity cohorts are the smallest cohorts) in the 1-year trend window and 2+ blood test cohort (Table B in S1 Appendix). For each test, the null AUC was 0.5 and target AUC was 0.7. A two-sided 0.048% alpha, accounting for multiple testing (a Bonferroni adjustment to a 5% significance level considering 104 test-trend window combinations), and 80% power was used. The calculation also uses the ratio of the number of patients without cancer to the number of patients with cancer, which we based on our observed data. Based on these inputs, 578–625 patients, depending on the test, were required. We repeated the calculation using 90% and 99% power due to our large sample size, with the latter increasing the sample size to 1,055–1,125.

## Results

### Summary of patient data

**Fig 1:**
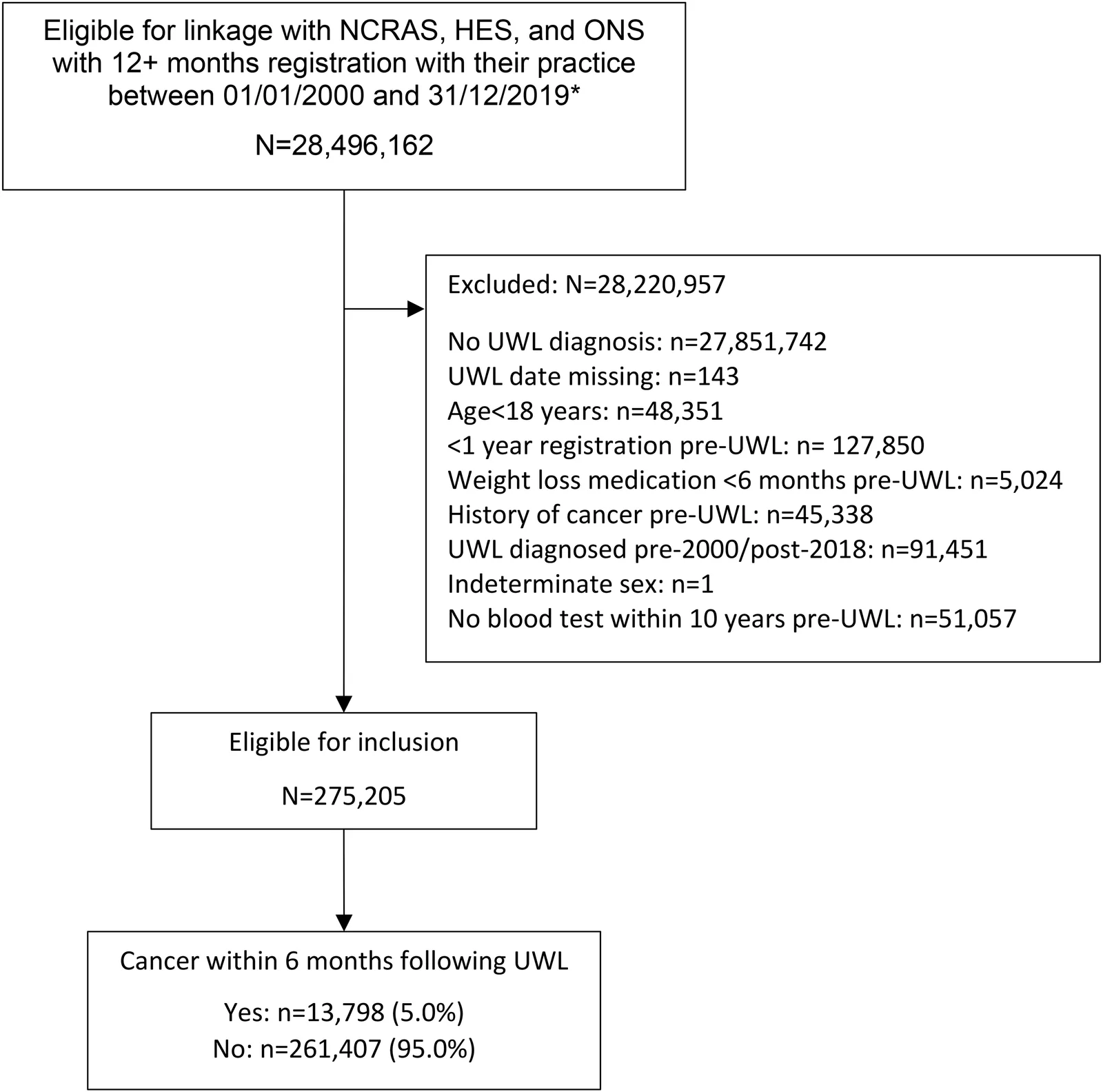
Patient flow diagram. *Primary care patient data was available from January 2000 to December 2019, but was cut off at December 2018, as this was the NCRAS data-cut date for cancer outcomes. Abbreviations: HES, Hospital Episode Statistics; NCRAS, National Cancer Registration and Analysis Service; ONS, Office of National Statistics; UWL, unexpected weight loss.

We included 275,205 eligible patients with UWL (Fig 1). Mean age at UWL was 61.8 (SD [20.1]) and 58.0% (*n* = 159,616) were female (Table 1). The most commonly available blood tests were haemoglobin (95.9%, *n* = 264,012 patients), white blood cell count (WBC) (95.3%, *n* = 262,153), and platelets (95.2%, *n* = 261,985) (Table C in S1 Appendix). The least commonly available were plasma viscosity (9.8%, *n* = 27,022) and mean platelet volume (MPV) (17.5%, *n* = 43,257). Among those included in the trend analysis (those with 2+ tests) (sample sizes are in Table C in S1 Appendix), there was a median of 2 (IQR [1, 3]) blood tests per person over the 10 years for three types of tests and 4 (IQR [2, 7]) for 12 types of tests (results are available on GitHub) [14]. The median number of blood tests per patient generally increased with increasing age, body mass index (BMI), number of co-morbidities, and presence of symptoms. The median number of tests per patient was comparable between men and women and between IMD groups. The most common blood test abnormalities on the most recent blood test were low red blood cell count (RBC) (12.4%, *n* = 34,156 patients), low haemoglobin (8.7%, *n* = 23,919), and high C-reactive protein (CRP) (7.7%, *n* = 21,241) (Table A in S1 Appendix).

**Table 1:** Summary (*n* (%)) of patient demographics and co-occurring clinical features at UWL.

Patient data	Overall	Cancer	No cancer
**Sex**			
Female	159,616 (58.0%)	5,474 (39.7%)	154,142 (59.0%)
Male	115,589 (42.0%)	8,324 (60.3%)	107,265 (41.0%)
**Age (years)**			
18–39	47,027 (17.1%)	116 (0.8%)	46,911 (17.9%)
40–49	30,307 (11.0%)	314 (2.3%)	29,993 (11.5%)
50–59	36,759 (13.4%)	1,122 (8.1%)	35,637 (13.6%)
60–69	42,529 (15.5%)	2,752 (19.9%)	39,777 (15.2%)
70–79	56,650 (20.6%)	4,944 (35.8%)	51,706 (19.8%)
80+	61,933 (22.5%)	4,550 (33.0%)	57,383 (22.0%)
**Ethnicity**			
Asian	13,407 (4.9%)	241 (1.7%)	13,166 (5.0%)
Black	9,004 (3.3%)	291 (2.1%)	8,713 (3.3%)
Mixed	1,831 (0.7%)	39 (0.3%)	1,792 (0.7%)
Other	3,266 (1.2%)	100 (0.7%)	3,166 (1.2%)
White	230,196 (83.6%)	12,084 (87.6%)	218,112 (83.4%)
Missing	17,501 (6.4%)	1,043 (7.6%)	16,458 (6.3%)
**IMD group**			
1 (least deprived)	49,128 (17.9%)	2,579 (18.7%)	46,549 (17.8%)
2	52,739 (19.2%)	2,709 (19.6%)	50,030 (19.1%)
3	52,283 (19.0%)	2,534 (18.4%)	49,749 (19.0%)
4	55,886 (20.3%)	2,779 (20.1%)	53,107 (20.3%)
5 (most deprived)	64,746 (23.5%)	3,170 (23.0%)	61,576 (23.6%)
Missing	423 (0.2%)	27 (0.2%)	396 (0.2%)
**Smoking status**			
Current	89,297 (32.4%)	4,647 (33.7%)	84,650 (32.4%)
Past smoker	67,342 (24.5%)	4,463 (32.3%)	62,879 (24.1%)
Non-smoker	43,651 (15.9%)	1,972 (14.3%)	41,679 (15.9%)
Missing	74,915 (27.2%)	2,716 (19.7%)	72,199 (27.6%)
**Alcohol status**			
Current	131,453 (47.8%)	7,397 (53.6%)	124,056 (47.5%)
Past drinker	293 (0.1%)	16 (0.1%)	277 (0.1%)
Non-drinker	76,037 (27.6%)	3,537 (25.6%)	72,500 (27.7%)
Missing	67,422 (24.5%)	2,848 (20.6%)	64,574 (24.7%)
**BMI**			
Underweight	17,059 (6.2%)	617 (4.5%)	16,442 (6.3%)
Normal	122,654 (44.6%)	5,622 (40.7%)	117,032 (44.8%)
Overweight	65,940 (24.0%)	4,305 (31.2%)	61,635 (23.6%)
Obese	38,218 (13.9%)	1,926 (14.0%)	36,292 (13.9%)
Missing	31,334 (11.4%)	1,328 (9.6%)	30,006 (11.5%)
**No. of comorbidities**			
0	38,364 (13.9%)	1,431 (10.4%)	36,933 (14.1%)
1	53,321 (19.4%)	2,538 (18.4%)	50,783 (19.4%)
2	53,569 (19.5%)	2,838 (20.6%)	50,731 (19.4%)
3	45,012 (16.4%)	2,478 (18.0%)	42,534 (16.3%)
4	33,486 (12.2%)	1,886 (13.7%)	31,600 (12.1%)
5+	51,453 (18.7%)	2,627 (19.0%)	48,826 (18.7%)
**Clinical feature (10 most common)**			
Appetite Loss	7,707 (2.8%)	773 (5.6%)	6,934 (2.7%)
Cough	19,514 (7.1%)	1,215 (8.8%)	18,299 (7.0%)
Dyspepsia	6,100 (2.2%)	581 (4.2%)	5,519 (2.1%)
Fatigue	10,268 (3.7%)	698 (5.1%)	9,570 (3.7%)
Infection Chest	13,614 (4.9%)	918 (6.7%)	12,696 (4.9%)
Pain Abdomen	15,561 (5.7%)	1,530 (11.1%)	14,031 (5.4%)
Pain Back	15,953 (5.8%)	1,034 (7.5%)	14,919 (5.7%)
Pain Chest	7,341 (2.7%)	523 (3.8%)	6,818 (2.6%)
Shortness Of Breath	16,615 (6.0%)	1,185 (8.6%)	15,430 (5.9%)
Urinary Tract Infection	8,615 (3.1%)	506 (3.7%)	8,109 (3.1%)
**Total**	275,205	13,798	261,407

Abbreviations: IMD, index of multiple deprivation; BMI, body mass index.

There were 13,798 (5.0%) patients diagnosed with cancer within 6 months following UWL, with lung (1.13%, *n* = 3,120) and colorectal (0.78%, *n* = 2,150) most commonly diagnosed (Table 2). There were 6.5% (*n* = 17,909) of patients who were censored before reaching the end of 6-month follow-up (4.4%, *n* = 12,221 deaths; 2.1%, *n* = 5,688 de-registration with the practice, data cut date for the practice, 31/12/2018, or 6 months following UWL). The median time between the first and last blood test per person was 5.2 (IQR [1.5, 8.2]) years for patients with subsequent cancer and 4.3 (IQR [1.0, 7.7]) years for patients without (Fig A in S1 Appendix). The median time between the last blood test and UWL per person was 0.1 (IQR [0.0, 0.6]) years (or 1.2 months) for patients with subsequent cancer and 0.3 (IQR [0.0, 1.1]) years (or 3.6 months) for patients without.

**Table 2:** Summary of cancer diagnosis within 6 months following UWL.

Cancer in 6 months	Cancer in 6 months
Yes	13,798 (5.0%)
No	261,407 (95.0%)
Bladder	356 (0.13%)
Bowel	2,170 (0.79%)
Brain	68 (0.02%)
Breast	399 (0.14%)
Head and neck	218 (0.08%)
Kidney	532 (0.19%)
Leukaemia	291 (0.11%)
Liver	369 (0.13%)
Lung	3,120 (1.13%)
Lymphoma	722 (0.26%)
Melanoma of skin	85 (0.03%)
Myeloma	232 (0.08%)
Oesophagus	773 (0.28%)
Other	732 (0.27%)
Ovarian	244 (0.09%)
Pancreas	1,226 (0.45%)
Prostate	1,197 (0.43%)
Stomach	920 (0.33%)
Thyroid	51 (0.02%)
Uterine	93 (0.03%)

Abbreviations: UWL, unexpected weight loss.

**Fig 2:**
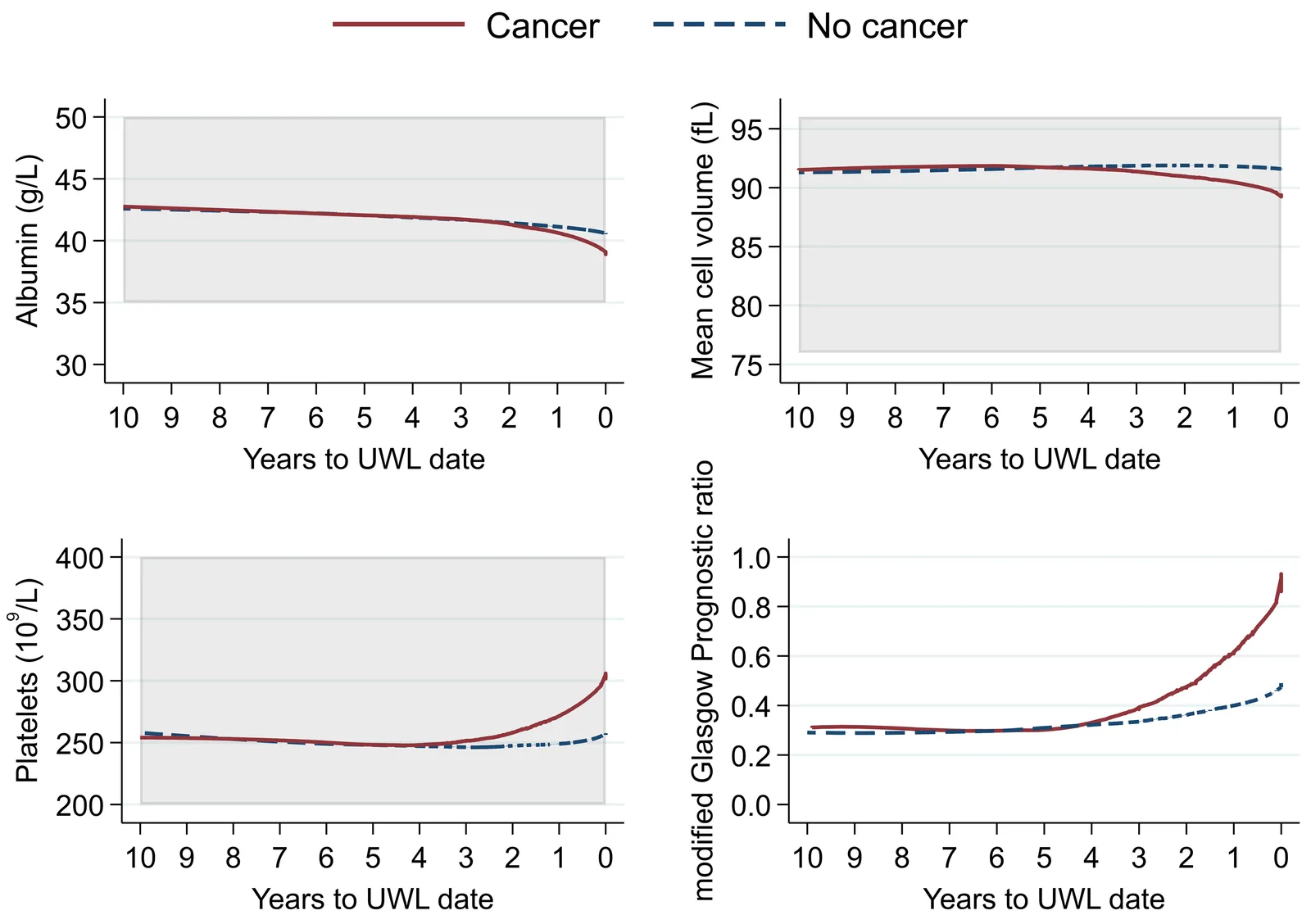
Trends in four blood tests in men aged 70 years at UWL (as examples). LOWESS trends are presented, which represent the observed population-level trend in the study sample. The overlaid grey box is the reference range used in practice for that for that test. Abbreviations: mGPR, modified Glasgow Prognostic ratio; UW, unexpected weight loss.

Graphs of blood test trends over the 10-year study period are in Figs 2 and B–AA in S1 Appendix. Trends in patients with cancer began to diverge from patients without cancer around 3–5 years before the UWL date. Divergent trends were on average confined within the normal reference range, except for haemoglobin and RBC in patients aged 70+ years and inflammatory markers CRP, erythrocyte sedimentation rate (ESR), and plasma viscosity. Haematocrit and red cell distribution width (RDW) trends also exceeded the normal range limit in younger adults aged 50 years or below.

### Association between blood test trend and cancer diagnosis

#### Unadjusted analyses.

Unadjusted blood test abnormalities for 25 (96.2% of 26) blood tests were associated with cancer overall (95% confidence intervals (CIs) did not contain 1), with no association for alanine transaminase (ALT) (Table 3). However, unadjusted blood test abnormalities were poorly discriminative for cancer overall, with AUCs ranging from 0.5 AUC for mean cell haemoglobin concentration (MCHC) to 0.63 AUC for plasma viscosity (Table D in S1 Appendix).

**Table 3:** Hazard ratio (95% CI) of blood test abnormality and trend for increased 6-month cancer risk following UWL.

Blood test^1^	Model^2^	Abnormality	1-year Trend	3-year Trend	5-year Trend	10-year Trend
Albumin (g/L) declining	Unadjusted	2.622 (2.492, 2.758)	1.076 (1.067, 1.086)	1.062 (1.050, 1.073)	1.053 (1.041, 1.064)	1.044 (1.032, 1.056)
Albumin (g/L) declining	Adjusted	1.951 (1.853, 2.055)	1.081 (1.068, 1.094)	1.074 (1.059, 1.088)	1.067 (1.053, 1.082)	1.054 (1.038, 1.070)
Alkaline phosphatase (iu/L) increasing	Unadjusted	2.210 (2.108, 2.317)	1.006 (1.006, 1.007)	1.006 (1.005, 1.007)	1.005 (1.004, 1.006)	1.005 (1.004, 1.006)
Alkaline phosphatase (iu/L) increasing	Adjusted	1.933 (1.844, 2.027)	1.007 (1.006, 1.008)	1.006 (1.005, 1.008)	1.006 (1.005, 1.007)	1.006 (1.004, 1.007)
ALT (iu/L) declining	Unadjusted	1.018 (0.951, 1.089)	1.004 (1.000, 1.008)	1.004 (0.999, 1.008)	1.004 (1.000, 1.009)	1.002 (0.998, 1.007)
ALT (iu/L) declining	Adjusted	1.135 (1.060, 1.215)	1.005 (1.000, 1.010)	1.002 (0.996, 1.008)	1.004 (0.998, 1.010)	1.000 (0.994, 1.007)
AST (iu/L) declining	Unadjusted	1.368 (1.191, 1.571)	1.002 (0.994, 1.009)	1.006 (0.999, 1.012)	1.003 (0.998, 1.009)	1.004 (0.998, 1.009)
AST (iu/L) declining	Adjusted	1.428 (1.242, 1.640)	1.000 (0.992, 1.008)	1.001 (0.994, 1.009)	1.001 (0.994, 1.009)	1.001 (0.993, 1.008)
Bilirubin (umol/L) declining	Unadjusted	1.091 (1.016, 1.172)	1.032 (1.022, 1.041)	1.019 (1.010, 1.029)	1.019 (1.010, 1.029)	1.019 (1.009, 1.030)
Bilirubin (umol/L) declining	Adjusted	1.258 (1.171, 1.352)	1.020 (1.009, 1.032)	1.022 (1.010, 1.034)	1.022 (1.010, 1.035)	1.021 (1.008, 1.034)
RBC (10^12^/L) declining	Unadjusted	1.948 (1.872, 2.026)	1.004 (0.959, 1.053)	1.040 (0.989, 1.092)	1.020 (0.970, 1.073)	1.022 (0.968, 1.078)
RBC (10^12^/L) declining	Adjusted	1.203 (1.153, 1.255)	1.025 (0.962, 1.092)	1.030 (0.970, 1.094)	1.009 (0.950, 1.071)	1.006 (0.945, 1.071)
Haemoglobin (g/L) declining	Unadjusted	2.768 (2.660, 2.880)	1.017 (1.015, 1.020)	1.017 (1.014, 1.020)	1.012 (1.009, 1.015)	1.013 (1.010, 1.016)
Haemoglobin (g/L) declining	Adjusted	1.786 (1.712, 1.863)	1.017 (1.013, 1.021)	1.015 (1.012, 1.019)	1.014 (1.011, 1.018)	1.014 (1.010, 1.017)
Haematocrit (L/L) declining	Unadjusted	2.093 (2.004, 2.186)	1.001 (0.993, 1.008)	1.002 (0.993, 1.011)	1.000 (0.991, 1.008)	1.006 (0.997, 1.015)
Haematocrit (L/L) declining	Adjusted	1.738 (1.663, 1.817)	1.001 (0.991, 1.011)	1.006 (0.994, 1.018)	1.007 (0.997, 1.017)	1.003 (0.992, 1.014)
MCH (pg) declining	Unadjusted	2.359 (2.234, 2.491)	1.135 (1.119, 1.151)	1.120 (1.101, 1.139)	1.121 (1.100, 1.142)	1.098 (1.078, 1.119)
MCH (pg) declining	Adjusted	2.510 (2.377, 2.651)	1.140 (1.120, 1.161)	1.160 (1.136, 1.183)	1.127 (1.104, 1.152)	1.119 (1.094, 1.143)
MCHC (g/L) declining	Unadjusted	1.396 (1.285, 1.517)	1.001 (1.000, 1.001)	1.001 (1.000, 1.001)	1.001 (1.000, 1.002)	1.000 (0.999, 1.000)
MCHC (g/L) declining	Adjusted	1.437 (1.322, 1.561)	1.001 (1.000, 1.002)	1.000 (0.999, 1.001)	1.001 (1.000, 1.002)	1.001 (1.000, 1.001)
MCV (fL) declining	Unadjusted	2.973 (2.676, 3.304)	1.053 (1.047, 1.058)	1.049 (1.043, 1.056)	1.031 (1.024, 1.038)	1.026 (1.018, 1.033)
MCV (fL) declining	Adjusted	3.463 (3.117, 3.848)	1.055 (1.047, 1.063)	1.047 (1.038, 1.055)	1.053 (1.044, 1.062)	1.048 (1.038, 1.057)
RDW (%) increasing	Unadjusted	1.847 (1.743, 1.957)	1.071 (1.046, 1.095)	1.078 (1.056, 1.100)	1.063 (1.038, 1.089)	1.056 (1.031, 1.081)
RDW (%) increasing	Adjusted	1.485 (1.400, 1.575)	1.062 (1.037, 1.087)	1.076 (1.048, 1.105)	1.064 (1.034, 1.095)	1.066 (1.037, 1.095)
Platelets (10^9^/L) increasing	Unadjusted	2.910 (2.760, 3.068)	1.005 (1.004, 1.005)	1.004 (1.003, 1.004)	1.004 (1.003, 1.004)	1.004 (1.003, 1.005)
Platelets (10^9^/L) increasing	Adjusted	2.973 (2.819, 3.135)	1.005 (1.004, 1.005)	1.004 (1.003, 1.005)	1.004 (1.003, 1.004)	1.003 (1.003, 1.004)
MPV (fL) declining	Unadjusted	1.420 (1.141, 1.767)	1.136 (1.062, 1.215)	1.078 (1.024, 1.135)	1.080 (1.025, 1.139)	1.056 (1.001, 1.114)
MPV (fL) declining	Adjusted	1.287 (1.034, 1.602)	1.149 (1.070, 1.235)	1.105 (1.040, 1.174)	1.093 (1.029, 1.161)	1.094 (1.030, 1.161)
WBC (10^9^/L) increasing	Unadjusted	2.486 (2.357, 2.622)	1.197 (1.177, 1.217)	1.176 (1.152, 1.200)	1.152 (1.126, 1.178)	1.179 (1.154, 1.203)
WBC (10^9^/L) increasing	Adjusted	2.471 (2.342, 2.606)	1.221 (1.194, 1.248)	1.194 (1.164, 1.224)	1.178 (1.148, 1.208)	1.161 (1.129, 1.193)
Basophil count (10^9^/L) increasing	Unadjusted	1.093 (1.049, 1.140)	3.384 (0.929, 12.321)	3.025 (0.790, 11.583)	9.487 (2.425, 37.110)	3.860 (1.055, 14.124)
Basophil count (10^9^/L) increasing	Adjusted	1.096 (1.052, 1.143)	1.936 (0.389, 9.628)	1.017 (0.197, 5.263)	3.028 (0.630, 14.560)	1.824 (0.371, 8.970)
Eosinophil count (10^9^/L) increasing	Unadjusted	1.125 (1.040, 1.217)	1.175 (0.762, 1.813)	1.012 (0.713, 1.437)	1.030 (0.715, 1.481)	1.103 (0.752, 1.616)
Eosinophil count (10^9^/L) increasing	Adjusted	1.013 (0.936, 1.096)	1.513 (0.984, 2.326)	1.460 (0.935, 2.283)	1.613 (1.017, 2.558)	1.321 (0.835, 2.092)
Lymphocyte count (10^9^/L) increasing	Unadjusted	1.694 (1.586, 1.811)	1.043 (0.980, 1.111)	1.118 (1.045, 1.196)	1.121 (1.043, 1.204)	1.182 (1.106, 1.262)
Lymphocyte count (10^9^/L) increasing	Adjusted	1.191 (1.113, 1.273)	1.043 (0.954, 1.139)	1.096 (1.003, 1.198)	1.110 (1.022, 1.206)	1.061 (0.972, 1.158)
Monocyte count (10^9^/L) increasing	Unadjusted	2.474 (2.373, 2.580)	4.869 (4.024, 5.892)	4.097 (3.294, 5.096)	4.878 (3.891, 6.116)	3.812 (3.028, 4.798)
Monocyte count (10^9^/L) increasing	Adjusted	1.988 (1.907, 2.074)	6.628 (5.151, 8.528)	5.345 (4.073, 7.015)	5.120 (3.869, 6.777)	4.341 (3.277, 5.752)
Neutrophil count (10^9^/L) increasing	Unadjusted	2.659 (2.528, 2.796)	1.255 (1.230, 1.280)	1.233 (1.203, 1.263)	1.205 (1.173, 1.239)	1.198 (1.164, 1.233)
Neutrophil count (10^9^/L) increasing	Adjusted	2.506 (2.383, 2.636)	1.251 (1.218, 1.286)	1.226 (1.188, 1.265)	1.217 (1.178, 1.258)	1.230 (1.189, 1.272)
CRP (mg/L) increasing	Unadjusted	3.020 (2.820, 3.234)	1.021 (1.019, 1.024)	1.023 (1.021, 1.025)	1.023 (1.021, 1.026)	1.024 (1.020, 1.027)
CRP (mg/L) increasing	Adjusted	2.472 (2.304, 2.653)	1.021 (1.018, 1.023)	1.023 (1.020, 1.026)	1.024 (1.021, 1.026)	1.024 (1.021, 1.026)
ESR (mm/h) increasing	Unadjusted	3.079 (2.900, 3.270)	1.021 (1.019, 1.024)	1.023 (1.021, 1.025)	1.023 (1.021, 1.026)	1.023 (1.020, 1.026)
ESR (mm/h) increasing	Adjusted	2.620 (2.466, 2.783)	1.021 (1.018, 1.023)	1.023 (1.020, 1.026)	1.024 (1.021, 1.026)	1.024 (1.021, 1.027)
Plasma viscosity (mPa.S) increasing	Unadjusted	3.689 (3.173, 4.291)	10.649 (6.252, 18.136)	2.092 (1.393, 3.143)	2.085 (1.671, 2.601)	2.083 (1.635, 2.653)
Plasma viscosity (mPa.S) increasing	Adjusted	3.112 (2.673, 3.623)	9.992 (5.545, 18.005)	9.754 (5.513, 17.256)	15.429 (8.890, 26.775)	25.259 (14.294, 44.635)
NLR increasing	Unadjusted	1.707 (1.596, 1.826)	1.179 (1.153, 1.206)	1.141 (1.108, 1.175)	1.144 (1.107, 1.182)	1.100 (1.064, 1.137)
NLR increasing	Adjusted	1.196 (1.118, 1.281)	1.226 (1.185, 1.268)	1.152 (1.112, 1.193)	1.122 (1.082, 1.164)	1.134 (1.092, 1.179)
PLR increasing	Unadjusted	1.725 (1.611, 1.846)	1.003 (1.002, 1.004)	1.002 (1.002, 1.003)	1.002 (1.001, 1.003)	1.001 (1.000, 1.002)
PLR increasing	Adjusted	1.202 (1.123, 1.288)	1.003 (1.002, 1.004)	1.003 (1.002, 1.003)	1.002 (1.001, 1.003)	1.002 (1.001, 1.003)
mGPR increasing	Unadjusted	2.771 (2.539, 3.024)	1.923 (1.712, 2.159)	2.153 (1.916, 2.418)	2.131 (1.944, 2.336)	2.165 (1.962, 2.388)
mGPR increasing	Adjusted	1.998 (1.827, 2.184)	1.991 (1.713, 2.314)	2.124 (1.891, 2.385)	2.202 (1.967, 2.465)	2.240 (2.000, 2.509)

1 HRs are presented for a one-unit change in the blood test per year and the associated hazard rate of cancer within 6 months.

2 Adjusted HRs for trend have been adjusted for age at UWL and sex.

Abbreviations: UWL, unexpected weight loss; ALT, alanine aminotransferase; AST, aspartate aminotransferase; RBC, red blood cell count; MCH, mean cell haemoglobin; MCHC, mean cell haemoglobin concentration; MCV, mean cell volume; RDW, red cell distribution width; MPV, mean platelet volume; WBC, white blood cell count; CRP, C-reactive protein; ESR, erythrocyte sedimentation rate; NLR, neutrophil-lymphocyte ratio; PLR, platelet-lymphocyte ratio; mGPR, modified Glasgow Prognostic ratio.

Unadjusted 5-year trends for 21 (80.8% of 26) blood tests were associated with cancer overall (Table 3). The trend was declining for seven (26.9% of 26) tests and increasing for 14 (53.8% of 26). Unadjusted associations were maintained when extending the trend to 10 years, except for mean cell haemoglobin (MCH), and after shortening the trend to 3 and 1 years, except for lymphocyte count over 1-year, and MCHC over 1 and 3 years. HRs for blood test trend were rescaled to represent unit changes per year that are more clinically meaningful (Table E in S1 Appendix).

**Fig 3:**
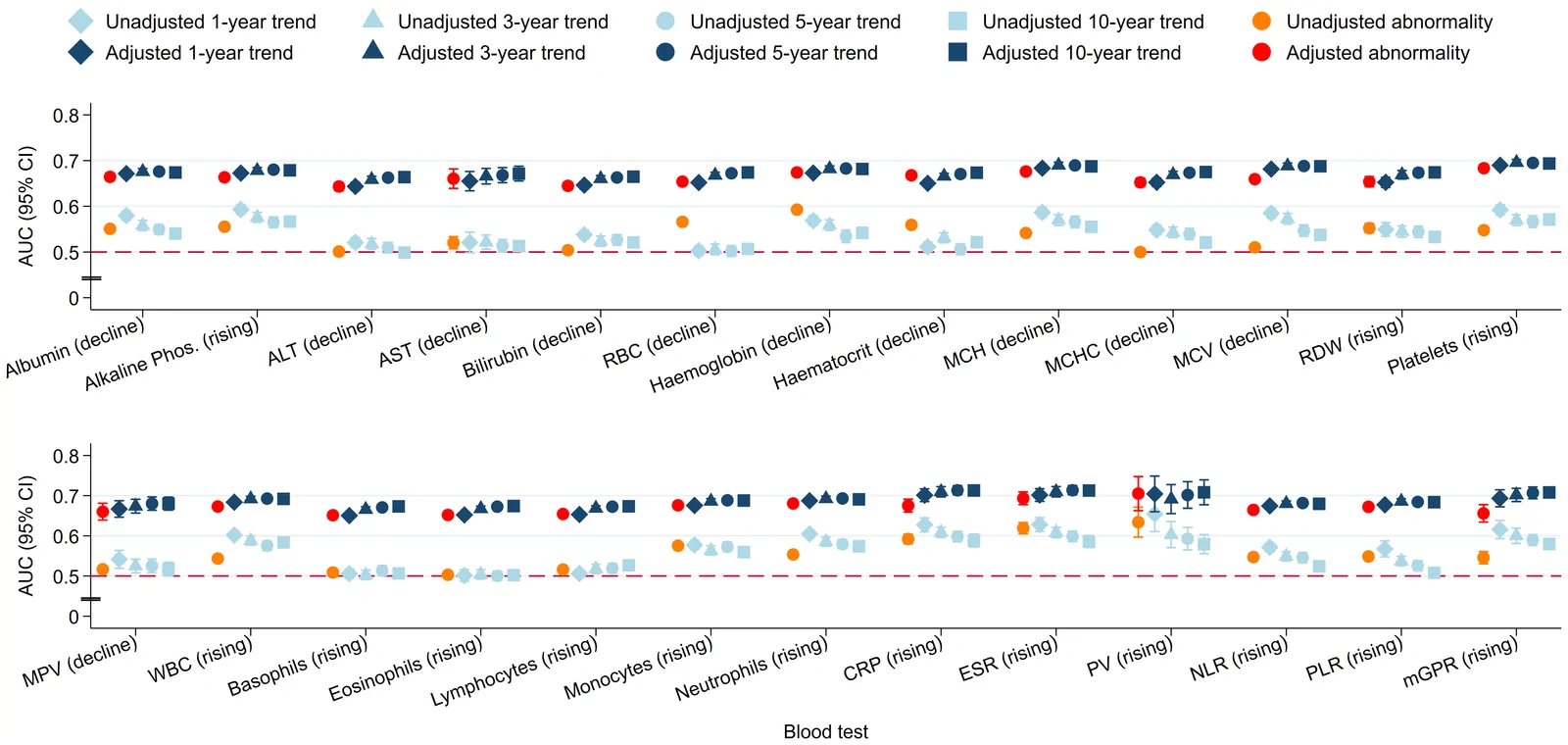
AUC (95% CI) of blood test trend and abnormality for 6-month cancer risk following UWL. This figure shows the AUC of unadjusted and age- and sex-adjusted blood test abnormality and blood test trend. The AUC often favoured unadjusted trend than the unadjusted abnormality, particularly for trend over 1 year prior to UWL, with decreasing AUC as increasing earlier trend prior to UWL was used. The AUC was often comparable between adjusted trend than the adjusted abnormality. Abbreviations: UWL, unexpected weight loss AUC, area under the curve; ALT, alanine aminotransferase; AST, aspartate aminotransferase; RBC, red blood cell count; MCH, mean cell haemoglobin; MCHC, mean cell haemoglobin concentration; MCV, mean cell volume; RDW, red cell distribution width; MPV, mean platelet volume; WBC, white blood cell count; CRP, C-reactive protein; ESR, erythrocyte sedimentation rate; PV, plasma viscosity; NLR, neutrophil-lymphocyte ratio; PLR, platelet-lymphocyte ratio; mGPR, modified Glasgow Prognostic ratio.

Unadjusted blood test trends had AUCs for cancer overall ranging from 0.50 (95% CI [0.49, 0.51]) for 10-year ALT to 0.66 (95% CI [0.61, 0.70]) for 1-year plasma viscosity (Table D in S1 Appendix). In general, increasing the trend period resulted in a reduction in the AUC. Unadjusted trends over 1, 3, 5, and 10 years had a higher AUC than the equivalent unadjusted abnormality for 12 (46.2% of 26), 7 (26.9%), 6 (23.1%), and 5 (19.2%) blood tests, respectively (Fig 3).

The largest differences in AUC between unadjusted abnormality and unadjusted trend were observed for mGPR (abnormality: 0.55 (95% CI [0.53, 0.56]); 1-year trend: 0.62 (95% CI [0.59, 0.64])); RBC (abnormality: 0.50 (95% CI [0.49, 0.51]); 1-year trend 0.57 (95% CI [0.56, 0.57])), and mean cell volume (MCV) (abnormality: 0.51 (95% CI [0.51, 0.51]); 1-year trend: 0.58 (95% CI [0.57, 0.60])).

#### Adjusted analyses.

Adjustment of all blood test abnormalities and trends by age and sex resulted in higher AUCs compared to the equivalent unadjusted estimate (Table D in S1 Appendix). Adjusted blood test abnormalities for 25 (96.2% of 26) blood tests were associated with cancer overall, with no association for eosinophil count (Table 3). Adjusted blood test abnormalities had AUCs for cancer overall ranging from 0.64 (95% CI [0.63, 0.65]) for ALT to 0.71 (95% CI [0.66, 0.75]) for plasma viscosity (Table D in S1 Appendix).

There were 21 (80.8% of 26) adjusted blood test trends over 5 years associated with cancer diagnosis overall, with no association for ALT, aspartate aminotransferase (AST), RBC, haematocrit, or MCHC (Table 3). Associations were often maintained when reducing the trend to 1 and 3 years and extending the trend to 10 years. HRs for blood test trend were rescaled to represent unit changes per year that are more clinically meaningful (Table E in S1 Appendix). Adjusted blood test trends had AUCs for cancer overall ranging from 0.64 (95% CI [0.63, 0.65]) for ALT over 1 year 0.71 (95% CI [0.70, 0.72]) for CRP over 3–10 years (Fig 3).

**Fig 4:**
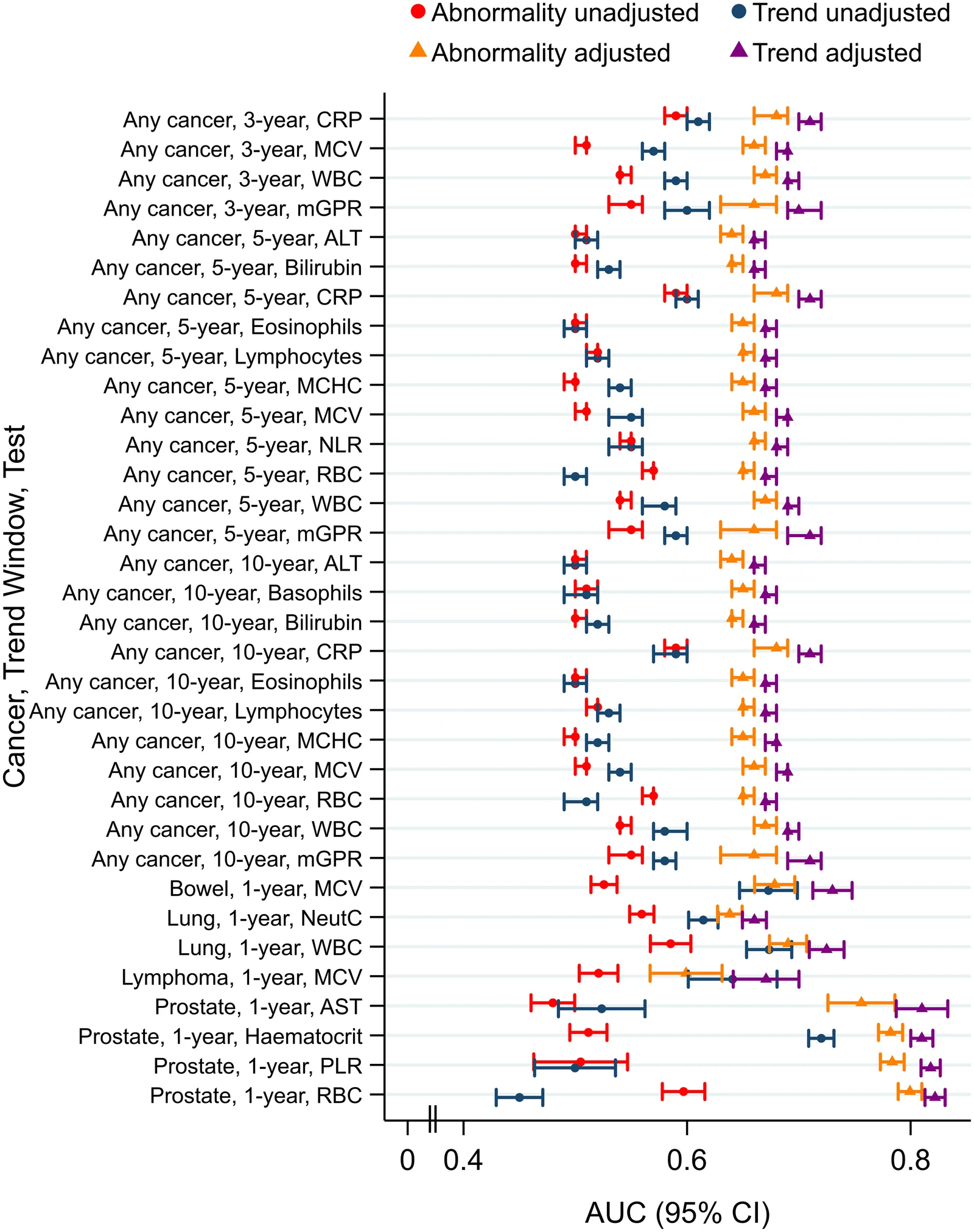
Instances where the age- and sex-adjusted AUC favoured blood test trend over blood test abnormality. This figure portrays the instances where the AUC favoured the age- and sex-adjusted trend than the adjusted abnormality counterpart for cancer overall but also by cancer site. Only instances for cancer overall and by cancer site in the UWL cohort are shown; instances in subgroups, such as by age or sex, are not shown. Abbreviations: AUC, area under the curve; ALT, alanine aminotransferase; AST, aspartate aminotransferase; RBC, red blood cell count; MCHC, mean cell haemoglobin concentration; MCV, mean cell volume; WBC, white blood cell count; CRP, C-reactive protein; Neutc, neNLR, neutrophil-lymphocyte ratio; PLR, platelet-lymphocyte ratio; mGPR, modified Glasgow Prognostic ratio.

There were higher AUCs (95% CI) for trend over 1, 3, 5, and 10 years for cancer overall compared to the equivalent adjusted abnormality for 0, 4 (15.4% of 26), 11 (42.3%), and 11 (42.3%) tests, respectively (Fig 4). There was no difference in AUC between the remaining 26 (100.0% of 26), 22 (84.6%), 15 (57.7%), and 15 (57.7%) abnormality and trend combinations, respectively.

However, differences in the in AUC between adjusted trend and adjusted abnormality were small, with the largest differences observed for mGPR (abnormality: 0.66 (95% CI [0.63, 0.68]); 3-year trend: 0.70 (95% CI [0.69, 0.72])); CRP (abnormality: 0.68 (95% CI [0.66, 0.69]); 3-year trend 0.71 (95% CI [0.70, 0.72])), and MCHC (abnormality: 0.65 (95% CI [0.64, 0.66]); 10-year trend: 0.68 (95% CI [0.67, 0.68])) (Table D in S1 Appendix).

### Subgroup analyses

#### Cancer site.

After adjustment, declining MCV trend was favoured for bowel cancer (0.72 (95% CI [0.70, 0.74])) and lymphoma (0.66 (95% CI [0.63, 0.69])) and rising WBC (0.72 (95% CI [0.70, 0.73])) and neutrophil trend (0.66 (95% CI [0.65, 0.67])) for lung cancer, and AST, RBC, haematocrit, and PLR trend for prostate cancer (Table F in S1 Appendix).

#### Number of tests.

For each blood test trend, the AUC decreased slightly as the number of blood tests used to derive the 1-year trend increased until 4 tests and remained comparable thereafter (Table G in S1 Appendix). However, confidence intervals grew wider as the number of repeat tests increased. Similar patterns were seen between unadjusted and adjusted analyses.

## Discussion

We investigated whether trends in 26 commonly used blood tests might offer improved risk stratification for cancer compared to the most recent blood test abnormality in patients attending primary care with UWL. We explored unadjusted and adjusted associations between blood test abnormalities, and blood test trends over 1, 3, 5, and 10 years, and the diagnosis of cancer overall and by cancer site. Unadjusted analyses demonstrated that blood test abnormalities are weakly associated with cancer and poorly discriminative. Unadjusted blood test trends over 1 and 2 years demonstrated stronger associations with cancer and improved discrimination for cancer compared to unadjusted abnormality. Adjustment for age and sex strengthened associations and improved discrimination. Adjustment resulted in superior discrimination for blood test trend than blood test abnormality for 34 test-cancer combinations. This includes cancer overall across 12 tests over 3 time intervals, 1-year trend in MCV for bowel cancer and lymphoma, 1-year trend in WBC and neutrophils for lung cancer, and 1-year trend in AST, RBC, haematocrit, and PLR for prostate cancer.

This is a large-scale analysis comparing the discrimination of blood test trends for cancer over varying intervals with commonly used blood test abnormalities in a primary care population. We included over 275,000 patients with UWL, and close to 14,000 cancer cases, ensuring reliability and precision of analyses for cancer overall, although confidence intervals remained relatively wide for some tests. This was further demonstrated when we assessed the influence of multiple testing on our findings: we adjusted the 95% CIs to 99.952% CIs, based on a Bonferroni adjustment of a 5% significance level to 0.048% due to 104 test and trend window combinations, but found that the CIs only changed from the 3rd or 4th decimal point onwards. We therefore retained 95% CIs. Precision was retained for lung, bowel, and prostate cancer subgroup analyses as the number of events ranging from 1,197 for prostate to 3,120 for lung, demonstrating that some adjusted trends had greater discrimination than blood test abnormality. Weaker associations were found in subgroups with smaller sample sizes. Future work investigating the clinical utility of trend in more common symptoms than UWL may lead to more precise associations with trend, overall and by subgroup.

A further strength is that we utilised dynamic modelling to assess the association between blood test trend and cancer diagnosis, which can accommodate the unstructured nature of primary care records. Joint modelling for repeated measures data includes a mixed-effects model component to derive the trend and subject-specific random effects to account for sporadic and irregular testing and the correlation between repeat measurements [18,19]. It does not require a specific testing pattern to derive a trend as it accounts for the variation in the timing and frequency of testing and so is appropriate for unstructured routinely collected data. Prior to the last decade, such investigations were not possible, as many statistical software packages did not include these capabilities. Our modelling strategy did not take into account the bias in who was tested, with patients with chronic disease and greater symptom burden more likely to attend primary care and therefore undergo blood testing. Our trend analysis was also restricted to patients with 2 or more tests in each trend window. This could introduce selection bias, as patients with multiple blood tests must be alive for both tests and may have utilised healthcare services compared to those with one blood test. Patients with 2 or more tests could potentially be more likely to be diagnosed with cancer in primary care. However, when conducting our data checks, analyses showed that the cancer incidence in patients with only one test was comparable to patients with two or more tests (results are available from the authors). In future work, we intend to incorporate blood test trends into a prediction models and this would include all patients with UWL who have a test, not just those with 2 or more tests.

We found that trends over 1 year prior to UWL were most discriminative for individual cancers. The median number of test results in a 1-year trend was two, arguably representing a change rather than a trend across three or more measurements. As the latter test result was the same result used to define test abnormality, any additional discrimination is likely based on the nature of the data used in the trend analysis, the timing of the first test, how ‘normal’ and when the first measurement was, and how ‘abnormal’ the second measurement was. This combination of data structure and study design may explain why we did not see a marked additional utility of adjusted trends in the majority of comparisons with adjusted abnormality. We assessed whether cases with more than two tests within a year led to greater utility and found that discrimination decreased between two and four tests and often when incorporating data earlier than 1-year, signalling that the final measurement in the trend is the most discriminative, though confidence intervals grew wider. When UWL is associated with cancer cachexia, blood test abnormalities are expected [21], reflecting underlying metabolic and inflammatory processes, and explaining why blood test abnormalities at the time the patient presents with UWL offer greatest utility to risk stratify patients for cancer investigation. Blood test trends may hold greater utility in patients presenting with symptoms without accompanying underlying metabolic derangement, or in analyses where the symptomatic presentation is not taken into account.

A further limitation is that we relied on patient data from electronic health records to define UWL. However, we build on previous analyses using a validated list of UWL Read codes [22], where UWL code usage was cross-referenced with change in quantitative weight values over time [15], and validated cancer diagnoses recorded primarily in NCRAS, increasing the reliability of the cohort used and cancers diagnosed [14]. As GPs preferentially code clinical features they assess to be associated with cancer, our reported associations may be stronger than if unstructured free-text data were included [23].

A recent systematic review included 23 studies that assessed the association between blood test trend and cancer diagnosis [10]. However, no study compared performance between blood test abnormalities and the equivalent blood test trends to understand in which cases incorporating blood test trend might enhance cancer risk stratification. Existing studies have estimated the diagnostic performance of commonly used blood test abnormalities to risk stratify for cancer in primary care [3–5]. Commonly, the risk of cancer is estimated for a lower bounded age-range [24]. Risk estimates for multiple age-bands, such as decades of age, more closely align to the continuous age-adjustment of blood test abnormalities used in our analysis, with some studies reporting cancer risks within age strata or by sex separately as an actionable proxy for adjustment [25]. Model-based approaches including more specific and static cancer markers such as Cancer Antigen 125 and the Faecal Immunochemical Test (FIT) are being introduced and incorporated into clinical guidance [26–28]. Our analysis adds to these studies by considering age as a continuous variable, blood test trend, by focussing on the UWL cohort, and by considering a broader range of tests commonly used in clinical practice.

We identified scenarios in which blood test trends may warrant further evaluation to focus or augment the diagnostic work-up in patients presenting with UWL. Changes over a short 1-year period were most discriminative for specific cancers. Whilst these blood tests are commonly used in practice, they are not commonly used to test for the cancers we report an association for trend. Many of these cancers often have an existing, more specific, primary care test. Further research should therefore investigate associations between MCV trend and colorectal cancer in combination or as triage to FIT testing, AST, RBC, haematocrit, and PLR trend in relation to Prostate Specific Antigen (PSA) testing for prostate cancer, and WBC trend as triage to thoracic imaging for lung cancer. As adjusted trend outperformed abnormality most often for cancer diagnosis overall, as opposed to individual cancer sites, this offers a potential approach to enhance patient selection for multi-cancer testing when these technologies are ready for clinical evaluation in primary care [29].

Our findings illustrate that blood test abnormalities and trends have greater discrimination for cancer diagnosis overall and for specific cancers after adjustment for age and sex. In clinical practice, most blood test results are interpreted around a fixed threshold without adjustment [1]. Adjustment could be performed at the laboratory level and an adjusted risk estimate could be reported to primary care with the unadjusted test result, or unadjusted results received from the laboratory could be adjusted within the primary care electronic health record to produce the associated risk estimate. Our findings highlight test-cancer pairings for which trend has the potential to enhance the selection of patients with UWL for cancer investigation in primary care. Dynamic prediction models that utilise age, sex, and the combinations of blood test abnormalities and blood test trends identified in this manuscript could be combined with additional covariates, such as ethnicity, comorbidity, deprivation score, and smoking status. We did not adjust for these additional variables, as we prioritised complete and accurate data electronic health records data (age, sex, and blood tests) and our analysis is exploratory. Such an approach could be implemented into primary care electronic systems after validation to support GP decision-making. These changes would require modifications to current electronic health record functionality and implementation evaluations should consider their impact on clinical workflows, risk communication, clinician behaviour, cost-effectiveness, and clinical outcomes [30].

Adjusted trends over three or more years in 12 blood test components showed superior discrimination to adjusted abnormality. However, the improvements were modest and there are significant implementation considerations to analysing blood test trends within the primary care electronic health record that need to be studied to permit real-time trend analysis. Our results will also need external validation, by deriving performance measures in further primary care datasets. It may be that the more immediate and substantial gains made by focussing on age and sex adjustment of blood test abnormalities should be prioritised over the smaller additional gains made by adding trend analysis. This may not be the case for symptomatic presentations with less metabolic derangement, or for analysing blood test trend without accounting for symptoms, but this needs further investigation, but this needs further investigation.

Blood test abnormalities should be interpreted after age and sex adjustment to optimise patient selection for cancer investigation in patients attending primary care with UWL. Evaluating historical trend within 1 year prior to UWL offers further discrimination, after adjustment by age and sex, for some test-cancer combinations, but the utility of incorporating trend to improve patient triage requires further investigation. Simple decision rules or a risk prediction model incorporating this data could support patient selection in primary care.

### Ethics approval

Ethical approval was provided by the Research Data Governance and Independent Scientific Advisory Committee through the CPRD on behalf of the Health Research Authority (protocol number: 22_001798).

## Supporting information

S1 Appendix Supplementary figures and tables. (DOCX)

S1 Checklist STROBE+RECORD checklist (https://doi.org/10.1016/j.jclinepi.2007.11.008). Checklist is protected under Creative Commons Attribution (CC BY) license.
(DOCX)

S2 Checklist STARD checklist. (DOCX)

## Abbreviations

AIC: Akaike Information Criterion
ALT: alanine transaminase
AUC: area under the curve
BIC: Bayesian Information Criterion
BMI: body mass index
CIs: confidence intervals
CPRD: Clinical Practice Research Datalink
CRP: C-reactive protein
ESR: erythrocyte sedimentation rate
FIT: Faecal Immunochemical Test
GPs: general practitioners
HES: Hospital Episode Statistics
HRs: hazard ratios
ICD10: International Classification of Diseases, 10th Revision
IMD: Index of Multiple Deprivation
IQR: interquartile range
LOWESS: Locally Weighted Scatterplot Smoothing
MCV: mean cell volume
MCH: mean cell haemoglobin
MCHC: mean cell haemoglobin concentration
mGPR: modified Glasgow Prognostic ratio
mGPS: modified Glasgow Prognostic Score
MPV: mean platelet volume
NCRAS: National Cancer Registration and Analysis Service
NICE: National Institute for Health and Care Excellence
NLR: neutrophil-to-lymphocyte ratio
NLS: neutrophil-to-lymphocyte score
ONS: Office of National Statistics
PLR: platelet-to-lymphocyte ratio
PLS: platelet-to-lymphocyte score
PSA: Prostate Specific Antigen
RBC: red blood cell count
RDW: red cell distribution width
RECORD: REporting of studies Conducted using Observational Routinely-collected health Data
SD: standard deviation
SNOMED-CT: Systematized Nomenclature of Medicine-Clinical Terms
STROBE: STrengthening the Reporting of OBservational studies in Epidemiology
UWL: unexpected weight loss
WBC: white blood cell count
