# Supplementary material for: Blood test trend versus single threshold abnormality to discriminate cancer from non-cancer in patients with unexpected weight loss: A retrospective cohort study
Source: PLoS Med. 2026 Sep 17;23(9):e1004956. doi: 10.1371/journal.pmed.1004956 (PMC13585255; doi:10.1371/journal.pmed.1004956)
Supplement: Supplementary file 1 [file pmed.1004956.s001.docx]

# Table A: Summary of blood test abnormality

| Blood level | Abnormality | Abnormality definition | N (%) with a co-occurring test | N (%) with abnormality on occurring test |
| --- | --- | --- | --- | --- |
| Albumin (g/L) | Low | <35 | 90,462 (32.9%) | 8,600 (3.1%) |
| Alkaline phosphatase (iu/L) | High | >130 | 95,110 (34.6%) | 12,279 (4.5%) |
| ALT (iu/L) | High | >35 | 83,860 (30.5%) | 9,344 (3.4%) |
| AST (iu/L) | High | >35 | 15,925 (5.8%) | 1,648 (0.6%) |
| Bilirubin (umol/L) | High | >17 | 93,732 (34.1%) | 8,535 (3.1%) |
| Red blood cell count (10^12^/L) | Low | <4.5 (men)  <3.9 (women) | 100,905 (36.7%) | 34,156 (12.4%) |
| Haemoglobin (g/L) | Low | <130 (men)  <115 (women) | 105,183 (38.2%) | 23,919 (8.7%) |
| Haematocrit (L/L) | Low | <0.40 (men)  <0.37 (women) | 102,122 (37.1%) | 18,187 (6.6%) |
| MCH (pg) | Low | <27 | 102,734 (37.3%) | 1,477 (0.5%) |
| MCHC (g/L) | Low | <300 | 97,153 (35.3%) | 8,483 (3.1%) |
| MCV (fL) | Low | <76 | 75,449 (27.4%) | 4,473 (1.6%) |
| Red cell distribution width (%) | High | >14.6 | 49,343 (17.9%) | 13,488 (4.9%) |
| Platelets (10^9^/L) | High | >400 | 102,575 (37.3%) | 7,948 (2.9%) |
| MPV (fL) | High | >11.5 | 14,143 (5.1%) | 1,120 (0.4%) |
| WBC (10^9^/L) | High | >11 | 103,253 (37.5%) | 8,408 (3.1%) |
| Basophil count (10^9^/L) | High | >0.1 | 95,235 (34.6%) | 0 (0.0%) |
| Eosinophil count (10^9^/L) | High | >0.44 | 100,711 (36.6%) | 5,519 (2.0%) |
| Lymphocyte count (10^9^/L) | Low | <1 | 101,087 (36.7%) | 7,173 (2.6%) |
| Monocyte count (10^9^/L) | High | >0.8 | 100,385 (36.5%) | 11,304 (4.1%) |
| Neutrophil count (10^9^/L) | High | >7.5 | 100,899 (36.7%) | 9,702 (3.5%) |
| CRP (mg/L) | High | >=10 | 41,852 (15.2%) | 21,241 (7.7%) |
| ESR (mm/h) | High | >=10 (men, age <65y)  >=20 (men, age >=65y)  >=15 (women, age <65y)  >=25 (women, age >=65y) | 41,852 (15.2%) | 14,229 (5.2%) |
| Plasma viscosity (mPa.S) | High | >1.72 | 6,055 (2.2%) | 1,909 (0.7%) |
| Neutrophil-to-lymphocyte score | 1 if one test abnormal  2 if both abnormal | Neutrophils >7.5 (10^9^/L)  Lymphocytes <1 (10^9^/L) | 98,984 (36.0%) | 33,102 (12.0%) |
| Platelets-to-lymphocyte score | 1 if one test abnormal  2 if both abnormal | Platelets >400 (10^9^/L)  Lymphocytes <1 (10^9^/L) | 98,149 (35.7%) | 33,074 (12.0%) |
| mGPS score | 1 if CRP alone abnormal  2 if both abnormal | CRP >=10 (mg/L)  Albumin <35 (g/L) | 32,049 (11.6%) | 12,967 (4.7%) |
| Blood test references ranges used to define abnormality were taken from: Oxford Academic. Oxford Handbook of Clinical Medicine - Reference intervals, etc. 2017 [Accessed 21 January 2026].  Abbreviations: ALT=alanine aminotransferase; AST=aspartate aminotransferase; MCH=mean cell haemoglobin; MCHC=mean cell haemoglobin concentration; MCV=mean cell volume; MPV=mean platelet volume; WBC=white blood cell count; CRP=C-reactive protein; ESR=erythrocyte sedimentation rate. | | | | |

# Table B: Sample size calculation

| Blood test | No. of cancer-free patients divided by no. of cancer patients | Null AUC | Target AUC | Alpha | Power | Estimated sample size |
| --- | --- | --- | --- | --- | --- | --- |
| Albumin | 14.176 | 0.5 | 0.7 | 0.05 | 0.8 | 258 |
| Albumin | 14.176 | 0.5 | 0.7 | 0.00048 | 0.8 | 608 |
| Albumin | 14.176 | 0.5 | 0.7 | 0.00048 | 0.9 | 744 |
| Albumin | 14.176 | 0.5 | 0.7 | 0.00048 | 0.99 | 1093 |
|  |  |  |  |  |  |  |
| AST | 13.876 | 0.5 | 0.7 | 0.05 | 0.8 | 253 |
| AST | 13.876 | 0.5 | 0.7 | 0.00048 | 0.8 | 596 |
| AST | 13.876 | 0.5 | 0.7 | 0.00048 | 0.9 | 729 |
| AST | 13.876 | 0.5 | 0.7 | 0.00048 | 0.99 | 1089 |
|  |  |  |  |  |  |  |
| Haemoglobin | 14.613 | 0.5 | 0.7 | 0.05 | 0.8 | 266 |
| Haemoglobin | 14.613 | 0.5 | 0.7 | 0.00048 | 0.8 | 625 |
| Haemoglobin | 14.613 | 0.5 | 0.7 | 0.00048 | 0.9 | 766 |
| Haemoglobin | 14.613 | 0.5 | 0.7 | 0.00048 | 0.99 | 1125 |
|  |  |  |  |  |  |  |
| CRP | 13.801 | 0.5 | 0.7 | 0.05 | 0.8 | 252 |
| CRP | 13.801 | 0.5 | 0.7 | 0.00048 | 0.8 | 593 |
| CRP | 13.801 | 0.5 | 0.7 | 0.00048 | 0.9 | 726 |
| CRP | 13.801 | 0.5 | 0.7 | 0.00048 | 0.99 | 1081 |
|  |  |  |  |  |  |  |
| Plasma viscosity | 13.449 | 0.5 | 0.7 | 0.05 | 0.8 | 246 |
| Plasma viscosity | 13.449 | 0.5 | 0.7 | 0.00048 | 0.8 | 578 |
| Plasma viscosity | 13.449 | 0.5 | 0.7 | 0.00048 | 0.9 | 708 |
| Plasma viscosity | 13.449 | 0.5 | 0.7 | 0.00048 | 0.99 | 1055 |
| A sample size calculation for a difference in AUC was performed for these five blood tests as examples, with AST and plasma viscosity cohorts being the smallest cohorts included in the 1-year trend window and 2+ blood test cohort. A conventional alpha of 0.05 and a Bonferroni-corrected (for multiple testing) alpha of 0.00048 were used alongside power ranging 0.8-0.99 to assess the range of sample size requirements, given the number of tests and large sample size.  Abbreviations: AST=aspartate aminotransferase; CRP=C-reactive protein. | | | | | | |

# Table C: Number of patients with repeat blood tests over each longitudinal window before UWL (n=275,205)

| Blood test | N (%) with at least one test among all UWL patients | | | | N (%) with at least one test among all UWL patients with 2+ repeat tests | | | |
| --- | --- | --- | --- | --- | --- | --- | --- | --- |
|  | **1-year trend** | **3-year trend** | **5-year trend** | **10-year trend** | **1-year trend** | **3-year trend** | **5-year trend** | **10-year trend** |
| Albumin (g/L) | 165,956 (60.3%) | 213,739 (77.7%) | 227,409 (82.6%) | 236,931 (86.1%) | 66,735 (24.2%) | 143,421 (52.1%) | 166,011 (60.3%) | 181,679 (66.0%) |
| ALP (iu/L) | 174,301 (63.3%) | 223,859 (81.3%) | 238,072 (86.5%) | 248,004 (90.1%) | 69,579 (25.3%) | 150,214 (54.6%) | 174,277 (63.3%) | 191,482 (69.6%) |
| ALT (iu/L) | 155,377 (56.5%) | 199,620 (72.5%) | 211,975 (77.0%) | 219,691 (79.8%) | 59,689 (21.7%) | 132,010 (48.0%) | 153,004 (55.6%) | 167,087 (60.7%) |
| AST (iu/L) | 30,627 (11.1%) | 45,293 (16.5%) | 54,379 (19.8%) | 69,346 (25.2%) | 10,891 (4.0%) | 25,226 (9.2%) | 32,533 (11.8%) | 43,772 (15.9%) |
| Bilirubin (umol/L) | 173,641 (63.1%) | 223,797 (81.3%) | 237,994 (86.5%) | 247,190 (89.8%) | 66,831 (24.3%) | 148,187 (53.8%) | 172,558 (62.7%) | 189,413 (68.8%) |
| RBC (10^12^/L) | 175,793 (63.9%) | 228,995 (83.2%) | 244,172 (88.7%) | 254,045 (92.3%) | 74,774 (27.2%) | 155,326 (56.4%) | 182,134 (66.2%) | 201,144 (73.1%) |
| Haemoglobin (g/dL) | 182,440 (66.3%) | 237,657 (86.4%) | 253,426 (92.1%) | 264,012 (95.9%) | 78,373 (28.5%) | 162,397 (59.0%) | 190,473 (69.2%) | 211,083 (76.7%) |
| Haematocrit (L/L) | 178,206 (64.8%) | 231,999 (84.3%) | 247,100 (89.8%) | 256,638 (93.3%) | 75,669 (27.5%) | 157,344 (57.2%) | 184,343 (67.0%) | 203,122 (73.8%) |
| MCV (fL) | 179,511 (65.2%) | 234,451 (85.2%) | 250,083 (90.9%) | 260,353 (94.6%) | 76,090 (27.6%) | 159,159 (57.8%) | 186,960 (67.9%) | 207,016 (75.2%) |
| MCH (pg) | 170,063 (61.8%) | 222,688 (80.9%) | 237,986 (86.5%) | 249,019 (90.5%) | 71,770 (26.1%) | 150,042 (54.5%) | 176,405 (64.1%) | 195,926 (71.2%) |
| MCHC (g/L) | 133,377 (48.5%) | 176,740 (64.2%) | 191,009 (69.4%) | 202,779 (73.7%) | 55,986 (20.3%) | 117,792 (42.8%) | 139,430 (50.7%) | 157,002 (57.0%) |
| RDW (%) | 85,832 (31.2%) | 110,734 (40.2%) | 117,881 (42.8%) | 122,702 (44.6%) | 36,734 (13.3%) | 74,457 (27.1%) | 86,213 (31.3%) | 93,867 (34.1%) |
| Platelets (10^9^/L) | 180,327 (65.5%) | 235,772 (85.7%) | 251,552 (91.4%) | 261,985 (95.2%) | 76,139 (27.7%) | 160,112 (58.2%) | 188,231 (68.4%) | 208,548 (75.8%) |
| MPV (fL) | 26,161 (9.5%) | 35,861 (13.0%) | 39,719 (14.4%) | 43,257 (15.7%) | 10,767 (3.9%) | 23,369 (8.5%) | 28,290 (10.3%) | 32,703 (11.9%) |
| WBC (10^9^/L) | 180,963 (65.8%) | 236,179 (85.8%) | 251,830 (91.5%) | 262,153 (95.3%) | 76,738 (27.9%) | 160,536 (58.3%) | 188,536 (68.5%) | 208,742 (75.8%) |
| Basophil count (10^9^/L) | 167,168 (60.7%) | 218,832 (79.5%) | 233,773 (84.9%) | 243,768 (88.6%) | 69,807 (25.4%) | 146,026 (53.1%) | 171,537 (62.3%) | 189,689 (68.9%) |
| Eosinophil count (10^9^/L) | 176,435 (64.1%) | 229,984 (83.6%) | 244,851 (89.0%) | 254,013 (92.3%) | 74,400 (27.0%) | 155,493 (56.5%) | 182,221 (66.2%) | 200,523 (72.9%) |
| Lymphocyte count (10^9^/L) | 177,201 (64.4%) | 230,857 (83.9%) | 245,756 (89.3%) | 255,059 (92.7%) | 74,690 (27.1%) | 156,211 (56.8%) | 183,078 (66.5%) | 201,586 (73.2%) |
| Monocyte count (10^9^/L) | 176,423 (64.1%) | 229,998 (83.6%) | 244,845 (89.0%) | 254,054 (92.3%) | 74,269 (27.0%) | 155,544 (56.5%) | 182,352 (66.3%) | 200,658 (72.9%) |
| Neutrophil count (10^9^/L) | 177,337 (64.4%) | 231,071 (84.0%) | 245,900 (89.4%) | 255,158 (92.7%) | 74,749 (27.2%) | 156,339 (56.8%) | 183,170 (66.6%) | 201,598 (73.3%) |
| CRP (mg/L) | 75,108 (27.3%) | 115,443 (41.9%) | 135,621 (49.3%) | 156,355 (56.8%) | 18,043 (6.6%) | 45,880 (16.7%) | 64,614 (23.5%) | 87,544 (31.8%) |
| ESR (mm/h) | 75,108 (27.3%) | 115,443 (41.9%) | 135,621 (49.3%) | 156,355 (56.8%) | 18,043 (6.6%) | 45,880 (16.7%) | 64,614 (23.5%) | 87,544 (31.8%) |
| Plasma viscosity (mPa.S) | 10,956 (4.0%) | 17,526 (6.4%) | 21,579 (7.8%) | 27,022 (9.8%) | 2,543 (0.9%) | 6,516 (2.4%) | 9,433 (3.4%) | 13,579 (4.9%) |
| NLR | 175,413 (63.7%) | 229,463 (83.4%) | 244,476 (88.8%) | 253,850 (92.2%) | 73,096 (26.6%) | 154,482 (56.1%) | 181,453 (65.9%) | 200,013 (72.7%) |
| PLR | 174,445 (63.4%) | 228,654 (83.1%) | 243,792 (88.6%) | 253,283 (92.0%) | 72,436 (26.3%) | 153,837 (55.9%) | 180,858 (65.7%) | 199,552 (72.5%) |
| mGPR | 58,171 (21.1%) | 90,170 (32.8%) | 106,401 (38.7%) | 122,882 (44.7%) | 10,509 (3.8%) | 29,116 (10.6%) | 42,424 (15.4%) | 58,387 (21.2%) |
| The left-hand panel reports the number and proportion of patients in the total UWL cohort who had at least one of each type of blood test in the trend window. The right-hand panel reports this for the subset of patients with at least two blood tests in the trend window, which were the patients included in the trend analysis.  Abbreviations: UWL=unexpected weight loss; ALP=alkaline phosphatase; ALT=alanine aminotransferase; AST=aspartate aminotransferase; RBC=red blood cell count; MCH=mean cell haemoglobin; MCHC=mean cell haemoglobin concentration; MCV=mean cell volume; RDW=red cell distribution width; MPV=mean platelet volume; WBC=white blood cell count; CRP=C-reactive protein; ESR=erythrocyte sedimentation rate; NLR=neutrophil-lymphocyte ratio; PLR=platelet-lymphocyte ratio; mGPR=modified Glasgow Prognostic ratio. | | | | | | | | |

# Figure A: Summary of time between first and last blood test (top) and last blood test and UWL (bottom)

The first and last test per patient over the 10-year period prior to UWL were extracted and the time difference between them calculated and summarised. The time from UWL to cancer diagnosis is also summarised.

Abbreviations: UWL=unexpected weight loss; SD=standard deviation; LQ=lower quartile; UQ=upper quartile.

# Figure B: Albumin over time by age at UWL and sex: males (left) and females (right)

Legend: LOWESS trends are presented, which represent the observed population-level trend in the study sample. The overlaid grey box is the reference range used in clinical practice.

Abbreviations: UWL=unexpected weight loss.

# Figure C: Alkaline phosphatase over time by age at UWL and sex: males (left) and females (right)

Legend: LOWESS trends are presented, which represent the observed population-level trend in the study sample. The overlaid grey box is the reference range used in clinical practice.

Abbreviations: UWL=unexpected weight loss.

# Figure D: ALT over time by age at UWL and sex: males (left) and females (right)

Legend: LOWESS trends are presented, which represent the observed population-level trend in the study sample. The overlaid grey box is the reference range used in clinical practice.

Abbreviations: UWL=unexpected weight loss; ALT=alanine aminotransferase.

# Figure E: AST over time by age at UWL and sex: males (left) and females (right)

Legend: LOWESS trends are presented, which represent the observed population-level trend in the study sample. The overlaid grey box is the reference range used in clinical practice.

Abbreviations: UWL=unexpected weight loss; AST=aspartate aminotransferase.

# Figure F: Bilirubin over time by age at UWL and sex: males (left) and females (right)

Legend: LOWESS trends are presented, which represent the observed population-level trend in the study sample. The overlaid grey box is the reference range used in clinical practice.

Abbreviations: UWL=unexpected weight loss.

# Figure G: Haemoglobin over time by age at UWL and sex: males (left) and females (right)

Legend: LOWESS trends are presented, which represent the observed population-level trend in the study sample. The overlaid grey box is the reference range used in clinical practice.

Abbreviations: UWL=unexpected weight loss.

# Figure H: Lymphocyte count over time by age at UWL and sex: males (left) and females (right)

Legend: LOWESS trends are presented, which represent the observed population-level trend in the study sample. The overlaid grey box is the reference range used in clinical practice.

Abbreviations: UWL=unexpected weight loss.

# Figure I: MCV over time by age at UWL and sex: males (left) and females (right)

Legend: LOWESS trends are presented, which represent the observed population-level trend in the study sample. The overlaid grey box is the reference range used in clinical practice.

Abbreviations: UWL=unexpected weight loss; MCV=mean cell volume.

# Figure J: Monocyte count over time by age at UWL and sex: males (left) and females (right)

Legend: LOWESS trends are presented, which represent the observed population-level trend in the study sample. The overlaid grey box is the reference range used in clinical practice.

Abbreviations: UWL=unexpected weight loss.

# Figure K: Neutrophil count over time by age at UWL and sex: males (left) and females (right)

Legend: LOWESS trends are presented, which represent the observed population-level trend in the study sample. The overlaid grey box is the reference range used in clinical practice.

Abbreviations: UWL=unexpected weight loss.

# Figure L: Platelets over time by age at UWL and sex: males (left) and females (right)

Legend: LOWESS trends are presented, which represent the observed population-level trend in the study sample. The overlaid grey box is the reference range used in clinical practice.

Abbreviations: UWL=unexpected weight loss.

# Figure M: White blood cell count over time by age at UWL and sex: males (left) and females (right)

Legend: LOWESS trends are presented, which represent the observed population-level trend in the study sample. The overlaid grey box is the reference range used in clinical practice.

Abbreviations: UWL=unexpected weight loss.

# Figure N: C-reactive protein over time by age at UWL and sex: males (left) and females (right)

Legend: LOWESS trends are presented, which represent the observed population-level trend in the study sample. The overlaid grey box is the reference range used in clinical practice.

Abbreviations: UWL=unexpected weight loss.

# Figure O: Erythrocyte sedimentation rate over time by age at UWL and sex: males (left) and females (right)

Legend: LOWESS trends are presented, which represent the observed population-level trend in the study sample. The overlaid grey box is the reference range used in clinical practice.

Abbreviations: UWL=unexpected weight loss.

# Figure P: Red blood cell count over time by age at UWL and sex: males (left) and females (right)

Legend: LOWESS trends are presented, which represent the observed population-level trend in the study sample. The overlaid grey box is the reference range used in clinical practice.

Abbreviations: UWL=unexpected weight loss.

# Figure Q: Haematocrit over time by age at UWL and sex: males (left) and females (right)

Legend: the overlaid grey box is the normal/reference range

Abbreviations: UWL=unexpected weight loss.

# Figure R: Mean cell haemoglobin over time by age at UWL and sex: males (left) and females (right)

Legend: LOWESS trends are presented, which represent the observed population-level trend in the study sample. The overlaid grey box is the reference range used in clinical practice.

Abbreviations: UWL=unexpected weight loss.

# Figure S: Mean cell haemoglobin concentration over time by age at UWL and sex: males (left) and females (right)

Legend: LOWESS trends are presented, which represent the observed population-level trend in the study sample. The overlaid grey box is the reference range used in clinical practice.

Abbreviations: UWL=unexpected weight loss.

# Figure T: Red blood cell distribution width over time by age at UWL and sex: males (left) and females (right)

Legend: LOWESS trends are presented, which represent the observed population-level trend in the study sample. The overlaid grey box is the reference range used in clinical practice.

Abbreviations: UWL=unexpected weight loss.

# Figure U: Mean platelet volume over time by age at UWL and sex: males (left) and females (right)

Legend: LOWESS trends are presented, which represent the observed population-level trend in the study sample. The overlaid grey box is the reference range used in clinical practice.

Abbreviations: UWL=unexpected weight loss.

# Figure V: Basophil count over time by age at UWL and sex: males (left) and females (right)

Legend: LOWESS trends are presented, which represent the observed population-level trend in the study sample. The overlaid grey box is the reference range used in clinical practice.

Abbreviations: UWL=unexpected weight loss.

# Figure W: Eosinophil count over time by age at UWL and sex: males (left) and females (right)

Legend: LOWESS trends are presented, which represent the observed population-level trend in the study sample. The overlaid grey box is the reference range used in clinical practice.

Abbreviations: UWL=unexpected weight loss.

# Figure X: Plasma viscosity over time by age at UWL and sex: males (left) and females (right)

Legend: LOWESS trends are presented, which represent the observed population-level trend in the study sample. The overlaid grey box is the reference range used in clinical practice.

Abbreviations: UWL=unexpected weight loss.

# Figure Y: Neutrophil-to-lymphocyte ratio over time by age at UWL and sex: males (left) and females (right)

Legend: LOWESS trends are presented, which represent the observed population-level trend in the study sample.

Abbreviations: UWL=unexpected weight loss.

# Figure Z: Platelet-to-lymphocyte ratio over time by age at UWL and sex: males (left) and females (right)

Legend: LOWESS trends are presented, which represent the observed population-level trend in the study sample.

Abbreviations: UWL=unexpected weight loss.

# Figure AA: mGPR (C-reactive protein-to-albumin ratio) over time by age at UWL and sex: males (left) and females (right)

Legend: LOWESS trends are presented, which represent the observed population-level trend in the study sample.

Abbreviations: UWL=unexpected weight loss; mGPR=modified Glasgow Prognostic ratio.

# Table D: AUC (95% CI) of blood test trend and abnormality for 6-month cancer risk following UWL

| Blood test | Model | Abnormality | 1-year Trend | 3-year Trend | 5-year Trend | 10-year Trend |
| --- | --- | --- | --- | --- | --- | --- |
| Albumin (g/L) declining | Unadjusted | 0.55 (0.54-0.56) | 0.58 (0.57-0.59) | 0.56 (0.55-0.57) | 0.55 (0.54-0.56) | 0.54 (0.53-0.55) |
| Albumin (g/L) declining | Adjusted | 0.66 (0.66-0.67) | 0.67 (0.66-0.68) | 0.68 (0.67-0.68) | 0.68 (0.67-0.68) | 0.67 (0.67-0.68) |
| Alkaline phosphatase (iu/L) increasing | Unadjusted | 0.56 (0.55-0.56) | 0.59 (0.58-0.60) | 0.58 (0.56-0.59) | 0.57 (0.55-0.58) | 0.57 (0.55-0.58) |
| Alkaline phosphatase (iu/L) increasing | Adjusted | 0.66 (0.65-0.67) | 0.67 (0.66-0.68) | 0.68 (0.67-0.68) | 0.68 (0.67-0.69) | 0.68 (0.67-0.69) |
| ALT (iu/L) declining | Unadjusted | 0.50 (0.50-0.51) | 0.52 (0.51-0.53) | 0.52 (0.51-0.53) | 0.51 (0.50-0.52) | 0.50 (0.49-0.51) |
| ALT (iu/L) declining | Adjusted | 0.64 (0.63-0.65) | 0.64 (0.63-0.65) | 0.66 (0.65-0.67) | 0.66 (0.66-0.67) | 0.66 (0.66-0.67) |
| AST (iu/L) declining | Unadjusted | 0.52 (0.51-0.53) | 0.52 (0.50-0.54) | 0.52 (0.51-0.54) | 0.51 (0.50-0.53) | 0.51 (0.50-0.52) |
| AST (iu/L) declining | Adjusted | 0.66 (0.64-0.68) | 0.66 (0.63-0.68) | 0.67 (0.65-0.68) | 0.67 (0.65-0.68) | 0.67 (0.66-0.69) |
| Bilirubin (umol/L) declining | Unadjusted | 0.50 (0.50-0.51) | 0.54 (0.53-0.55) | 0.52 (0.51-0.53) | 0.53 (0.52-0.54) | 0.52 (0.51-0.53) |
| Bilirubin (umol/L) declining | Adjusted | 0.64 (0.64-0.65) | 0.65 (0.64-0.65) | 0.66 (0.65-0.67) | 0.66 (0.66-0.67) | 0.66 (0.66-0.67) |
| RBC (10^12^/L) declining | Unadjusted | 0.57 (0.56-0.57) | 0.50 (0.49-0.51) | 0.51 (0.49-0.52) | 0.50 (0.49-0.51) | 0.51 (0.49-0.52) |
| RBC (10^12^/L) declining | Adjusted | 0.65 (0.65-0.66) | 0.65 (0.64-0.66) | 0.67 (0.66-0.67) | 0.67 (0.67-0.68) | 0.67 (0.67-0.68) |
| Haemoglobin (g/L) declining | Unadjusted | 0.59 (0.59-0.60) | 0.57 (0.56-0.58) | 0.56 (0.55-0.57) | 0.53 (0.52-0.55) | 0.54 (0.53-0.55) |
| Haemoglobin (g/L) declining | Adjusted | 0.67 (0.67-0.68) | 0.67 (0.67-0.68) | 0.68 (0.68-0.69) | 0.68 (0.68-0.69) | 0.68 (0.68-0.69) |
| Haematocrit (L/L) declining | Unadjusted | 0.56 (0.55-0.57) | 0.51 (0.50-0.52) | 0.53 (0.52-0.54) | 0.51 (0.49-0.52) | 0.52 (0.51-0.53) |
| Haematocrit (L/L) declining | Adjusted | 0.67 (0.66-0.68) | 0.65 (0.64-0.66) | 0.67 (0.66-0.67) | 0.67 (0.66-0.68) | 0.67 (0.67-0.68) |
| MCH (pg) declining | Unadjusted | 0.54 (0.54-0.55) | 0.59 (0.58-0.60) | 0.57 (0.56-0.58) | 0.57 (0.55-0.58) | 0.56 (0.54-0.57) |
| MCH (pg) declining | Adjusted | 0.68 (0.67-0.68) | 0.68 (0.68-0.69) | 0.69 (0.68-0.70) | 0.69 (0.68-0.70) | 0.69 (0.68-0.69) |
| MCHC (g/L) declining | Unadjusted | 0.50 (0.50-0.50) | 0.55 (0.54-0.56) | 0.54 (0.53-0.55) | 0.54 (0.53-0.55) | 0.52 (0.51-0.53) |
| MCHC (g/L) declining | Adjusted | 0.65 (0.64-0.66) | 0.65 (0.64-0.66) | 0.67 (0.66-0.68) | 0.67 (0.67-0.68) | 0.68 (0.67-0.68) |
| MCV (fL) declining | Unadjusted | 0.51 (0.51-0.51) | 0.58 (0.57-0.60) | 0.57 (0.56-0.58) | 0.55 (0.53-0.56) | 0.54 (0.53-0.55) |
| MCV (fL) declining | Adjusted | 0.66 (0.65-0.67) | 0.68 (0.67-0.69) | 0.69 (0.68-0.69) | 0.69 (0.68-0.69) | 0.69 (0.68-0.69) |
| RDW (%) increasing | Unadjusted | 0.55 (0.54-0.56) | 0.55 (0.53-0.56) | 0.54 (0.53-0.56) | 0.54 (0.53-0.56) | 0.53 (0.52-0.54) |
| RDW (%) increasing | Adjusted | 0.65 (0.64-0.67) | 0.65 (0.64-0.66) | 0.67 (0.66-0.68) | 0.67 (0.66-0.68) | 0.67 (0.67-0.68) |
| Platelets (10^9^/L) increasing | Unadjusted | 0.55 (0.54-0.55) | 0.59 (0.58-0.60) | 0.57 (0.56-0.58) | 0.57 (0.55-0.58) | 0.57 (0.56-0.58) |
| Platelets (10^9^/L) increasing | Adjusted | 0.68 (0.68-0.69) | 0.69 (0.68-0.70) | 0.70 (0.69-0.70) | 0.69 (0.69-0.70) | 0.69 (0.69-0.70) |
| MPV (fL) declining | Unadjusted | 0.52 (0.51-0.52) | 0.54 (0.52-0.56) | 0.52 (0.51-0.54) | 0.53 (0.51-0.54) | 0.52 (0.50-0.53) |
| MPV (fL) declining | Adjusted | 0.66 (0.64-0.68) | 0.67 (0.65-0.69) | 0.67 (0.66-0.69) | 0.68 (0.66-0.70) | 0.68 (0.66-0.70) |
| WBC (10^9^/L) increasing | Unadjusted | 0.54 (0.54-0.55) | 0.60 (0.59-0.61) | 0.59 (0.58-0.60) | 0.58 (0.56-0.59) | 0.58 (0.57-0.60) |
| WBC (10^9^/L) increasing | Adjusted | 0.67 (0.66-0.68) | 0.68 (0.68-0.69) | 0.69 (0.69-0.70) | 0.69 (0.69-0.70) | 0.69 (0.69-0.70) |
| Basophil count (10^9^/L) increasing | Unadjusted | 0.51 (0.50-0.52) | 0.51 (0.49-0.52) | 0.50 (0.49-0.51) | 0.51 (0.50-0.53) | 0.51 (0.49-0.52) |
| Basophil count (10^9^/L) increasing | Adjusted | 0.65 (0.64-0.66) | 0.65 (0.64-0.66) | 0.67 (0.66-0.67) | 0.67 (0.66-0.68) | 0.67 (0.67-0.68) |
| Eosinophil count (10^9^/L) increasing | Unadjusted | 0.50 (0.50-0.51) | 0.50 (0.49-0.52) | 0.50 (0.49-0.52) | 0.50 (0.49-0.51) | 0.50 (0.49-0.51) |
| Eosinophil count (10^9^/L) increasing | Adjusted | 0.65 (0.64-0.66) | 0.65 (0.64-0.66) | 0.67 (0.66-0.67) | 0.67 (0.67-0.68) | 0.67 (0.67-0.68) |
| Lymphocyte count (10^9^/L) increasing | Unadjusted | 0.52 (0.51-0.52) | 0.51 (0.50-0.52) | 0.52 (0.51-0.53) | 0.52 (0.51-0.53) | 0.53 (0.52-0.54) |
| Lymphocyte count (10^9^/L) increasing | Adjusted | 0.65 (0.65-0.66) | 0.65 (0.65-0.66) | 0.67 (0.66-0.67) | 0.67 (0.67-0.68) | 0.67 (0.67-0.68) |
| Monocyte count (10^9^/L) increasing | Unadjusted | 0.58 (0.57-0.58) | 0.58 (0.57-0.59) | 0.56 (0.55-0.58) | 0.57 (0.56-0.58) | 0.56 (0.55-0.57) |
| Monocyte count (10^9^/L) increasing | Adjusted | 0.68 (0.67-0.68) | 0.68 (0.67-0.68) | 0.69 (0.68-0.69) | 0.69 (0.68-0.69) | 0.69 (0.68-0.69) |
| Neutrophil count (10^9^/L) increasing | Unadjusted | 0.55 (0.55-0.56) | 0.60 (0.59-0.62) | 0.59 (0.57-0.60) | 0.58 (0.57-0.59) | 0.57 (0.56-0.59) |
| Neutrophil count (10^9^/L) increasing | Adjusted | 0.68 (0.67-0.69) | 0.69 (0.68-0.70) | 0.69 (0.69-0.70) | 0.69 (0.69-0.70) | 0.69 (0.68-0.70) |
| CRP (mg/L) increasing | Unadjusted | 0.59 (0.58-0.60) | 0.63 (0.61-0.64) | 0.61 (0.60-0.62) | 0.60 (0.59-0.61) | 0.59 (0.57-0.60) |
| CRP (mg/L) increasing | Adjusted | 0.68 (0.66-0.69) | 0.70 (0.69-0.72) | 0.71 (0.70-0.72) | 0.71 (0.70-0.72) | 0.71 (0.70-0.72) |
| ESR (mm/h) increasing | Unadjusted | 0.62 (0.61-0.63) | 0.63 (0.61-0.65) | 0.61 (0.60-0.62) | 0.60 (0.59-0.61) | 0.59 (0.57-0.60) |
| ESR (mm/h) increasing | Adjusted | 0.69 (0.68-0.71) | 0.70 (0.69-0.72) | 0.71 (0.70-0.72) | 0.71 (0.70-0.73) | 0.71 (0.70-0.72) |
| Plasma viscosity (mPa.S) increasing | Unadjusted | 0.63 (0.60-0.67) | 0.65 (0.61-0.70) | 0.60 (0.57-0.64) | 0.59 (0.56-0.62) | 0.58 (0.56-0.60) |
| Plasma viscosity (mPa.S) increasing | Adjusted | 0.71 (0.66-0.75) | 0.70 (0.66-0.75) | 0.69 (0.66-0.73) | 0.70 (0.67-0.73) | 0.71 (0.68-0.74) |
| NLR increasing | Unadjusted | 0.55 (0.54-0.55) | 0.57 (0.56-0.58) | 0.55 (0.54-0.56) | 0.55 (0.53-0.56) | 0.52 (0.51-0.54) |
| NLR increasing | Adjusted | 0.66 (0.66-0.67) | 0.67 (0.67-0.68) | 0.68 (0.67-0.69) | 0.68 (0.68-0.69) | 0.68 (0.67-0.69) |
| PLR increasing | Unadjusted | 0.55 (0.54-0.56) | 0.57 (0.55-0.59) | 0.54 (0.53-0.55) | 0.53 (0.51-0.54) | 0.51 (0.50-0.52) |
| PLR increasing | Adjusted | 0.67 (0.66-0.68) | 0.68 (0.67-0.69) | 0.69 (0.68-0.69) | 0.68 (0.68-0.69) | 0.68 (0.68-0.69) |
| mGPR increasing | Unadjusted | 0.55 (0.53-0.56) | 0.62 (0.59-0.64) | 0.60 (0.58-0.62) | 0.59 (0.58-0.60) | 0.58 (0.57-0.59) |
| mGPR increasing | Adjusted | 0.66 (0.63-0.68) | 0.69 (0.67-0.71) | 0.70 (0.69-0.72) | 0.71 (0.69-0.72) | 0.71 (0.69-0.72) |
| This table presents the AUC of unadjusted and age- and sex-adjusted blood test abnormality and trend from each trend-window.  Abbreviations: UWL=unexpected weight loss: ALT=alanine aminotransferase; AST=aspartate aminotransferase; RBC=red blood cell count; MCH=mean cell haemoglobin; MCHC=mean cell haemoglobin concentration; MCV=mean cell volume; RDW=red cell distribution width; MPV=mean platelet volume; WBC=white blood cell count; CRP=C-reactive protein; ESR=erythrocyte sedimentation rate; NLR=neutrophil-lymphocyte ratio; PLR=platelet-lymphocyte ratio; mGPR=modified Glasgow Prognostic ratio. | | | | | | |

# Table E: Hazard ratio (95% CI) of blood test abnormality and trend for increased six-month cancer risk following UWL – rescaled to reflect clinically meaningful changes in the blood test value

| Blood test | Model | Rescale to changes in test values of # units | 1-year Trend | 3-year Trend | 5-year Trend | 10-year Trend |
| --- | --- | --- | --- | --- | --- | --- |
| Albumin (g/L) declining | Unadjusted | 1 | 1.076 | 1.062 | 1.053 | 1.044 |
| Albumin (g/L) declining | Unadjusted | 5 | 1.445 | 1.348 | 1.292 | 1.239 |
| Albumin (g/L) declining | Unadjusted | 10 | 2.089 | 1.818 | 1.670 | 1.536 |
| Albumin (g/L) declining | Adjusted | 1 | 1.081 | 1.074 | 1.067 | 1.054 |
| Albumin (g/L) declining | Adjusted | 5 | 1.477 | 1.430 | 1.385 | 1.299 |
| Albumin (g/L) declining | Adjusted | 10 | 2.181 | 2.044 | 1.917 | 1.688 |
| Alkaline phosphatase (iu/L) increasing | Unadjusted | 1 | 1.006 | 1.006 | 1.005 | 1.005 |
| Alkaline phosphatase (iu/L) increasing | Unadjusted | 5 | 1.030 | 1.030 | 1.025 | 1.025 |
| Alkaline phosphatase (iu/L) increasing | Unadjusted | 10 | 1.062 | 1.062 | 1.051 | 1.051 |
| Alkaline phosphatase (iu/L) increasing | Adjusted | 1 | 1.007 | 1.006 | 1.006 | 1.006 |
| Alkaline phosphatase (iu/L) increasing | Adjusted | 5 | 1.035 | 1.030 | 1.030 | 1.030 |
| Alkaline phosphatase (iu/L) increasing | Adjusted | 10 | 1.072 | 1.062 | 1.062 | 1.062 |
| ALT (iu/L) declining | Unadjusted | 1 | 1.004 | 1.004 | 1.004 | 1.002 |
| ALT (iu/L) declining | Unadjusted | 5 | 1.020 | 1.020 | 1.020 | 1.010 |
| ALT (iu/L) declining | Unadjusted | 10 | 1.041 | 1.041 | 1.041 | 1.020 |
| ALT (iu/L) declining | Adjusted | 1 | 1.005 | 1.002 | 1.004 | 1.000 |
| ALT (iu/L) declining | Adjusted | 5 | 1.025 | 1.010 | 1.020 | 1.000 |
| ALT (iu/L) declining | Adjusted | 10 | 1.051 | 1.020 | 1.041 | 1.000 |
| AST (iu/L) declining | Unadjusted | 1 | 1.002 | 1.006 | 1.003 | 1.004 |
| AST (iu/L) declining | Unadjusted | 5 | 1.010 | 1.031 | 1.015 | 1.020 |
| AST (iu/L) declining | Unadjusted | 10 | 1.020 | 1.062 | 1.031 | 1.041 |
| AST (iu/L) declining | Adjusted | 1 | 1.000 | 1.001 | 1.001 | 1.001 |
| AST (iu/L) declining | Adjusted | 5 | 1.000 | 1.005 | 1.005 | 1.005 |
| AST (iu/L) declining | Adjusted | 10 | 1.000 | 1.010 | 1.010 | 1.010 |
| Bilirubin (umol/L) declining | Unadjusted | 1 | 1.032 | 1.019 | 1.019 | 1.019 |
| Bilirubin (umol/L) declining | Unadjusted | 5 | 1.171 | 1.101 | 1.101 | 1.101 |
| Bilirubin (umol/L) declining | Unadjusted | 10 | 1.370 | 1.211 | 1.211 | 1.211 |
| Bilirubin (umol/L) declining | Adjusted | 1 | 1.020 | 1.022 | 1.022 | 1.021 |
| Bilirubin (umol/L) declining | Adjusted | 5 | 1.106 | 1.118 | 1.118 | 1.112 |
| Bilirubin (umol/L) declining | Adjusted | 10 | 1.224 | 1.249 | 1.249 | 1.236 |
| RBC (10^12^/L) declining | Unadjusted | 1 | 1.004 | 1.040 | 1.020 | 1.022 |
| RBC (10^12^/L) declining | Unadjusted | 5 | 1.020 | 1.214 | 1.106 | 1.115 |
| RBC (10^12^/L) declining | Unadjusted | 10 | 1.041 | 1.473 | 1.224 | 1.243 |
| RBC (10^12^/L) declining | Adjusted | 1 | 1.025 | 1.030 | 1.009 | 1.006 |
| RBC (10^12^/L) declining | Adjusted | 5 | 1.129 | 1.159 | 1.046 | 1.030 |
| RBC (10^12^/L) declining | Adjusted | 10 | 1.275 | 1.342 | 1.094 | 1.062 |
| Haemoglobin (g/L) declining | Unadjusted | 1 | 1.017 | 1.017 | 1.012 | 1.013 |
| Haemoglobin (g/L) declining | Unadjusted | 5 | 1.090 | 1.090 | 1.062 | 1.068 |
| Haemoglobin (g/L) declining | Unadjusted | 10 | 1.187 | 1.187 | 1.128 | 1.140 |
| Haemoglobin (g/L) declining | Adjusted | 1 | 1.017 | 1.015 | 1.014 | 1.014 |
| Haemoglobin (g/L) declining | Adjusted | 5 | 1.090 | 1.078 | 1.073 | 1.073 |
| Haemoglobin (g/L) declining | Adjusted | 10 | 1.187 | 1.163 | 1.151 | 1.151 |
| Haematocrit (L/L) declining | Unadjusted | 1 | 1.001 | 1.002 | 1.000 | 1.006 |
| Haematocrit (L/L) declining | Unadjusted | 5 | 1.005 | 1.010 | 1.000 | 1.031 |
| Haematocrit (L/L) declining | Unadjusted | 10 | 1.010 | 1.020 | 1.000 | 1.062 |
| Haematocrit (L/L) declining | Adjusted | 1 | 1.001 | 1.006 | 1.007 | 1.003 |
| Haematocrit (L/L) declining | Adjusted | 5 | 1.005 | 1.031 | 1.035 | 1.015 |
| Haematocrit (L/L) declining | Adjusted | 10 | 1.010 | 1.062 | 1.072 | 1.031 |
| MCH (pg) declining | Unadjusted | 1 | 1.135 | 1.120 | 1.121 | 1.098 |
| MCH (pg) declining | Unadjusted | 5 | 1.884 | 1.761 | 1.771 | 1.594 |
| MCH (pg) declining | Unadjusted | 10 | 3.550 | 3.101 | 3.136 | 2.540 |
| MCH (pg) declining | Adjusted | 1 | 1.140 | 1.160 | 1.127 | 1.119 |
| MCH (pg) declining | Adjusted | 5 | 1.928 | 2.101 | 1.821 | 1.751 |
| MCH (pg) declining | Adjusted | 10 | 3.715 | 4.415 | 3.317 | 3.066 |
| MCHC (g/L) declining | Unadjusted | 1 | 1.001 | 1.001 | 1.001 | 1.000 |
| MCHC (g/L) declining | Unadjusted | 5 | 1.005 | 1.005 | 1.005 | 1.000 |
| MCHC (g/L) declining | Unadjusted | 10 | 1.010 | 1.010 | 1.010 | 1.000 |
| MCHC (g/L) declining | Adjusted | 1 | 1.001 | 1.000 | 1.001 | 1.001 |
| MCHC (g/L) declining | Adjusted | 5 | 1.005 | 1.000 | 1.005 | 1.005 |
| MCHC (g/L) declining | Adjusted | 10 | 1.010 | 1.000 | 1.010 | 1.010 |
| MCV (fL) declining | Unadjusted | 1 | 1.053 | 1.049 | 1.031 | 1.026 |
| MCV (fL) declining | Unadjusted | 5 | 1.292 | 1.272 | 1.165 | 1.135 |
| MCV (fL) declining | Unadjusted | 10 | 1.670 | 1.618 | 1.356 | 1.288 |
| MCV (fL) declining | Adjusted | 1 | 1.055 | 1.047 | 1.053 | 1.048 |
| MCV (fL) declining | Adjusted | 5 | 1.306 | 1.259 | 1.292 | 1.265 |
| MCV (fL) declining | Adjusted | 10 | 1.706 | 1.585 | 1.670 | 1.601 |
| RDW (%) increasing | Unadjusted | 1 | 1.071 | 1.078 | 1.063 | 1.056 |
| RDW (%) increasing | Unadjusted | 5 | 1.409 | 1.456 | 1.357 | 1.313 |
| RDW (%) increasing | Unadjusted | 10 | 1.986 | 2.119 | 1.842 | 1.724 |
| RDW (%) increasing | Adjusted | 1 | 1.062 | 1.076 | 1.064 | 1.066 |
| RDW (%) increasing | Adjusted | 5 | 1.351 | 1.442 | 1.364 | 1.377 |
| RDW (%) increasing | Adjusted | 10 | 1.825 | 2.080 | 1.860 | 1.895 |
| Platelets (10^9^/L) increasing | Unadjusted | 1 | 1.005 | 1.004 | 1.004 | 1.004 |
| MPV (fL) declining | Unadjusted | 5 | 1.025 | 1.020 | 1.020 | 1.020 |
| MPV (fL) declining | Unadjusted | 10 | 1.051 | 1.041 | 1.041 | 1.041 |
| MPV (fL) declining | Adjusted | 1 | 1.005 | 1.004 | 1.004 | 1.003 |
| MPV (fL) declining | Adjusted | 5 | 1.025 | 1.020 | 1.020 | 1.015 |
| MPV (fL) declining | Adjusted | 10 | 1.051 | 1.041 | 1.041 | 1.030 |
| MPV (fL) declining | Unadjusted | 1 | 1.136 | 1.078 | 1.080 | 1.056 |
| MPV (fL) declining | Unadjusted | 5 | 1.895 | 1.453 | 1.469 | 1.313 |
| MPV (fL) declining | Unadjusted | 10 | 3.591 | 2.111 | 2.157 | 1.724 |
| MPV (fL) declining | Adjusted | 1 | 1.149 | 1.105 | 1.093 | 1.094 |
| MPV (fL) declining | Adjusted | 5 | 2.006 | 1.647 | 1.559 | 1.568 |
| MPV (fL) declining | Adjusted | 10 | 4.025 | 2.713 | 2.431 | 2.458 |
| WBC (10^9^/L) increasing | Unadjusted | 1 | 1.197 | 1.176 | 1.152 | 1.179 |
| WBC (10^9^/L) increasing | Unadjusted | 5 | 2.457 | 2.249 | 2.029 | 2.278 |
| WBC (10^9^/L) increasing | Unadjusted | 10 | 6.039 | 5.059 | 4.116 | 5.190 |
| WBC (10^9^/L) increasing | Adjusted | 1 | 1.221 | 1.194 | 1.178 | 1.161 |
| WBC (10^9^/L) increasing | Adjusted | 5 | 2.714 | 2.427 | 2.268 | 2.109 |
| WBC (10^9^/L) increasing | Adjusted | 10 | 7.365 | 5.889 | 5.146 | 4.450 |
| Basophil count (10^9^/L) increasing | Unadjusted | 0.1 | 1.130 | 1.117 | 1.252 | 1.145 |
| Basophil count (10^9^/L) increasing | Unadjusted | 0.5 | 1.840 | 1.739 | 3.080 | 1.965 |
| Basophil count (10^9^/L) increasing | Unadjusted | 1 | 3.384 | 3.025 | 9.487 | 3.860 |
| Basophil count (10^9^/L) increasing | Adjusted | 0.1 | 1.068 | 1.002 | 1.117 | 1.062 |
| Basophil count (10^9^/L) increasing | Adjusted | 0.5 | 1.391 | 1.009 | 1.740 | 1.351 |
| Basophil count (10^9^/L) increasing | Adjusted | 1 | 1.936 | 1.017 | 3.028 | 1.824 |
| Eosinophil count (10^9^/L) increasing | Unadjusted | 1 | 1.175 | 1.012 | 1.030 | 1.103 |
| Eosinophil count (10^9^/L) increasing | Unadjusted | 5 | 2.240 | 1.062 | 1.159 | 1.633 |
| Eosinophil count (10^9^/L) increasing | Unadjusted | 10 | 5.016 | 1.128 | 1.342 | 2.665 |
| Eosinophil count (10^9^/L) increasing | Adjusted | 1 | 1.513 | 1.460 | 1.613 | 1.321 |
| Eosinophil count (10^9^/L) increasing | Adjusted | 5 | 7.925 | 6.631 | 10.915 | 4.023 |
| Eosinophil count (10^9^/L) increasing | Adjusted | 10 | 62.804 | 43.964 | 119.147 | 16.182 |
| Lymphocyte count (10^9^/L) increasing | Unadjusted | 1 | 1.043 | 1.118 | 1.121 | 1.182 |
| Lymphocyte count (10^9^/L) increasing | Unadjusted | 5 | 1.234 | 1.747 | 1.770 | 2.307 |
| Lymphocyte count (10^9^/L) increasing | Unadjusted | 10 | 1.524 | 3.051 | 3.134 | 5.323 |
| Lymphocyte count (10^9^/L) increasing | Adjusted | 1 | 1.043 | 1.096 | 1.110 | 1.061 |
| Lymphocyte count (10^9^/L) increasing | Adjusted | 5 | 1.233 | 1.581 | 1.685 | 1.345 |
| Lymphocyte count (10^9^/L) increasing | Adjusted | 10 | 1.520 | 2.501 | 2.839 | 1.808 |
| Monocyte count (10^9^/L) increasing | Unadjusted | 0.1 | 1.172 | 1.151 | 1.172 | 1.143 |
| Monocyte count (10^9^/L) increasing | Unadjusted | 0.5 | 2.207 | 2.024 | 2.209 | 1.952 |
| Monocyte count (10^9^/L) increasing | Unadjusted | 1 | 4.869 | 4.097 | 4.878 | 3.812 |
| Monocyte count (10^9^/L) increasing | Adjusted | 0.1 | 1.208 | 1.182 | 1.177 | 1.158 |
| Monocyte count (10^9^/L) increasing | Adjusted | 0.5 | 2.574 | 2.312 | 2.263 | 2.084 |
| Monocyte count (10^9^/L) increasing | Adjusted | 1 | 6.628 | 5.345 | 5.120 | 4.341 |
| Neutrophil count (10^9^/L) increasing | Unadjusted | 1 | 1.255 | 1.233 | 1.205 | 1.198 |
| Neutrophil count (10^9^/L) increasing | Unadjusted | 5 | 3.113 | 2.850 | 2.541 | 2.468 |
| Neutrophil count (10^9^/L) increasing | Unadjusted | 10 | 9.693 | 8.121 | 6.455 | 6.089 |
| Neutrophil count (10^9^/L) increasing | Adjusted | 1 | 1.251 | 1.226 | 1.217 | 1.230 |
| Neutrophil count (10^9^/L) increasing | Adjusted | 5 | 3.064 | 2.770 | 2.670 | 2.815 |
| Neutrophil count (10^9^/L) increasing | Adjusted | 10 | 9.388 | 7.672 | 7.127 | 7.926 |
| CRP (mg/L) increasing | Unadjusted | 1 | 1.021 | 1.023 | 1.023 | 1.024 |
| ESR (mm/h) increasing | Unadjusted | 5 | 1.110 | 1.120 | 1.120 | 1.126 |
| ESR (mm/h) increasing | Unadjusted | 10 | 1.231 | 1.255 | 1.255 | 1.268 |
| ESR (mm/h) increasing | Adjusted | 1 | 1.021 | 1.023 | 1.024 | 1.024 |
| ESR (mm/h) increasing | Adjusted | 5 | 1.110 | 1.120 | 1.126 | 1.126 |
| ESR (mm/h) increasing | Adjusted | 10 | 1.231 | 1.255 | 1.268 | 1.268 |
| ESR (mm/h) increasing | Unadjusted | 1 | 1.021 | 1.023 | 1.023 | 1.023 |
| ESR (mm/h) increasing | Unadjusted | 5 | 1.110 | 1.120 | 1.120 | 1.120 |
| ESR (mm/h) increasing | Unadjusted | 10 | 1.231 | 1.255 | 1.255 | 1.255 |
| ESR (mm/h) increasing | Adjusted | 1 | 1.021 | 1.023 | 1.024 | 1.024 |
| ESR (mm/h) increasing | Adjusted | 5 | 1.110 | 1.120 | 1.126 | 1.126 |
| ESR (mm/h) increasing | Adjusted | 10 | 1.231 | 1.255 | 1.268 | 1.268 |
| Plasma viscosity (mPa.S) increasing | Unadjusted | 0.1 | 1.267 | 1.077 | 1.076 | 1.076 |
| Plasma viscosity (mPa.S) increasing | Unadjusted | 0.5 | 3.263 | 1.446 | 1.444 | 1.443 |
| Plasma viscosity (mPa.S) increasing | Unadjusted | 1 | 10.649 | 2.092 | 2.085 | 2.083 |
| Plasma viscosity (mPa.S) increasing | Adjusted | 0.1 | 1.259 | 1.256 | 1.315 | 1.381 |
| Plasma viscosity (mPa.S) increasing | Adjusted | 0.5 | 3.161 | 3.123 | 3.928 | 5.026 |
| Plasma viscosity (mPa.S) increasing | Adjusted | 1 | 9.992 | 9.754 | 15.429 | 25.259 |
| NLR increasing | Unadjusted | 1 | 1.179 | 1.141 | 1.144 | 1.100 |
| NLR increasing | Unadjusted | 5 | 2.278 | 1.934 | 1.959 | 1.611 |
| NLR increasing | Unadjusted | 10 | 5.190 | 3.740 | 3.839 | 2.594 |
| NLR increasing | Adjusted | 1 | 1.226 | 1.152 | 1.122 | 1.134 |
| NLR increasing | Adjusted | 5 | 2.770 | 2.029 | 1.778 | 1.875 |
| NLR increasing | Adjusted | 10 | 7.672 | 4.116 | 3.162 | 3.517 |
| PLR increasing | Unadjusted | 1 | 1.003 | 1.002 | 1.002 | 1.001 |
| PLR increasing | Unadjusted | 5 | 1.015 | 1.010 | 1.010 | 1.005 |
| PLR increasing | Unadjusted | 10 | 1.030 | 1.020 | 1.020 | 1.010 |
| PLR increasing | Adjusted | 1 | 1.003 | 1.003 | 1.002 | 1.002 |
| PLR increasing | Adjusted | 5 | 1.015 | 1.015 | 1.010 | 1.010 |
| PLR increasing | Adjusted | 10 | 1.030 | 1.030 | 1.020 | 1.020 |
| mGPR increasing | Unadjusted | 0.1 | 1.068 | 1.080 | 1.079 | 1.080 |
| mGPR increasing | Unadjusted | 0.5 | 1.387 | 1.467 | 1.460 | 1.471 |
| mGPR increasing | Unadjusted | 1 | 1.923 | 2.153 | 2.131 | 2.165 |
| mGPR increasing | Adjusted | 0.1 | 1.071 | 1.078 | 1.082 | 1.084 |
| mGPR increasing | Adjusted | 0.5 | 1.411 | 1.457 | 1.484 | 1.497 |
| mGPR increasing | Adjusted | 1 | 1.991 | 2.124 | 2.202 | 2.240 |
| These HRs have been rescaled to present how a 5- and 10-unit change in the blood test trend per year is associated with the hazard rate of cancer within 6 months following UWL, except 0.1- and 0.5-unit changes were used for basophils, monocytes, plasma viscosity, and mGPR, as these test result values are on the decimal scale.  Abbreviations: UWL=unexpected weight loss: ALT=alanine aminotransferase; AST=aspartate aminotransferase; RBC=red blood cell count; MCH=mean cell haemoglobin; MCHC=mean cell haemoglobin concentration; MCV=mean cell volume; RDW=red cell distribution width; MPV=mean platelet volume; WBC=white blood cell count; CRP=C-reactive protein; ESR=erythrocyte sedimentation rate; NLR=neutrophil-lymphocyte ratio; PLR=platelet-lymphocyte ratio; mGPR=modified Glasgow Prognostic ratio. | | | | | | |

# Table F: AUC (95% CI) of 1-year blood test trend and abnormality for 6-month cancer risk following UWL by cancer site

| Blood test | Site | Unadjusted | | Adjusted | |  |
| --- | --- | --- | --- | --- | --- | --- |
|  |  | **Trend** | **Abnormality** | **Trend** | **Abnormality** | |
| Albumin (g/L) declining | Bladder | 0.60 (0.55-0.65) | 0.54 (0.49-0.58) | 0.69 (0.64-0.73) | 0.68 (0.64-0.73) | |
| Albumin (g/L) declining | Bowel | 0.62 (0.59-0.64) | 0.57 (0.55-0.59) | 0.67 (0.65-0.69) | 0.65 (0.63-0.67) | |
| Albumin (g/L) declining | Brain | 0.45 (0.31-0.58) | 0.47 (0.41-0.53) | 0.40 (0.29-0.51) | 0.39 (0.28-0.49) | |
| Albumin (g/L) declining | Breast | 0.53 (0.47-0.58) | 0.54 (0.49-0.58) | 0.47 (0.43-0.52) | 0.48 (0.43-0.53) | |
| Albumin (g/L) declining | HandN | 0.51 (0.42-0.61) | 0.53 (0.46-0.60) | 0.65 (0.57-0.73) | 0.66 (0.58-0.74) | |
| Albumin (g/L) declining | Kidney | 0.67 (0.62-0.73) | 0.61 (0.56-0.66) | 0.67 (0.62-0.71) | 0.65 (0.60-0.70) | |
| Albumin (g/L) declining | Leukaemia | 0.48 (0.40-0.56) | 0.51 (0.46-0.57) | 0.64 (0.57-0.70) | 0.64 (0.58-0.69) | |
| Albumin (g/L) declining | Liver | 0.64 (0.58-0.69) | 0.59 (0.55-0.64) | 0.72 (0.69-0.76) | 0.71 (0.67-0.74) | |
| Albumin (g/L) declining | Lung | 0.61 (0.59-0.63) | 0.57 (0.55-0.59) | 0.67 (0.65-0.69) | 0.66 (0.64-0.68) | |
| Albumin (g/L) declining | Lymphoma | 0.63 (0.59-0.67) | 0.57 (0.54-0.61) | 0.63 (0.60-0.67) | 0.61 (0.57-0.64) | |
| Albumin (g/L) declining | Melanoma | 0.44 (0.34-0.55) | 0.52 (0.43-0.60) | 0.67 (0.56-0.78) | 0.71 (0.61-0.81) | |
| Albumin (g/L) declining | Myeloma | 0.64 (0.56-0.71) | 0.55 (0.49-0.61) | 0.63 (0.57-0.68) | 0.60 (0.54-0.67) | |
| Albumin (g/L) declining | Oesophagus | 0.54 (0.49-0.58) | 0.52 (0.49-0.55) | 0.68 (0.65-0.71) | 0.67 (0.64-0.70) | |
| Albumin (g/L) declining | Ovarian | 0.60 (0.51-0.68) | 0.62 (0.55-0.69) | 0.50 (0.44-0.55) | 0.52 (0.46-0.59) | |
| Albumin (g/L) declining | Pancreas | 0.49 (0.46-0.52) | 0.51 (0.50-0.53) | 0.60 (0.57-0.62) | 0.61 (0.58-0.63) | |
| Albumin (g/L) declining | Prostate | 0.52 (0.48-0.55) | 0.53 (0.51-0.56) | 0.80 (0.78-0.82) | 0.81 (0.79-0.83) | |
| Albumin (g/L) declining | Stomach | 0.54 (0.50-0.58) | 0.53 (0.50-0.56) | 0.69 (0.66-0.72) | 0.69 (0.65-0.72) | |
| Albumin (g/L) declining | Thyroid | 0.33 (0.17-0.49) | 0.51 (0.37-0.65) | 0.54 (0.40-0.69) | 0.59 (0.44-0.75) | |
| Albumin (g/L) declining | Uterine | 0.58 (0.46-0.71) | 0.56 (0.47-0.65) | 0.44 (0.35-0.54) | 0.41 (0.32-0.51) | |
| Alkaline phosphatase (iu/L) increasing | Bladder | 0.52 (0.46-0.59) | 0.48 (0.44-0.52) | 0.68 (0.63-0.72) | 0.66 (0.62-0.71) | |
| Alkaline phosphatase (iu/L) increasing | Bowel | 0.59 (0.56-0.62) | 0.55 (0.52-0.57) | 0.65 (0.63-0.67) | 0.64 (0.62-0.66) | |
| Alkaline phosphatase (iu/L) increasing | Brain | 0.41 (0.30-0.51) | 0.45 (0.40-0.51) | 0.37 (0.27-0.47) | 0.38 (0.28-0.47) | |
| Alkaline phosphatase (iu/L) increasing | Breast | 0.54 (0.48-0.61) | 0.56 (0.50-0.61) | 0.48 (0.43-0.52) | 0.51 (0.46-0.56) | |
| Alkaline phosphatase (iu/L) increasing | HandN | 0.54 (0.45-0.63) | 0.52 (0.44-0.59) | 0.62 (0.54-0.69) | 0.60 (0.52-0.67) | |
| Alkaline phosphatase (iu/L) increasing | Kidney | 0.62 (0.57-0.67) | 0.57 (0.53-0.62) | 0.63 (0.59-0.67) | 0.61 (0.57-0.66) | |
| Alkaline phosphatase (iu/L) increasing | Leukaemia | 0.55 (0.47-0.64) | 0.55 (0.48-0.62) | 0.66 (0.60-0.73) | 0.65 (0.59-0.71) | |
| Alkaline phosphatase (iu/L) increasing | Liver | 0.80 (0.75-0.85) | 0.75 (0.68-0.81) | 0.80 (0.76-0.84) | 0.78 (0.74-0.82) | |
| Alkaline phosphatase (iu/L) increasing | Lung | 0.63 (0.61-0.65) | 0.56 (0.54-0.58) | 0.67 (0.66-0.69) | 0.66 (0.64-0.68) | |
| Alkaline phosphatase (iu/L) increasing | Lymphoma | 0.60 (0.56-0.64) | 0.56 (0.52-0.59) | 0.62 (0.58-0.65) | 0.61 (0.57-0.64) | |
| Alkaline phosphatase (iu/L) increasing | Melanoma | 0.49 (0.35-0.62) | 0.51 (0.42-0.59) | 0.65 (0.53-0.76) | 0.67 (0.56-0.78) | |
| Alkaline phosphatase (iu/L) increasing | Myeloma | 0.53 (0.44-0.61) | 0.52 (0.45-0.59) | 0.53 (0.47-0.59) | 0.54 (0.48-0.60) | |
| Alkaline phosphatase (iu/L) increasing | Oesophagus | 0.53 (0.49-0.58) | 0.51 (0.48-0.54) | 0.67 (0.64-0.70) | 0.66 (0.63-0.69) | |
| Alkaline phosphatase (iu/L) increasing | Ovarian | 0.57 (0.48-0.66) | 0.51 (0.45-0.58) | 0.44 (0.38-0.49) | 0.44 (0.39-0.50) | |
| Alkaline phosphatase (iu/L) increasing | Pancreas | 0.59 (0.56-0.62) | 0.59 (0.56-0.61) | 0.66 (0.64-0.68) | 0.65 (0.62-0.67) | |
| Alkaline phosphatase (iu/L) increasing | Prostate | 0.57 (0.53-0.61) | 0.59 (0.56-0.63) | 0.82 (0.80-0.84) | 0.81 (0.79-0.83) | |
| Alkaline phosphatase (iu/L) increasing | Stomach | 0.51 (0.47-0.55) | 0.52 (0.49-0.55) | 0.67 (0.64-0.71) | 0.68 (0.65-0.71) | |
| Alkaline phosphatase (iu/L) increasing | Thyroid | 0.61 (0.42-0.80) | 0.64 (0.44-0.84) | 0.65 (0.51-0.79) | 0.63 (0.49-0.77) | |
| Alkaline phosphatase (iu/L) increasing | Uterine | 0.56 (0.43-0.69) | 0.55 (0.44-0.66) | 0.45 (0.36-0.54) | 0.46 (0.36-0.55) | |
| ALT (iu/L) declining | Bladder | 0.60 (0.54-0.66) | 0.48 (0.44-0.51) | 0.68 (0.63-0.73) | 0.67 (0.62-0.72) | |
| ALT (iu/L) declining | Bowel | 0.54 (0.52-0.57) | 0.50 (0.48-0.51) | 0.62 (0.59-0.64) | 0.62 (0.60-0.64) | |
| ALT (iu/L) declining | Brain | 0.51 (0.36-0.66) | 0.51 (0.42-0.60) | 0.37 (0.27-0.46) | 0.37 (0.28-0.47) | |
| ALT (iu/L) declining | Breast | 0.46 (0.41-0.52) | 0.46 (0.44-0.49) | 0.46 (0.42-0.50) | 0.48 (0.43-0.52) | |
| ALT (iu/L) declining | HandN | 0.58 (0.48-0.67) | 0.48 (0.43-0.52) | 0.64 (0.54-0.73) | 0.62 (0.52-0.72) | |
| ALT (iu/L) declining | Kidney | 0.56 (0.50-0.62) | 0.51 (0.47-0.54) | 0.56 (0.52-0.61) | 0.55 (0.50-0.60) | |
| ALT (iu/L) declining | Leukaemia | 0.47 (0.39-0.54) | 0.53 (0.47-0.59) | 0.64 (0.58-0.70) | 0.64 (0.58-0.70) | |
| ALT (iu/L) declining | Liver | 0.25 (0.20-0.30) | 0.66 (0.61-0.72) | 0.63 (0.59-0.67) | 0.69 (0.64-0.73) | |
| ALT (iu/L) declining | Lung | 0.57 (0.55-0.60) | 0.49 (0.48-0.50) | 0.64 (0.62-0.66) | 0.63 (0.61-0.65) | |
| ALT (iu/L) declining | Lymphoma | 0.50 (0.46-0.54) | 0.51 (0.48-0.54) | 0.60 (0.56-0.63) | 0.59 (0.56-0.63) | |
| ALT (iu/L) declining | Melanoma | 0.37 (0.23-0.51) | 0.57 (0.44-0.70) | 0.69 (0.58-0.79) | 0.71 (0.60-0.82) | |
| ALT (iu/L) declining | Myeloma | 0.56 (0.48-0.64) | 0.48 (0.44-0.52) | 0.57 (0.51-0.63) | 0.56 (0.50-0.61) | |
| ALT (iu/L) declining | Oesophagus | 0.56 (0.51-0.61) | 0.50 (0.47-0.53) | 0.67 (0.64-0.71) | 0.66 (0.62-0.69) | |
| ALT (iu/L) declining | Ovarian | 0.49 (0.42-0.57) | 0.48 (0.44-0.52) | 0.43 (0.38-0.48) | 0.45 (0.39-0.50) | |
| ALT (iu/L) declining | Pancreas | 0.45 (0.42-0.48) | 0.54 (0.52-0.57) | 0.61 (0.59-0.64) | 0.62 (0.60-0.65) | |
| ALT (iu/L) declining | Prostate | 0.56 (0.52-0.59) | 0.48 (0.47-0.50) | 0.83 (0.82-0.85) | 0.82 (0.80-0.83) | |
| ALT (iu/L) declining | Stomach | 0.52 (0.48-0.56) | 0.50 (0.47-0.52) | 0.68 (0.64-0.71) | 0.67 (0.63-0.70) | |
| ALT (iu/L) declining | Thyroid | 0.59 (0.42-0.76) | 0.44 (0.44-0.45) | 0.63 (0.47-0.78) | 0.61 (0.47-0.76) | |
| ALT (iu/L) declining | Uterine | 0.44 (0.33-0.55) | 0.44 (0.44-0.45) | 0.35 (0.27-0.44) | 0.36 (0.27-0.44) | |
| AST (iu/L) declining | Bladder | 0.65 (0.52-0.78) | 0.48 (0.41-0.56) | 0.68 (0.55-0.81) | 0.65 (0.52-0.79) | |
| AST (iu/L) declining | Bowel | 0.55 (0.49-0.61) | 0.51 (0.48-0.55) | 0.64 (0.59-0.69) | 0.64 (0.60-0.69) | |
| AST (iu/L) declining | Brain | 0.34 (0.08-0.60) | 0.44 (0.44-0.45) | 0.39 (0.27-0.51) | 0.42 (0.29-0.56) | |
| AST (iu/L) declining | Breast | 0.41 (0.25-0.56) | 0.59 (0.44-0.74) | 0.38 (0.22-0.55) | 0.48 (0.27-0.68) | |
| AST (iu/L) declining | HandN | 0.61 (0.43-0.79) | 0.53 (0.36-0.69) | 0.47 (0.33-0.60) | 0.52 (0.38-0.67) | |
| AST (iu/L) declining | Kidney | 0.61 (0.51-0.72) | 0.47 (0.42-0.51) | 0.65 (0.55-0.75) | 0.61 (0.51-0.71) | |
| AST (iu/L) declining | Leukaemia | 0.46 (0.30-0.62) | 0.61 (0.40-0.82) | 0.65 (0.38-0.92) | 0.66 (0.44-0.89) | |
| AST (iu/L) declining | Liver | 0.27 (0.15-0.39) | 0.82 (0.72-0.92) | 0.64 (0.55-0.72) | 0.74 (0.64-0.84) | |
| AST (iu/L) declining | Lung | 0.59 (0.54-0.63) | 0.50 (0.48-0.53) | 0.67 (0.63-0.72) | 0.66 (0.62-0.71) | |
| AST (iu/L) declining | Lymphoma | 0.45 (0.36-0.53) | 0.54 (0.48-0.61) | 0.48 (0.40-0.56) | 0.48 (0.40-0.57) | |
| AST (iu/L) declining | Melanoma | 0.45 (0.03-0.87) | 0.44 (0.44-0.45) | 0.83 (0.60-1.00) | 0.86 (0.71-1.00) | |
| AST (iu/L) declining | Myeloma | 0.43 (0.17-0.69) | 0.57 (0.32-0.81) | 0.49 (0.38-0.59) | 0.54 (0.30-0.78) | |
| AST (iu/L) declining | Oesophagus | 0.50 (0.44-0.57) | 0.46 (0.43-0.48) | 0.69 (0.63-0.76) | 0.68 (0.61-0.74) | |
| AST (iu/L) declining | Ovarian | 0.36 (0.21-0.51) | 0.63 (0.45-0.81) | 0.37 (0.23-0.50) | 0.49 (0.35-0.64) | |
| AST (iu/L) declining | Pancreas | 0.51 (0.44-0.59) | 0.52 (0.48-0.57) | 0.59 (0.53-0.66) | 0.59 (0.52-0.66) | |
| AST (iu/L) declining | Prostate | 0.52 (0.45-0.59) | 0.47 (0.44-0.50) | 0.81 (0.79-0.83) | 0.76 (0.73-0.79) | |
| AST (iu/L) declining | Stomach | 0.50 (0.43-0.57) | 0.52 (0.47-0.57) | 0.68 (0.62-0.73) | 0.67 (0.62-0.73) | |
| AST (iu/L) declining | Thyroid | 0.16 (0.00-0.36) | 0.94 | 0.17 | 0.37 | |
| AST (iu/L) declining | Uterine | 0.51 (0.23-0.80) | 0.69 (0.20-1.00) | 0.26 (0.00-0.55) | 0.43 (0.00-1.00) | |
| Bilirubin (umol/L) declining | Bladder | 0.56 (0.50-0.62) | 0.52 (0.49-0.54) | 0.68 (0.64-0.73) | 0.67 (0.63-0.72) | |
| Bilirubin (umol/L) declining | Bowel | 0.57 (0.55-0.60) | 0.52 (0.50-0.53) | 0.63 (0.61-0.65) | 0.62 (0.60-0.64) | |
| Bilirubin (umol/L) declining | Brain | 0.48 (0.34-0.63) | 0.54 (0.54-0.55) | 0.37 (0.28-0.46) | 0.39 (0.30-0.47) | |
| Bilirubin (umol/L) declining | Breast | 0.49 (0.44-0.55) | 0.52 (0.50-0.55) | 0.46 (0.42-0.50) | 0.48 (0.44-0.52) | |
| Bilirubin (umol/L) declining | HandN | 0.60 (0.52-0.69) | 0.54 (0.54-0.55) | 0.64 (0.55-0.72) | 0.63 (0.55-0.72) | |
| Bilirubin (umol/L) declining | Kidney | 0.57 (0.52-0.62) | 0.51 (0.49-0.54) | 0.58 (0.54-0.63) | 0.57 (0.53-0.62) | |
| Bilirubin (umol/L) declining | Leukaemia | 0.53 (0.46-0.60) | 0.49 (0.45-0.54) | 0.65 (0.59-0.71) | 0.65 (0.59-0.71) | |
| Bilirubin (umol/L) declining | Liver | 0.38 (0.32-0.43) | 0.43 (0.39-0.47) | 0.63 (0.59-0.67) | 0.64 (0.60-0.68) | |
| Bilirubin (umol/L) declining | Lung | 0.58 (0.56-0.60) | 0.52 (0.52-0.53) | 0.64 (0.63-0.66) | 0.64 (0.62-0.65) | |
| Bilirubin (umol/L) declining | Lymphoma | 0.48 (0.43-0.52) | 0.47 (0.45-0.50) | 0.57 (0.54-0.61) | 0.57 (0.53-0.60) | |
| Bilirubin (umol/L) declining | Melanoma | 0.47 (0.32-0.62) | 0.48 (0.39-0.57) | 0.69 (0.59-0.80) | 0.71 (0.61-0.81) | |
| Bilirubin (umol/L) declining | Myeloma | 0.54 (0.46-0.62) | 0.51 (0.46-0.55) | 0.57 (0.51-0.62) | 0.56 (0.51-0.61) | |
| Bilirubin (umol/L) declining | Oesophagus | 0.47 (0.43-0.52) | 0.48 (0.45-0.51) | 0.66 (0.63-0.70) | 0.67 (0.63-0.70) | |
| Bilirubin (umol/L) declining | Ovarian | 0.50 (0.42-0.57) | 0.53 (0.51-0.56) | 0.41 (0.37-0.46) | 0.43 (0.38-0.48) | |
| Bilirubin (umol/L) declining | Pancreas | 0.50 (0.47-0.53) | 0.48 (0.47-0.50) | 0.61 (0.59-0.63) | 0.61 (0.59-0.63) | |
| Bilirubin (umol/L) declining | Prostate | 0.53 (0.49-0.57) | 0.49 (0.47-0.51) | 0.83 (0.82-0.85) | 0.83 (0.81-0.84) | |
| Bilirubin (umol/L) declining | Stomach | 0.54 (0.50-0.58) | 0.50 (0.48-0.52) | 0.68 (0.65-0.71) | 0.68 (0.65-0.71) | |
| Bilirubin (umol/L) declining | Thyroid | 0.60 (0.46-0.75) | 0.49 (0.40-0.59) | 0.64 (0.46-0.82) | 0.63 (0.47-0.80) | |
| Bilirubin (umol/L) declining | Uterine | 0.58 (0.44-0.73) | 0.49 (0.41-0.56) | 0.36 (0.28-0.43) | 0.36 (0.28-0.44) | |
| RBC (10^12^/L) declining | Bladder | 0.45 (0.39-0.51) | 0.55 (0.50-0.61) | 0.70 (0.65-0.74) | 0.70 (0.66-0.74) | |
| RBC (10^12^/L) declining | Bowel | 0.52 (0.50-0.55) | 0.58 (0.56-0.61) | 0.65 (0.63-0.66) | 0.65 (0.63-0.67) | |
| RBC (10^12^/L) declining | Brain | 0.48 (0.32-0.64) | 0.47 (0.34-0.59) | 0.42 (0.31-0.53) | 0.42 (0.31-0.53) | |
| RBC (10^12^/L) declining | Breast | 0.50 (0.44-0.57) | 0.47 (0.42-0.53) | 0.45 (0.41-0.49) | 0.48 (0.44-0.51) | |
| RBC (10^12^/L) declining | HandN | 0.58 (0.49-0.67) | 0.71 (0.64-0.77) | 0.63 (0.55-0.71) | 0.64 (0.56-0.72) | |
| RBC (10^12^/L) declining | Kidney | 0.52 (0.47-0.58) | 0.59 (0.54-0.64) | 0.59 (0.55-0.63) | 0.60 (0.56-0.64) | |
| RBC (10^12^/L) declining | Leukaemia | 0.67 (0.59-0.74) | 0.69 (0.63-0.75) | 0.66 (0.60-0.71) | 0.68 (0.63-0.73) | |
| RBC (10^12^/L) declining | Liver | 0.49 (0.43-0.55) | 0.58 (0.53-0.63) | 0.67 (0.63-0.71) | 0.66 (0.63-0.70) | |
| RBC (10^12^/L) declining | Lung | 0.48 (0.46-0.50) | 0.56 (0.54-0.58) | 0.64 (0.62-0.66) | 0.64 (0.62-0.66) | |
| RBC (10^12^/L) declining | Lymphoma | 0.50 (0.46-0.54) | 0.54 (0.50-0.58) | 0.57 (0.54-0.60) | 0.57 (0.54-0.60) | |
| RBC (10^12^/L) declining | Melanoma | 0.47 (0.34-0.59) | 0.55 (0.41-0.69) | 0.71 (0.60-0.82) | 0.70 (0.59-0.81) | |
| RBC (10^12^/L) declining | Myeloma | 0.73 (0.68-0.79) | 0.70 (0.64-0.75) | 0.60 (0.55-0.65) | 0.63 (0.58-0.68) | |
| RBC (10^12^/L) declining | Oesophagus | 0.46 (0.42-0.51) | 0.51 (0.47-0.55) | 0.68 (0.65-0.71) | 0.67 (0.64-0.70) | |
| RBC (10^12^/L) declining | Ovarian | 0.53 (0.46-0.60) | 0.49 (0.42-0.57) | 0.43 (0.39-0.47) | 0.45 (0.41-0.49) | |
| RBC (10^12^/L) declining | Pancreas | 0.44 (0.41-0.47) | 0.50 (0.47-0.53) | 0.62 (0.60-0.64) | 0.61 (0.59-0.63) | |
| RBC (10^12^/L) declining | Prostate | 0.46 (0.42-0.50) | 0.63 (0.60-0.66) | 0.82 (0.81-0.83) | 0.80 (0.79-0.81) | |
| RBC (10^12^/L) declining | Stomach | 0.50 (0.46-0.55) | 0.58 (0.54-0.62) | 0.69 (0.66-0.72) | 0.69 (0.66-0.72) | |
| RBC (10^12^/L) declining | Thyroid | 0.45 (0.26-0.64) | 0.52 (0.35-0.69) | 0.55 (0.43-0.68) | 0.56 (0.43-0.69) | |
| RBC (10^12^/L) declining | Uterine | 0.49 (0.38-0.60) | 0.42 (0.32-0.53) | 0.35 (0.28-0.42) | 0.37 (0.29-0.44) | |
| Haemoglobin (g/L) declining | Bladder | 0.54 (0.47-0.61) | 0.59 (0.52-0.65) | 0.70 (0.65-0.74) | 0.70 (0.67-0.74) | |
| Haemoglobin (g/L) declining | Bowel | 0.66 (0.64-0.69) | 0.65 (0.63-0.68) | 0.71 (0.69-0.72) | 0.70 (0.68-0.72) | |
| Haemoglobin (g/L) declining | Brain | 0.37 (0.25-0.50) | 0.39 (0.31-0.47) | 0.39 (0.29-0.49) | 0.40 (0.29-0.50) | |
| Haemoglobin (g/L) declining | Breast | 0.51 (0.44-0.58) | 0.51 (0.44-0.57) | 0.48 (0.44-0.53) | 0.48 (0.44-0.52) | |
| Haemoglobin (g/L) declining | HandN | 0.55 (0.45-0.65) | 0.61 (0.52-0.70) | 0.65 (0.58-0.73) | 0.65 (0.58-0.72) | |
| Haemoglobin (g/L) declining | Kidney | 0.70 (0.65-0.75) | 0.69 (0.64-0.73) | 0.68 (0.64-0.71) | 0.67 (0.63-0.70) | |
| Haemoglobin (g/L) declining | Leukaemia | 0.58 (0.49-0.66) | 0.62 (0.54-0.70) | 0.69 (0.63-0.75) | 0.69 (0.63-0.74) | |
| Haemoglobin (g/L) declining | Liver | 0.51 (0.45-0.57) | 0.57 (0.52-0.62) | 0.66 (0.63-0.70) | 0.67 (0.64-0.70) | |
| Haemoglobin (g/L) declining | Lung | 0.55 (0.53-0.57) | 0.59 (0.57-0.61) | 0.65 (0.63-0.67) | 0.66 (0.64-0.67) | |
| Haemoglobin (g/L) declining | Lymphoma | 0.62 (0.57-0.66) | 0.62 (0.58-0.66) | 0.64 (0.61-0.67) | 0.64 (0.61-0.67) | |
| Haemoglobin (g/L) declining | Melanoma | 0.49 (0.37-0.61) | 0.52 (0.40-0.64) | 0.68 (0.58-0.78) | 0.69 (0.59-0.79) | |
| Haemoglobin (g/L) declining | Myeloma | 0.71 (0.63-0.78) | 0.64 (0.57-0.71) | 0.70 (0.65-0.74) | 0.67 (0.62-0.72) | |
| Haemoglobin (g/L) declining | Oesophagus | 0.47 (0.42-0.51) | 0.51 (0.48-0.55) | 0.65 (0.62-0.68) | 0.66 (0.63-0.69) | |
| Haemoglobin (g/L) declining | Ovarian | 0.59 (0.52-0.65) | 0.50 (0.44-0.57) | 0.49 (0.45-0.53) | 0.48 (0.44-0.53) | |
| Haemoglobin (g/L) declining | Pancreas | 0.48 (0.45-0.51) | 0.52 (0.50-0.55) | 0.60 (0.58-0.62) | 0.61 (0.59-0.63) | |
| Haemoglobin (g/L) declining | Prostate | 0.53 (0.49-0.57) | 0.64 (0.60-0.67) | 0.81 (0.79-0.83) | 0.82 (0.80-0.84) | |
| Haemoglobin (g/L) declining | Stomach | 0.57 (0.53-0.62) | 0.64 (0.60-0.68) | 0.71 (0.68-0.73) | 0.71 (0.68-0.73) | |
| Haemoglobin (g/L) declining | Thyroid | 0.40 (0.31-0.50) | 0.40 (0.30-0.50) | 0.53 (0.40-0.67) | 0.55 (0.41-0.68) | |
| Haemoglobin (g/L) declining | Uterine | 0.53 (0.38-0.67) | 0.50 (0.35-0.65) | 0.43 (0.37-0.49) | 0.41 (0.35-0.48) | |
| Haematocrit (L/L) declining | Bladder | 0.52 (0.46-0.59) | 0.58 (0.52-0.64) | 0.70 (0.66-0.74) | 0.71 (0.67-0.75) | |
| Haematocrit (L/L) declining | Bowel | 0.46 (0.43-0.48) | 0.63 (0.60-0.65) | 0.65 (0.63-0.66) | 0.69 (0.67-0.71) | |
| Haematocrit (L/L) declining | Brain | 0.48 (0.32-0.64) | 0.43 (0.36-0.50) | 0.42 (0.31-0.52) | 0.41 (0.29-0.52) | |
| Haematocrit (L/L) declining | Breast | 0.32 (0.27-0.36) | 0.52 (0.47-0.57) | 0.46 (0.42-0.50) | 0.48 (0.44-0.53) | |
| Haematocrit (L/L) declining | HandN | 0.57 (0.48-0.66) | 0.54 (0.46-0.62) | 0.62 (0.55-0.70) | 0.63 (0.56-0.71) | |
| Haematocrit (L/L) declining | Kidney | 0.48 (0.43-0.54) | 0.65 (0.60-0.70) | 0.58 (0.54-0.62) | 0.65 (0.61-0.69) | |
| Haematocrit (L/L) declining | Leukaemia | 0.49 (0.41-0.56) | 0.67 (0.60-0.74) | 0.65 (0.60-0.71) | 0.69 (0.64-0.75) | |
| Haematocrit (L/L) declining | Liver | 0.58 (0.52-0.64) | 0.52 (0.47-0.57) | 0.67 (0.63-0.71) | 0.67 (0.63-0.70) | |
| Haematocrit (L/L) declining | Lung | 0.52 (0.50-0.54) | 0.54 (0.52-0.56) | 0.64 (0.62-0.66) | 0.65 (0.63-0.66) | |
| Haematocrit (L/L) declining | Lymphoma | 0.51 (0.47-0.55) | 0.59 (0.56-0.63) | 0.57 (0.54-0.60) | 0.61 (0.58-0.64) | |
| Haematocrit (L/L) declining | Melanoma | 0.62 (0.47-0.77) | 0.47 (0.36-0.57) | 0.73 (0.63-0.84) | 0.69 (0.59-0.79) | |
| Haematocrit (L/L) declining | Myeloma | 0.41 (0.33-0.49) | 0.66 (0.59-0.73) | 0.58 (0.53-0.63) | 0.66 (0.61-0.71) | |
| Haematocrit (L/L) declining | Oesophagus | 0.58 (0.54-0.63) | 0.49 (0.46-0.53) | 0.67 (0.64-0.70) | 0.66 (0.63-0.69) | |
| Haematocrit (L/L) declining | Ovarian | 0.26 (0.19-0.33) | 0.58 (0.51-0.65) | 0.44 (0.40-0.48) | 0.47 (0.43-0.52) | |
| Haematocrit (L/L) declining | Pancreas | 0.51 (0.48-0.54) | 0.49 (0.47-0.52) | 0.62 (0.60-0.65) | 0.61 (0.59-0.63) | |
| Haematocrit (L/L) declining | Prostate | 0.69 (0.66-0.71) | 0.55 (0.52-0.58) | 0.81 (0.80-0.82) | 0.78 (0.77-0.79) | |
| Haematocrit (L/L) declining | Stomach | 0.55 (0.51-0.59) | 0.58 (0.55-0.62) | 0.69 (0.66-0.72) | 0.70 (0.67-0.73) | |
| Haematocrit (L/L) declining | Thyroid | 0.44 (0.30-0.57) | 0.51 (0.39-0.62) | 0.57 (0.45-0.69) | 0.57 (0.43-0.70) | |
| Haematocrit (L/L) declining | Uterine | 0.31 (0.21-0.41) | 0.48 (0.38-0.57) | 0.36 (0.29-0.43) | 0.39 (0.32-0.46) | |
| MCH (pg) declining | Bladder | 0.54 (0.47-0.60) | 0.52 (0.48-0.57) | 0.69 (0.64-0.73) | 0.69 (0.65-0.73) | |
| MCH (pg) declining | Bowel | 0.67 (0.65-0.70) | 0.61 (0.58-0.63) | 0.73 (0.72-0.75) | 0.71 (0.69-0.73) | |
| MCH (pg) declining | Brain | 0.49 (0.35-0.62) | 0.51 (0.43-0.59) | 0.41 (0.31-0.50) | 0.41 (0.31-0.52) | |
| MCH (pg) declining | Breast | 0.49 (0.42-0.55) | 0.54 (0.49-0.58) | 0.46 (0.42-0.51) | 0.48 (0.43-0.52) | |
| MCH (pg) declining | HandN | 0.44 (0.32-0.55) | 0.51 (0.44-0.58) | 0.58 (0.48-0.67) | 0.62 (0.53-0.70) | |
| MCH (pg) declining | Kidney | 0.69 (0.63-0.75) | 0.62 (0.57-0.67) | 0.70 (0.66-0.74) | 0.69 (0.65-0.73) | |
| MCH (pg) declining | Leukaemia | 0.49 (0.41-0.57) | 0.49 (0.44-0.53) | 0.61 (0.55-0.67) | 0.63 (0.57-0.69) | |
| MCH (pg) declining | Liver | 0.55 (0.49-0.62) | 0.54 (0.50-0.58) | 0.65 (0.61-0.69) | 0.67 (0.63-0.71) | |
| MCH (pg) declining | Lung | 0.56 (0.54-0.58) | 0.52 (0.50-0.53) | 0.66 (0.64-0.68) | 0.65 (0.63-0.67) | |
| MCH (pg) declining | Lymphoma | 0.70 (0.66-0.73) | 0.59 (0.56-0.63) | 0.66 (0.63-0.69) | 0.64 (0.61-0.67) | |
| MCH (pg) declining | Melanoma | 0.44 (0.34-0.54) | 0.48 (0.42-0.55) | 0.68 (0.57-0.78) | 0.72 (0.61-0.82) | |
| MCH (pg) declining | Myeloma | 0.32 (0.25-0.39) | 0.46 (0.44-0.49) | 0.50 (0.44-0.56) | 0.57 (0.52-0.62) | |
| MCH (pg) declining | Oesophagus | 0.52 (0.47-0.57) | 0.50 (0.47-0.53) | 0.67 (0.63-0.70) | 0.67 (0.64-0.70) | |
| MCH (pg) declining | Ovarian | 0.63 (0.55-0.71) | 0.50 (0.45-0.55) | 0.52 (0.46-0.57) | 0.46 (0.40-0.51) | |
| MCH (pg) declining | Pancreas | 0.55 (0.52-0.58) | 0.50 (0.48-0.51) | 0.63 (0.61-0.65) | 0.61 (0.59-0.64) | |
| MCH (pg) declining | Prostate | 0.58 (0.54-0.62) | 0.51 (0.49-0.54) | 0.81 (0.79-0.83) | 0.82 (0.80-0.84) | |
| MCH (pg) declining | Stomach | 0.57 (0.53-0.62) | 0.55 (0.51-0.58) | 0.73 (0.69-0.76) | 0.72 (0.69-0.75) | |
| MCH (pg) declining | Thyroid | 0.64 (0.48-0.80) | 0.62 (0.45-0.78) | 0.60 (0.46-0.75) | 0.60 (0.45-0.75) | |
| MCH (pg) declining | Uterine | 0.64 (0.52-0.77) | 0.61 (0.50-0.71) | 0.51 (0.41-0.61) | 0.49 (0.37-0.61) | |
| MCHC (g/L) declining | Bladder | 0.57 (0.51-0.63) | 0.49 (0.47-0.51) | 0.71 (0.66-0.75) | 0.71 (0.66-0.75) | |
| MCHC (g/L) declining | Bowel | 0.61 (0.58-0.63) | 0.53 (0.51-0.55) | 0.65 (0.63-0.67) | 0.66 (0.64-0.68) | |
| MCHC (g/L) declining | Brain | 0.43 (0.29-0.57) | 0.47 (0.47-0.47) | 0.40 (0.31-0.50) | 0.40 (0.31-0.49) | |
| MCHC (g/L) declining | Breast | 0.46 (0.40-0.53) | 0.52 (0.48-0.55) | 0.48 (0.44-0.53) | 0.51 (0.46-0.55) | |
| MCHC (g/L) declining | HandN | 0.50 (0.41-0.59) | 0.49 (0.45-0.52) | 0.60 (0.51-0.69) | 0.60 (0.51-0.69) | |
| MCHC (g/L) declining | Kidney | 0.66 (0.61-0.71) | 0.54 (0.50-0.57) | 0.58 (0.53-0.62) | 0.58 (0.53-0.63) | |
| MCHC (g/L) declining | Leukaemia | 0.51 (0.42-0.59) | 0.50 (0.46-0.53) | 0.66 (0.60-0.72) | 0.66 (0.60-0.72) | |
| MCHC (g/L) declining | Liver | 0.53 (0.47-0.59) | 0.49 (0.47-0.51) | 0.66 (0.62-0.71) | 0.65 (0.61-0.70) | |
| MCHC (g/L) declining | Lung | 0.54 (0.52-0.57) | 0.51 (0.50-0.53) | 0.65 (0.63-0.67) | 0.65 (0.63-0.67) | |
| MCHC (g/L) declining | Lymphoma | 0.55 (0.51-0.60) | 0.53 (0.50-0.55) | 0.56 (0.53-0.60) | 0.56 (0.53-0.60) | |
| MCHC (g/L) declining | Melanoma | 0.50 (0.33-0.68) | 0.58 (0.44-0.73) | 0.74 (0.61-0.87) | 0.75 (0.63-0.87) | |
| MCHC (g/L) declining | Myeloma | 0.51 (0.43-0.59) | 0.51 (0.47-0.54) | 0.55 (0.50-0.61) | 0.55 (0.49-0.61) | |
| MCHC (g/L) declining | Oesophagus | 0.50 (0.45-0.55) | 0.49 (0.47-0.50) | 0.67 (0.63-0.71) | 0.67 (0.63-0.70) | |
| MCHC (g/L) declining | Ovarian | 0.56 (0.47-0.64) | 0.50 (0.46-0.54) | 0.43 (0.37-0.48) | 0.43 (0.38-0.49) | |
| MCHC (g/L) declining | Pancreas | 0.47 (0.44-0.50) | 0.49 (0.48-0.50) | 0.62 (0.59-0.65) | 0.62 (0.59-0.64) | |
| MCHC (g/L) declining | Prostate | 0.54 (0.50-0.58) | 0.48 (0.47-0.50) | 0.85 (0.83-0.87) | 0.84 (0.82-0.86) | |
| MCHC (g/L) declining | Stomach | 0.58 (0.53-0.62) | 0.51 (0.49-0.53) | 0.69 (0.66-0.73) | 0.69 (0.66-0.72) | |
| MCHC (g/L) declining | Thyroid | 0.44 (0.29-0.58) | 0.51 (0.44-0.58) | 0.58 (0.44-0.72) | 0.58 (0.44-0.73) | |
| MCHC (g/L) declining | Uterine | 0.57 (0.46-0.68) | 0.50 (0.44-0.56) | 0.39 (0.31-0.46) | 0.40 (0.31-0.48) | |
| MCV (fL) declining | Bladder | 0.61 (0.55-0.68) | 0.51 (0.49-0.53) | 0.69 (0.65-0.74) | 0.70 (0.66-0.74) | |
| MCV (fL) declining | Bowel | 0.67 (0.64-0.70) | 0.53 (0.51-0.54) | 0.72 (0.70-0.74) | 0.67 (0.65-0.69) | |
| MCV (fL) declining | Brain | 0.54 (0.41-0.68) | 0.49 (0.49-0.49) | 0.43 (0.33-0.52) | 0.41 (0.31-0.52) | |
| MCV (fL) declining | Breast | 0.50 (0.44-0.56) | 0.52 (0.49-0.54) | 0.47 (0.42-0.51) | 0.47 (0.43-0.51) | |
| MCV (fL) declining | HandN | 0.47 (0.37-0.58) | 0.49 (0.49-0.49) | 0.59 (0.50-0.68) | 0.62 (0.53-0.70) | |
| MCV (fL) declining | Kidney | 0.66 (0.61-0.72) | 0.55 (0.52-0.57) | 0.68 (0.65-0.72) | 0.62 (0.58-0.66) | |
| MCV (fL) declining | Leukaemia | 0.47 (0.39-0.54) | 0.52 (0.48-0.55) | 0.61 (0.55-0.67) | 0.66 (0.61-0.72) | |
| MCV (fL) declining | Liver | 0.54 (0.48-0.60) | 0.50 (0.49-0.52) | 0.66 (0.63-0.70) | 0.67 (0.63-0.70) | |
| MCV (fL) declining | Lung | 0.56 (0.54-0.58) | 0.51 (0.50-0.51) | 0.66 (0.64-0.68) | 0.64 (0.63-0.66) | |
| MCV (fL) declining | Lymphoma | 0.64 (0.60-0.68) | 0.52 (0.50-0.54) | 0.66 (0.63-0.69) | 0.59 (0.56-0.62) | |
| MCV (fL) declining | Melanoma | 0.48 (0.36-0.59) | 0.49 (0.49-0.49) | 0.69 (0.59-0.79) | 0.73 (0.63-0.82) | |
| MCV (fL) declining | Myeloma | 0.31 (0.24-0.38) | 0.49 (0.49-0.49) | 0.49 (0.43-0.55) | 0.58 (0.53-0.63) | |
| MCV (fL) declining | Oesophagus | 0.56 (0.51-0.60) | 0.50 (0.49-0.52) | 0.68 (0.65-0.71) | 0.67 (0.64-0.70) | |
| MCV (fL) declining | Ovarian | 0.57 (0.50-0.65) | 0.49 (0.49-0.49) | 0.49 (0.44-0.53) | 0.45 (0.41-0.50) | |
| MCV (fL) declining | Pancreas | 0.55 (0.53-0.58) | 0.50 (0.49-0.50) | 0.64 (0.62-0.66) | 0.62 (0.59-0.64) | |
| MCV (fL) declining | Prostate | 0.55 (0.52-0.59) | 0.50 (0.49-0.51) | 0.82 (0.80-0.84) | 0.83 (0.81-0.85) | |
| MCV (fL) declining | Stomach | 0.62 (0.57-0.66) | 0.53 (0.51-0.55) | 0.72 (0.69-0.75) | 0.70 (0.67-0.73) | |
| MCV (fL) declining | Thyroid | 0.59 (0.45-0.74) | 0.49 (0.49-0.49) | 0.60 (0.47-0.74) | 0.54 (0.41-0.67) | |
| MCV (fL) declining | Uterine | 0.69 (0.55-0.84) | 0.49 (0.49-0.49) | 0.49 (0.40-0.58) | 0.42 (0.33-0.51) | |
| RDW (%) increasing | Bladder | 0.53 (0.44-0.61) | 0.54 (0.47-0.61) | 0.70 (0.65-0.75) | 0.69 (0.64-0.74) | |
| RDW (%) increasing | Bowel | 0.60 (0.57-0.64) | 0.59 (0.56-0.63) | 0.65 (0.62-0.68) | 0.65 (0.63-0.68) | |
| RDW (%) increasing | Brain | 0.48 (0.33-0.63) | 0.41 (0.27-0.55) | 0.35 (0.23-0.47) | 0.34 (0.22-0.46) | |
| RDW (%) increasing | Breast | 0.51 (0.42-0.59) | 0.54 (0.45-0.63) | 0.47 (0.42-0.52) | 0.49 (0.44-0.55) | |
| RDW (%) increasing | HandN | 0.54 (0.45-0.64) | 0.49 (0.36-0.61) | 0.65 (0.54-0.76) | 0.65 (0.54-0.76) | |
| RDW (%) increasing | Kidney | 0.56 (0.48-0.64) | 0.55 (0.47-0.62) | 0.61 (0.56-0.67) | 0.61 (0.56-0.67) | |
| RDW (%) increasing | Leukaemia | 0.66 (0.56-0.77) | 0.65 (0.56-0.74) | 0.69 (0.62-0.76) | 0.68 (0.61-0.75) | |
| RDW (%) increasing | Liver | 0.57 (0.50-0.64) | 0.62 (0.54-0.69) | 0.68 (0.63-0.74) | 0.70 (0.65-0.75) | |
| RDW (%) increasing | Lung | 0.53 (0.50-0.56) | 0.52 (0.49-0.55) | 0.63 (0.61-0.65) | 0.63 (0.61-0.65) | |
| RDW (%) increasing | Lymphoma | 0.63 (0.57-0.69) | 0.63 (0.57-0.68) | 0.60 (0.55-0.64) | 0.61 (0.57-0.65) | |
| RDW (%) increasing | Melanoma | 0.58 (0.36-0.79) | 0.56 (0.36-0.76) | 0.75 (0.65-0.86) | 0.75 (0.65-0.85) | |
| RDW (%) increasing | Myeloma | 0.68 (0.60-0.77) | 0.62 (0.52-0.72) | 0.58 (0.51-0.65) | 0.60 (0.53-0.67) | |
| RDW (%) increasing | Oesophagus | 0.50 (0.44-0.56) | 0.48 (0.43-0.53) | 0.67 (0.63-0.72) | 0.67 (0.63-0.71) | |
| RDW (%) increasing | Ovarian | 0.42 (0.27-0.56) | 0.51 (0.40-0.62) | 0.40 (0.34-0.45) | 0.41 (0.36-0.47) | |
| RDW (%) increasing | Pancreas | 0.47 (0.43-0.51) | 0.49 (0.45-0.53) | 0.60 (0.57-0.64) | 0.59 (0.56-0.63) | |
| RDW (%) increasing | Prostate | 0.52 (0.46-0.57) | 0.52 (0.48-0.57) | 0.84 (0.82-0.86) | 0.83 (0.80-0.85) | |
| RDW (%) increasing | Stomach | 0.54 (0.48-0.60) | 0.54 (0.48-0.60) | 0.69 (0.64-0.73) | 0.69 (0.65-0.73) | |
| RDW (%) increasing | Thyroid | 0.51 (0.28-0.74) | 0.56 (0.36-0.76) | 0.63 (0.50-0.76) | 0.63 (0.49-0.78) | |
| RDW (%) increasing | Uterine | 0.69 (0.57-0.82) | 0.62 (0.45-0.79) | 0.38 (0.29-0.46) | 0.39 (0.31-0.47) | |
| Platelets (10^9^/L) increasing | Bladder | 0.59 (0.53-0.66) | 0.52 (0.47-0.56) | 0.73 (0.70-0.77) | 0.71 (0.67-0.75) | |
| Platelets (10^9^/L) increasing | Bowel | 0.66 (0.63-0.68) | 0.58 (0.55-0.60) | 0.73 (0.71-0.75) | 0.70 (0.69-0.72) | |
| Platelets (10^9^/L) increasing | Brain | 0.48 (0.37-0.59) | 0.46 (0.45-0.46) | 0.42 (0.31-0.53) | 0.40 (0.29-0.50) | |
| Platelets (10^9^/L) increasing | Breast | 0.50 (0.44-0.57) | 0.53 (0.48-0.57) | 0.50 (0.45-0.55) | 0.50 (0.46-0.55) | |
| Platelets (10^9^/L) increasing | HandN | 0.60 (0.50-0.71) | 0.53 (0.45-0.60) | 0.68 (0.61-0.76) | 0.67 (0.59-0.74) | |
| Platelets (10^9^/L) increasing | Kidney | 0.69 (0.64-0.74) | 0.59 (0.55-0.64) | 0.69 (0.65-0.73) | 0.66 (0.62-0.70) | |
| Platelets (10^9^/L) increasing | Leukaemia | 0.44 (0.34-0.53) | 0.48 (0.45-0.52) | 0.56 (0.50-0.63) | 0.64 (0.59-0.69) | |
| Platelets (10^9^/L) increasing | Liver | 0.55 (0.49-0.61) | 0.50 (0.47-0.53) | 0.67 (0.63-0.71) | 0.67 (0.63-0.71) | |
| Platelets (10^9^/L) increasing | Lung | 0.68 (0.66-0.70) | 0.58 (0.56-0.60) | 0.72 (0.71-0.74) | 0.69 (0.68-0.71) | |
| Platelets (10^9^/L) increasing | Lymphoma | 0.53 (0.48-0.58) | 0.55 (0.52-0.58) | 0.58 (0.55-0.61) | 0.59 (0.56-0.63) | |
| Platelets (10^9^/L) increasing | Melanoma | 0.45 (0.33-0.57) | 0.46 (0.45-0.46) | 0.66 (0.56-0.77) | 0.68 (0.58-0.78) | |
| Platelets (10^9^/L) increasing | Myeloma | 0.40 (0.32-0.47) | 0.48 (0.45-0.52) | 0.54 (0.49-0.60) | 0.57 (0.51-0.62) | |
| Platelets (10^9^/L) increasing | Oesophagus | 0.59 (0.55-0.64) | 0.50 (0.48-0.53) | 0.69 (0.66-0.73) | 0.68 (0.65-0.71) | |
| Platelets (10^9^/L) increasing | Ovarian | 0.58 (0.47-0.68) | 0.67 (0.59-0.75) | 0.54 (0.47-0.60) | 0.57 (0.51-0.63) | |
| Platelets (10^9^/L) increasing | Pancreas | 0.44 (0.41-0.47) | 0.49 (0.48-0.51) | 0.59 (0.57-0.61) | 0.61 (0.59-0.63) | |
| Platelets (10^9^/L) increasing | Prostate | 0.55 (0.51-0.59) | 0.51 (0.49-0.54) | 0.79 (0.77-0.81) | 0.81 (0.80-0.83) | |
| Platelets (10^9^/L) increasing | Stomach | 0.56 (0.52-0.61) | 0.52 (0.49-0.55) | 0.72 (0.69-0.75) | 0.71 (0.68-0.74) | |
| Platelets (10^9^/L) increasing | Thyroid | 0.59 (0.47-0.72) | 0.46 (0.46-0.46) | 0.56 (0.44-0.68) | 0.54 (0.44-0.65) | |
| Platelets (10^9^/L) increasing | Uterine | 0.56 (0.42-0.69) | 0.61 (0.50-0.73) | 0.49 (0.38-0.59) | 0.51 (0.40-0.62) | |
| MPV (fL) declining | Bladder | 0.56 (0.43-0.70) | 0.50 (0.44-0.57) | 0.71 (0.59-0.82) | 0.69 (0.58-0.81) | |
| MPV (fL) declining | Bowel | 0.54 (0.49-0.60) | 0.53 (0.51-0.54) | 0.66 (0.60-0.71) | 0.65 (0.60-0.70) | |
| MPV (fL) declining | Brain | 0.31 (0.25-0.37) | 0.54 (0.53-0.54) | 0.19 (0.16-0.22) | 0.22 (0.18-0.26) | |
| MPV (fL) declining | Breast | 0.48 (0.35-0.61) | 0.50 (0.43-0.57) | 0.55 (0.46-0.63) | 0.53 (0.43-0.63) | |
| MPV (fL) declining | HandN | 0.66 (0.44-0.87) | 0.54 (0.53-0.54) | 0.45 (0.13-0.77) | 0.41 (0.10-0.73) | |
| MPV (fL) declining | Kidney | 0.60 (0.47-0.73) | 0.54 (0.53-0.54) | 0.57 (0.45-0.69) | 0.54 (0.43-0.65) | |
| MPV (fL) declining | Leukaemia | 0.60 (0.45-0.75) | 0.54 (0.53-0.54) | 0.70 (0.58-0.82) | 0.71 (0.58-0.84) | |
| MPV (fL) declining | Liver | 0.49 (0.34-0.63) | 0.50 (0.44-0.57) | 0.70 (0.57-0.82) | 0.70 (0.58-0.82) | |
| MPV (fL) declining | Lung | 0.54 (0.49-0.59) | 0.53 (0.52-0.54) | 0.63 (0.58-0.67) | 0.62 (0.58-0.67) | |
| MPV (fL) declining | Lymphoma | 0.62 (0.53-0.71) | 0.52 (0.49-0.55) | 0.56 (0.49-0.64) | 0.53 (0.45-0.60) | |
| MPV (fL) declining | Melanoma | 0.58 (0.31-0.85) | 0.44 (0.24-0.63) | 0.92 (0.85-0.99) | 0.91 (0.82-0.99) | |
| MPV (fL) declining | Myeloma | 0.48 (0.31-0.64) | 0.54 (0.53-0.54) | 0.61 (0.47-0.75) | 0.57 (0.43-0.71) | |
| MPV (fL) declining | Oesophagus | 0.63 (0.55-0.71) | 0.54 (0.53-0.54) | 0.71 (0.64-0.78) | 0.69 (0.62-0.76) | |
| MPV (fL) declining | Ovarian | 0.44 (0.26-0.63) | 0.54 (0.53-0.54) | 0.47 (0.35-0.59) | 0.50 (0.38-0.62) | |
| MPV (fL) declining | Pancreas | 0.47 (0.40-0.54) | 0.48 (0.43-0.52) | 0.62 (0.56-0.68) | 0.63 (0.56-0.69) | |
| MPV (fL) declining | Prostate | 0.47 (0.39-0.54) | 0.52 (0.50-0.55) | 0.84 (0.80-0.88) | 0.86 (0.83-0.90) | |
| MPV (fL) declining | Stomach | 0.63 (0.56-0.70) | 0.54 (0.53-0.54) | 0.73 (0.67-0.80) | 0.71 (0.64-0.78) | |
| MPV (fL) declining | Thyroid | 0.54 (0.00-1.00) | 0.54 (0.53-0.54) | 0.73 (0.70-0.76) | 0.74 (0.59-0.88) | |
| MPV (fL) declining | Uterine | 0.48 (0.25-0.71) | 0.54 | 0.57 (0.33-0.81) | 0.55 (0.24-0.86) | |
| WBC (10^9^/L) increasing | Bladder | 0.65 (0.59-0.70) | 0.55 (0.50-0.60) | 0.73 (0.69-0.77) | 0.72 (0.68-0.76) | |
| WBC (10^9^/L) increasing | Bowel | 0.64 (0.62-0.67) | 0.57 (0.55-0.59) | 0.70 (0.68-0.72) | 0.68 (0.67-0.70) | |
| WBC (10^9^/L) increasing | Brain | 0.48 (0.35-0.61) | 0.45 (0.45-0.46) | 0.46 (0.35-0.56) | 0.43 (0.31-0.55) | |
| WBC (10^9^/L) increasing | Breast | 0.57 (0.52-0.63) | 0.55 (0.51-0.60) | 0.49 (0.45-0.54) | 0.50 (0.46-0.55) | |
| WBC (10^9^/L) increasing | HandN | 0.54 (0.45-0.63) | 0.51 (0.46-0.57) | 0.66 (0.59-0.72) | 0.63 (0.56-0.70) | |
| WBC (10^9^/L) increasing | Kidney | 0.60 (0.55-0.65) | 0.53 (0.49-0.56) | 0.62 (0.58-0.66) | 0.58 (0.54-0.62) | |
| WBC (10^9^/L) increasing | Leukaemia | 0.67 (0.57-0.76) | 0.64 (0.55-0.72) | 0.71 (0.64-0.78) | 0.74 (0.68-0.80) | |
| WBC (10^9^/L) increasing | Liver | 0.55 (0.48-0.61) | 0.53 (0.49-0.57) | 0.68 (0.64-0.72) | 0.68 (0.64-0.72) | |
| WBC (10^9^/L) increasing | Lung | 0.67 (0.65-0.69) | 0.58 (0.56-0.60) | 0.72 (0.70-0.73) | 0.68 (0.66-0.70) | |
| WBC (10^9^/L) increasing | Lymphoma | 0.49 (0.45-0.54) | 0.52 (0.50-0.55) | 0.57 (0.54-0.60) | 0.58 (0.55-0.61) | |
| WBC (10^9^/L) increasing | Melanoma | 0.55 (0.44-0.65) | 0.45 (0.45-0.46) | 0.69 (0.59-0.79) | 0.68 (0.58-0.78) | |
| WBC (10^9^/L) increasing | Myeloma | 0.38 (0.30-0.45) | 0.47 (0.44-0.49) | 0.50 (0.44-0.55) | 0.56 (0.51-0.60) | |
| WBC (10^9^/L) increasing | Oesophagus | 0.65 (0.60-0.69) | 0.54 (0.51-0.57) | 0.71 (0.68-0.74) | 0.69 (0.66-0.73) | |
| WBC (10^9^/L) increasing | Ovarian | 0.50 (0.41-0.58) | 0.48 (0.45-0.51) | 0.43 (0.38-0.49) | 0.44 (0.40-0.49) | |
| WBC (10^9^/L) increasing | Pancreas | 0.54 (0.51-0.57) | 0.52 (0.50-0.54) | 0.62 (0.60-0.65) | 0.62 (0.60-0.65) | |
| WBC (10^9^/L) increasing | Prostate | 0.53 (0.49-0.57) | 0.51 (0.49-0.53) | 0.80 (0.78-0.82) | 0.81 (0.80-0.83) | |
| WBC (10^9^/L) increasing | Stomach | 0.58 (0.54-0.62) | 0.52 (0.49-0.55) | 0.69 (0.66-0.73) | 0.68 (0.65-0.71) | |
| WBC (10^9^/L) increasing | Thyroid | 0.52 (0.37-0.67) | 0.55 (0.42-0.69) | 0.51 (0.38-0.64) | 0.53 (0.41-0.65) | |
| WBC (10^9^/L) increasing | Uterine | 0.63 (0.50-0.76) | 0.59 (0.47-0.70) | 0.52 (0.42-0.62) | 0.49 (0.39-0.59) | |
| Basophil count (10^9^/L) increasing | Bladder | 0.53 (0.46-0.60) | 0.50 (0.50-0.50) | 0.71 (0.67-0.75) | 0.70 (0.67-0.74) | |
| Basophil count (10^9^/L) increasing | Bowel | 0.49 (0.46-0.51) | 0.50 (0.50-0.50) | 0.65 (0.63-0.67) | 0.65 (0.63-0.67) | |
| Basophil count (10^9^/L) increasing | Brain | 0.55 (0.42-0.69) | 0.50 (0.50-0.50) | 0.42 (0.30-0.53) | 0.42 (0.30-0.53) | |
| Basophil count (10^9^/L) increasing | Breast | 0.49 (0.43-0.56) | 0.50 (0.50-0.50) | 0.46 (0.42-0.50) | 0.47 (0.42-0.51) | |
| Basophil count (10^9^/L) increasing | HandN | 0.53 (0.43-0.62) | 0.50 (0.50-0.50) | 0.61 (0.53-0.69) | 0.60 (0.52-0.69) | |
| Basophil count (10^9^/L) increasing | Kidney | 0.47 (0.41-0.53) | 0.50 (0.50-0.50) | 0.59 (0.55-0.63) | 0.59 (0.55-0.63) | |
| Basophil count (10^9^/L) increasing | Leukaemia | 0.55 (0.48-0.63) | 0.50 (0.50-0.50) | 0.66 (0.60-0.72) | 0.66 (0.60-0.72) | |
| Basophil count (10^9^/L) increasing | Liver | 0.50 (0.44-0.56) | 0.50 (0.50-0.50) | 0.66 (0.63-0.70) | 0.66 (0.62-0.70) | |
| Basophil count (10^9^/L) increasing | Lung | 0.53 (0.51-0.55) | 0.50 (0.50-0.50) | 0.64 (0.63-0.66) | 0.64 (0.63-0.66) | |
| Basophil count (10^9^/L) increasing | Lymphoma | 0.51 (0.47-0.56) | 0.50 (0.50-0.50) | 0.58 (0.54-0.61) | 0.57 (0.54-0.61) | |
| Basophil count (10^9^/L) increasing | Melanoma | 0.46 (0.33-0.59) | 0.50 (0.50-0.50) | 0.70 (0.59-0.81) | 0.70 (0.59-0.81) | |
| Basophil count (10^9^/L) increasing | Myeloma | 0.39 (0.31-0.48) | 0.50 (0.50-0.50) | 0.58 (0.52-0.63) | 0.58 (0.53-0.64) | |
| Basophil count (10^9^/L) increasing | Oesophagus | 0.55 (0.50-0.60) | 0.50 (0.50-0.50) | 0.68 (0.65-0.71) | 0.68 (0.64-0.71) | |
| Basophil count (10^9^/L) increasing | Ovarian | 0.43 (0.34-0.53) | 0.50 (0.50-0.50) | 0.43 (0.39-0.47) | 0.44 (0.39-0.48) | |
| Basophil count (10^9^/L) increasing | Pancreas | 0.49 (0.46-0.53) | 0.50 (0.50-0.50) | 0.62 (0.59-0.64) | 0.62 (0.59-0.64) | |
| Basophil count (10^9^/L) increasing | Prostate | 0.52 (0.48-0.56) | 0.50 (0.50-0.50) | 0.84 (0.82-0.86) | 0.84 (0.82-0.85) | |
| Basophil count (10^9^/L) increasing | Stomach | 0.48 (0.43-0.52) | 0.50 (0.50-0.50) | 0.67 (0.64-0.70) | 0.67 (0.64-0.70) | |
| Basophil count (10^9^/L) increasing | Thyroid | 0.46 (0.31-0.62) | 0.50 (0.50-0.50) | 0.52 (0.39-0.65) | 0.52 (0.39-0.65) | |
| Basophil count (10^9^/L) increasing | Uterine | 0.59 (0.44-0.73) | 0.50 (0.50-0.50) | 0.38 (0.30-0.46) | 0.38 (0.30-0.46) | |
| Eosinophil count (10^9^/L) increasing | Bladder | 0.51 (0.42-0.61) | 0.49 (0.46-0.52) | 0.69 (0.65-0.73) | 0.70 (0.66-0.74) | |
| Eosinophil count (10^9^/L) increasing | Bowel | 0.53 (0.49-0.56) | 0.51 (0.49-0.53) | 0.64 (0.63-0.66) | 0.65 (0.63-0.67) | |
| Eosinophil count (10^9^/L) increasing | Brain | 0.56 (0.35-0.77) | 0.52 (0.42-0.62) | 0.41 (0.31-0.52) | 0.42 (0.31-0.52) | |
| Eosinophil count (10^9^/L) increasing | Breast | 0.50 (0.42-0.58) | 0.47 (0.47-0.47) | 0.45 (0.41-0.49) | 0.47 (0.43-0.51) | |
| Eosinophil count (10^9^/L) increasing | HandN | 0.47 (0.32-0.62) | 0.50 (0.44-0.57) | 0.62 (0.54-0.70) | 0.62 (0.54-0.69) | |
| Eosinophil count (10^9^/L) increasing | Kidney | 0.44 (0.37-0.52) | 0.50 (0.47-0.53) | 0.59 (0.55-0.63) | 0.58 (0.54-0.62) | |
| Eosinophil count (10^9^/L) increasing | Leukaemia | 0.46 (0.34-0.59) | 0.49 (0.45-0.53) | 0.64 (0.59-0.70) | 0.64 (0.59-0.69) | |
| Eosinophil count (10^9^/L) increasing | Liver | 0.47 (0.40-0.54) | 0.50 (0.47-0.53) | 0.68 (0.64-0.71) | 0.67 (0.63-0.70) | |
| Eosinophil count (10^9^/L) increasing | Lung | 0.51 (0.48-0.54) | 0.50 (0.49-0.52) | 0.64 (0.62-0.66) | 0.64 (0.62-0.66) | |
| Eosinophil count (10^9^/L) increasing | Lymphoma | 0.48 (0.42-0.54) | 0.49 (0.47-0.52) | 0.58 (0.54-0.61) | 0.57 (0.54-0.60) | |
| Eosinophil count (10^9^/L) increasing | Melanoma | 0.32 (0.12-0.53) | 0.47 (0.47-0.47) | 0.73 (0.63-0.83) | 0.72 (0.62-0.82) | |
| Eosinophil count (10^9^/L) increasing | Myeloma | 0.43 (0.34-0.52) | 0.49 (0.46-0.52) | 0.59 (0.53-0.64) | 0.58 (0.53-0.63) | |
| Eosinophil count (10^9^/L) increasing | Oesophagus | 0.53 (0.47-0.60) | 0.51 (0.48-0.55) | 0.68 (0.65-0.71) | 0.68 (0.64-0.71) | |
| Eosinophil count (10^9^/L) increasing | Ovarian | 0.58 (0.49-0.67) | 0.49 (0.45-0.54) | 0.42 (0.38-0.47) | 0.44 (0.40-0.48) | |
| Eosinophil count (10^9^/L) increasing | Pancreas | 0.51 (0.46-0.55) | 0.50 (0.48-0.53) | 0.62 (0.60-0.65) | 0.62 (0.60-0.64) | |
| Eosinophil count (10^9^/L) increasing | Prostate | 0.47 (0.41-0.53) | 0.50 (0.47-0.52) | 0.85 (0.83-0.86) | 0.84 (0.82-0.85) | |
| Eosinophil count (10^9^/L) increasing | Stomach | 0.49 (0.43-0.55) | 0.50 (0.47-0.53) | 0.69 (0.66-0.72) | 0.68 (0.65-0.71) | |
| Eosinophil count (10^9^/L) increasing | Thyroid | 0.54 (0.34-0.75) | 0.55 (0.39-0.72) | 0.53 (0.42-0.65) | 0.53 (0.41-0.65) | |
| Eosinophil count (10^9^/L) increasing | Uterine | 0.44 (0.32-0.57) | 0.52 (0.42-0.62) | 0.34 (0.27-0.40) | 0.35 (0.29-0.42) | |
| Lymphocyte count (10^9^/L) increasing | Bladder | 0.61 (0.55-0.66) | 0.52 (0.47-0.58) | 0.70 (0.66-0.74) | 0.71 (0.68-0.74) | |
| Lymphocyte count (10^9^/L) increasing | Bowel | 0.51 (0.49-0.54) | 0.51 (0.49-0.53) | 0.65 (0.63-0.67) | 0.65 (0.64-0.67) | |
| Lymphocyte count (10^9^/L) increasing | Brain | 0.47 (0.35-0.59) | 0.51 (0.41-0.61) | 0.43 (0.32-0.54) | 0.55 (0.46-0.63) | |
| Lymphocyte count (10^9^/L) increasing | Breast | 0.49 (0.44-0.55) | 0.47 (0.45-0.49) | 0.45 (0.41-0.49) | 0.48 (0.45-0.51) | |
| Lymphocyte count (10^9^/L) increasing | HandN | 0.46 (0.36-0.57) | 0.48 (0.44-0.53) | 0.62 (0.54-0.70) | 0.64 (0.58-0.70) | |
| Lymphocyte count (10^9^/L) increasing | Kidney | 0.47 (0.42-0.53) | 0.51 (0.47-0.55) | 0.58 (0.54-0.62) | 0.61 (0.58-0.64) | |
| Lymphocyte count (10^9^/L) increasing | Leukaemia | 0.53 (0.44-0.61) | 0.51 (0.41-0.61) | 0.67 (0.61-0.74) | 0.69 (0.64-0.74) | |
| Lymphocyte count (10^9^/L) increasing | Liver | 0.46 (0.39-0.52) | 0.56 (0.50-0.61) | 0.67 (0.64-0.71) | 0.69 (0.66-0.72) | |
| Lymphocyte count (10^9^/L) increasing | Lung | 0.53 (0.51-0.55) | 0.50 (0.49-0.52) | 0.64 (0.62-0.66) | 0.66 (0.65-0.67) | |
| Lymphocyte count (10^9^/L) increasing | Lymphoma | 0.38 (0.34-0.43) | 0.59 (0.55-0.64) | 0.58 (0.55-0.61) | 0.62 (0.59-0.65) | |
| Lymphocyte count (10^9^/L) increasing | Melanoma | 0.34 (0.22-0.47) | 0.55 (0.43-0.67) | 0.72 (0.61-0.82) | 0.70 (0.62-0.78) | |
| Lymphocyte count (10^9^/L) increasing | Myeloma | 0.60 (0.52-0.68) | 0.48 (0.44-0.51) | 0.58 (0.52-0.63) | 0.61 (0.57-0.65) | |
| Lymphocyte count (10^9^/L) increasing | Oesophagus | 0.50 (0.46-0.54) | 0.50 (0.47-0.54) | 0.67 (0.64-0.71) | 0.69 (0.66-0.71) | |
| Lymphocyte count (10^9^/L) increasing | Ovarian | 0.44 (0.36-0.51) | 0.50 (0.45-0.55) | 0.43 (0.39-0.47) | 0.47 (0.44-0.51) | |
| Lymphocyte count (10^9^/L) increasing | Pancreas | 0.53 (0.50-0.56) | 0.51 (0.49-0.54) | 0.63 (0.60-0.65) | 0.65 (0.63-0.67) | |
| Lymphocyte count (10^9^/L) increasing | Prostate | 0.51 (0.48-0.55) | 0.52 (0.49-0.55) | 0.85 (0.83-0.86) | 0.84 (0.83-0.86) | |
| Lymphocyte count (10^9^/L) increasing | Stomach | 0.52 (0.48-0.56) | 0.52 (0.49-0.55) | 0.69 (0.66-0.72) | 0.71 (0.69-0.73) | |
| Lymphocyte count (10^9^/L) increasing | Thyroid | 0.48 (0.33-0.62) | 0.46 (0.46-0.46) | 0.55 (0.43-0.68) | 0.52 (0.41-0.63) | |
| Lymphocyte count (10^9^/L) increasing | Uterine | 0.42 (0.31-0.53) | 0.46 (0.46-0.46) | 0.36 (0.29-0.44) | 0.45 (0.39-0.51) | |
| Monocyte count (10^9^/L) increasing | Bladder | 0.61 (0.55-0.67) | 0.49 (0.45-0.54) | 0.72 (0.68-0.77) | 0.70 (0.66-0.75) | |
| Monocyte count (10^9^/L) increasing | Bowel | 0.59 (0.57-0.62) | 0.57 (0.55-0.59) | 0.68 (0.67-0.70) | 0.68 (0.66-0.70) | |
| Monocyte count (10^9^/L) increasing | Brain | 0.42 (0.33-0.50) | 0.44 (0.44-0.44) | 0.40 (0.29-0.51) | 0.40 (0.30-0.50) | |
| Monocyte count (10^9^/L) increasing | Breast | 0.51 (0.45-0.57) | 0.52 (0.48-0.56) | 0.48 (0.44-0.53) | 0.50 (0.46-0.55) | |
| Monocyte count (10^9^/L) increasing | HandN | 0.54 (0.45-0.64) | 0.53 (0.46-0.60) | 0.65 (0.57-0.73) | 0.64 (0.56-0.71) | |
| Monocyte count (10^9^/L) increasing | Kidney | 0.60 (0.54-0.65) | 0.55 (0.51-0.60) | 0.62 (0.57-0.66) | 0.60 (0.55-0.64) | |
| Monocyte count (10^9^/L) increasing | Leukaemia | 0.51 (0.41-0.62) | 0.53 (0.45-0.60) | 0.63 (0.56-0.69) | 0.66 (0.60-0.73) | |
| Monocyte count (10^9^/L) increasing | Liver | 0.52 (0.46-0.58) | 0.56 (0.51-0.61) | 0.69 (0.65-0.73) | 0.69 (0.65-0.72) | |
| Monocyte count (10^9^/L) increasing | Lung | 0.63 (0.61-0.65) | 0.59 (0.57-0.60) | 0.69 (0.68-0.71) | 0.67 (0.66-0.69) | |
| Monocyte count (10^9^/L) increasing | Lymphoma | 0.60 (0.56-0.65) | 0.55 (0.52-0.59) | 0.61 (0.57-0.64) | 0.60 (0.57-0.64) | |
| Monocyte count (10^9^/L) increasing | Melanoma | 0.53 (0.43-0.63) | 0.53 (0.43-0.62) | 0.70 (0.59-0.80) | 0.72 (0.61-0.82) | |
| Monocyte count (10^9^/L) increasing | Myeloma | 0.46 (0.38-0.54) | 0.53 (0.47-0.58) | 0.56 (0.51-0.62) | 0.59 (0.53-0.64) | |
| Monocyte count (10^9^/L) increasing | Oesophagus | 0.59 (0.54-0.64) | 0.56 (0.52-0.60) | 0.70 (0.66-0.73) | 0.69 (0.66-0.72) | |
| Monocyte count (10^9^/L) increasing | Ovarian | 0.53 (0.44-0.62) | 0.59 (0.52-0.65) | 0.49 (0.43-0.55) | 0.52 (0.46-0.58) | |
| Monocyte count (10^9^/L) increasing | Pancreas | 0.51 (0.48-0.54) | 0.53 (0.51-0.55) | 0.62 (0.60-0.64) | 0.62 (0.60-0.65) | |
| Monocyte count (10^9^/L) increasing | Prostate | 0.53 (0.49-0.57) | 0.53 (0.51-0.56) | 0.81 (0.79-0.83) | 0.81 (0.79-0.83) | |
| Monocyte count (10^9^/L) increasing | Stomach | 0.52 (0.48-0.57) | 0.52 (0.49-0.55) | 0.67 (0.64-0.70) | 0.67 (0.64-0.70) | |
| Monocyte count (10^9^/L) increasing | Thyroid | 0.56 (0.35-0.77) | 0.59 (0.44-0.74) | 0.55 (0.41-0.69) | 0.57 (0.41-0.72) | |
| Monocyte count (10^9^/L) increasing | Uterine | 0.64 (0.53-0.75) | 0.56 (0.47-0.66) | 0.44 (0.35-0.53) | 0.42 (0.33-0.51) | |
| Neutrophil count (10^9^/L) increasing | Bladder | 0.64 (0.58-0.70) | 0.57 (0.52-0.63) | 0.75 (0.71-0.79) | 0.74 (0.70-0.78) | |
| Neutrophil count (10^9^/L) increasing | Bowel | 0.64 (0.62-0.67) | 0.57 (0.55-0.59) | 0.71 (0.69-0.73) | 0.69 (0.67-0.71) | |
| Neutrophil count (10^9^/L) increasing | Brain | 0.49 (0.39-0.59) | 0.45 (0.44-0.45) | 0.46 (0.34-0.57) | 0.43 (0.31-0.54) | |
| Neutrophil count (10^9^/L) increasing | Breast | 0.59 (0.52-0.65) | 0.56 (0.51-0.61) | 0.51 (0.46-0.55) | 0.51 (0.46-0.56) | |
| Neutrophil count (10^9^/L) increasing | HandN | 0.61 (0.52-0.71) | 0.59 (0.51-0.67) | 0.66 (0.59-0.72) | 0.66 (0.59-0.72) | |
| Neutrophil count (10^9^/L) increasing | Kidney | 0.60 (0.55-0.65) | 0.54 (0.50-0.57) | 0.63 (0.59-0.67) | 0.60 (0.56-0.64) | |
| Neutrophil count (10^9^/L) increasing | Leukaemia | 0.49 (0.40-0.59) | 0.57 (0.50-0.63) | 0.60 (0.53-0.66) | 0.65 (0.59-0.71) | |
| Neutrophil count (10^9^/L) increasing | Liver | 0.55 (0.50-0.61) | 0.54 (0.50-0.58) | 0.68 (0.65-0.72) | 0.69 (0.65-0.73) | |
| Neutrophil count (10^9^/L) increasing | Lung | 0.68 (0.66-0.70) | 0.61 (0.59-0.63) | 0.72 (0.70-0.73) | 0.64 (0.63-0.65) | |
| Neutrophil count (10^9^/L) increasing | Lymphoma | 0.52 (0.47-0.56) | 0.53 (0.50-0.56) | 0.57 (0.53-0.60) | 0.58 (0.54-0.61) | |
| Neutrophil count (10^9^/L) increasing | Melanoma | 0.64 (0.54-0.74) | 0.45 (0.45-0.45) | 0.74 (0.64-0.83) | 0.69 (0.59-0.80) | |
| Neutrophil count (10^9^/L) increasing | Myeloma | 0.34 (0.26-0.42) | 0.46 (0.43-0.48) | 0.48 (0.42-0.54) | 0.54 (0.49-0.59) | |
| Neutrophil count (10^9^/L) increasing | Oesophagus | 0.63 (0.59-0.68) | 0.55 (0.51-0.58) | 0.71 (0.68-0.74) | 0.69 (0.66-0.72) | |
| Neutrophil count (10^9^/L) increasing | Ovarian | 0.53 (0.45-0.61) | 0.50 (0.45-0.54) | 0.47 (0.42-0.53) | 0.45 (0.41-0.50) | |
| Neutrophil count (10^9^/L) increasing | Pancreas | 0.55 (0.52-0.58) | 0.52 (0.50-0.54) | 0.63 (0.61-0.65) | 0.63 (0.61-0.66) | |
| Neutrophil count (10^9^/L) increasing | Prostate | 0.54 (0.50-0.58) | 0.53 (0.50-0.56) | 0.80 (0.78-0.82) | 0.81 (0.79-0.82) | |
| Neutrophil count (10^9^/L) increasing | Stomach | 0.55 (0.50-0.59) | 0.53 (0.50-0.56) | 0.70 (0.67-0.73) | 0.69 (0.66-0.72) | |
| Neutrophil count (10^9^/L) increasing | Thyroid | 0.57 (0.43-0.72) | 0.54 (0.42-0.66) | 0.51 (0.38-0.64) | 0.55 (0.43-0.67) | |
| Neutrophil count (10^9^/L) increasing | Uterine | 0.72 (0.61-0.83) | 0.61 (0.49-0.74) | 0.49 (0.39-0.60) | 0.45 (0.34-0.55) | |
| CRP (mg/L) increasing | Bladder | 0.69 (0.56-0.81) | 0.60 (0.51-0.69) | 0.78 (0.68-0.89) | 0.75 (0.66-0.84) | |
| CRP (mg/L) increasing | Bowel | 0.66 (0.62-0.70) | 0.61 (0.58-0.64) | 0.72 (0.68-0.75) | 0.69 (0.65-0.72) | |
| CRP (mg/L) increasing | Brain | 0.36 (0.17-0.56) | 0.47 (0.28-0.67) | 0.35 (0.21-0.49) | 0.37 (0.22-0.52) | |
| CRP (mg/L) increasing | Breast | 0.55 (0.45-0.64) | 0.56 (0.48-0.64) | 0.44 (0.35-0.52) | 0.47 (0.39-0.55) | |
| CRP (mg/L) increasing | HandN | 0.67 (0.50-0.85) | 0.63 (0.50-0.75) | 0.75 (0.51-0.99) | 0.70 (0.50-0.91) | |
| CRP (mg/L) increasing | Kidney | 0.74 (0.65-0.83) | 0.59 (0.53-0.66) | 0.75 (0.67-0.82) | 0.63 (0.55-0.72) | |
| CRP (mg/L) increasing | Leukaemia | 0.59 (0.46-0.72) | 0.53 (0.40-0.67) | 0.72 (0.59-0.85) | 0.69 (0.55-0.82) | |
| CRP (mg/L) increasing | Liver | 0.67 (0.58-0.76) | 0.63 (0.58-0.69) | 0.77 (0.70-0.84) | 0.75 (0.68-0.82) | |
| CRP (mg/L) increasing | Lung | 0.70 (0.67-0.73) | 0.62 (0.59-0.64) | 0.73 (0.69-0.76) | 0.68 (0.65-0.71) | |
| CRP (mg/L) increasing | Lymphoma | 0.62 (0.55-0.68) | 0.61 (0.57-0.65) | 0.64 (0.58-0.70) | 0.62 (0.57-0.68) | |
| CRP (mg/L) increasing | Melanoma | 0.37 (0.13-0.62) | 0.35 (0.15-0.56) | 0.64 (0.43-0.85) | 0.57 (0.32-0.82) | |
| CRP (mg/L) increasing | Myeloma | 0.68 (0.55-0.81) | 0.53 (0.42-0.64) | 0.68 (0.60-0.76) | 0.57 (0.49-0.65) | |
| CRP (mg/L) increasing | Oesophagus | 0.52 (0.45-0.59) | 0.59 (0.53-0.64) | 0.71 (0.65-0.76) | 0.72 (0.65-0.78) | |
| CRP (mg/L) increasing | Ovarian | 0.56 (0.44-0.68) | 0.57 (0.45-0.69) | 0.46 (0.33-0.60) | 0.50 (0.38-0.62) | |
| CRP (mg/L) increasing | Pancreas | 0.50 (0.45-0.55) | 0.54 (0.50-0.58) | 0.61 (0.57-0.65) | 0.62 (0.57-0.66) | |
| CRP (mg/L) increasing | Prostate | 0.55 (0.47-0.62) | 0.54 (0.48-0.59) | 0.79 (0.75-0.83) | 0.77 (0.71-0.83) | |
| CRP (mg/L) increasing | Stomach | 0.50 (0.42-0.57) | 0.57 (0.51-0.64) | 0.63 (0.57-0.70) | 0.67 (0.60-0.75) | |
| CRP (mg/L) increasing | Thyroid | 0.58 (0.37-0.79) | 0.69 (0.68-0.69) | 0.58 (0.48-0.68) | 0.74 (0.67-0.82) | |
| CRP (mg/L) increasing | Uterine | 0.67 (0.41-0.94) | 0.69 (0.68-0.69) | 0.76 (0.48-1.00) | 0.65 (0.45-0.84) | |
| ESR (mm/h) increasing | Bladder | 0.69 (0.56-0.81) | 0.67 (0.56-0.77) | 0.78 (0.68-0.89) | 0.79 (0.69-0.89) | |
| ESR (mm/h) increasing | Bowel | 0.66 (0.62-0.70) | 0.65 (0.62-0.69) | 0.72 (0.68-0.75) | 0.71 (0.68-0.75) | |
| ESR (mm/h) increasing | Brain | 0.37 (0.17-0.56) | 0.43 (0.25-0.61) | 0.35 (0.21-0.49) | 0.36 (0.18-0.53) | |
| ESR (mm/h) increasing | Breast | 0.55 (0.45-0.64) | 0.51 (0.42-0.60) | 0.44 (0.35-0.52) | 0.46 (0.37-0.55) | |
| ESR (mm/h) increasing | HandN | 0.67 (0.50-0.85) | 0.72 (0.60-0.85) | 0.75 (0.51-0.99) | 0.74 (0.55-0.94) | |
| ESR (mm/h) increasing | Kidney | 0.74 (0.65-0.83) | 0.65 (0.57-0.73) | 0.75 (0.67-0.82) | 0.68 (0.61-0.75) | |
| ESR (mm/h) increasing | Leukaemia | 0.59 (0.46-0.72) | 0.59 (0.46-0.73) | 0.72 (0.59-0.85) | 0.72 (0.58-0.86) | |
| ESR (mm/h) increasing | Liver | 0.67 (0.58-0.76) | 0.64 (0.57-0.72) | 0.77 (0.70-0.84) | 0.74 (0.68-0.81) | |
| ESR (mm/h) increasing | Lung | 0.70 (0.67-0.73) | 0.67 (0.64-0.70) | 0.73 (0.69-0.76) | 0.72 (0.69-0.75) | |
| ESR (mm/h) increasing | Lymphoma | 0.62 (0.55-0.68) | 0.62 (0.56-0.67) | 0.64 (0.58-0.70) | 0.62 (0.56-0.68) | |
| ESR (mm/h) increasing | Melanoma | 0.37 (0.13-0.62) | 0.45 (0.25-0.66) | 0.64 (0.43-0.85) | 0.65 (0.44-0.86) | |
| ESR (mm/h) increasing | Myeloma | 0.71 (0.60-0.82) | 0.63 (0.52-0.74) | 0.69 (0.61-0.78) | 0.66 (0.57-0.76) | |
| ESR (mm/h) increasing | Oesophagus | 0.52 (0.45-0.59) | 0.60 (0.53-0.67) | 0.71 (0.65-0.76) | 0.72 (0.65-0.78) | |
| ESR (mm/h) increasing | Ovarian | 0.56 (0.44-0.68) | 0.52 (0.38-0.66) | 0.46 (0.33-0.60) | 0.48 (0.33-0.63) | |
| ESR (mm/h) increasing | Pancreas | 0.50 (0.45-0.55) | 0.52 (0.47-0.56) | 0.61 (0.57-0.65) | 0.60 (0.56-0.65) | |
| ESR (mm/h) increasing | Prostate | 0.55 (0.48-0.62) | 0.56 (0.50-0.62) | 0.79 (0.75-0.83) | 0.76 (0.71-0.81) | |
| ESR (mm/h) increasing | Stomach | 0.50 (0.42-0.57) | 0.55 (0.47-0.62) | 0.63 (0.56-0.70) | 0.65 (0.58-0.72) | |
| ESR (mm/h) increasing | Thyroid | 0.58 (0.37-0.79) | 0.62 (0.29-0.95) | 0.58 (0.48-0.68) | 0.68 (0.40-0.97) | |
| ESR (mm/h) increasing | Uterine | 0.67 (0.40-0.94) | 0.62 (0.29-0.95) | 0.76 (0.48-1.00) | 0.63 (0.42-0.84) | |
| Plasma viscosity (mPa.S) increasing | Bladder | 0.85 (0.72-0.98) | 0.81 (0.80-0.82) | 0.97 (0.93-1.00) | 0.99 (0.97-1.00) | |
| Plasma viscosity (mPa.S) increasing | Bowel | 0.58 (0.47-0.69) | 0.57 (0.46-0.68) | 0.65 (0.54-0.77) | 0.66 (0.56-0.77) | |
| Plasma viscosity (mPa.S) increasing | Breast | 0.53 (0.32-0.75) | 0.64 (0.32-0.97) | 0.65 (0.50-0.79) | 0.65 (0.33-0.97) | |
| Plasma viscosity (mPa.S) increasing | HandN | 0.58 (0.50-0.67) | 0.31 | 0.59 | 0.43 | |
| Plasma viscosity (mPa.S) increasing | Kidney | 0.85 (0.70-1.00) | 0.75 (0.62-0.87) | 0.79 (0.60-0.98) | 0.79 (0.62-0.97) | |
| Plasma viscosity (mPa.S) increasing | Leukaemia | 0.66 | 0.81 | 0.69 | 0.84 | |
| Plasma viscosity (mPa.S) increasing | Liver | 0.58 (0.38-0.77) | 0.64 (0.32-0.97) | 0.70 (0.45-0.94) | 0.76 (0.53-0.99) | |
| Plasma viscosity (mPa.S) increasing | Lung | 0.66 (0.58-0.74) | 0.64 (0.56-0.73) | 0.71 (0.63-0.79) | 0.68 (0.59-0.77) | |
| Plasma viscosity (mPa.S) increasing | Lymphoma | 0.69 (0.56-0.82) | 0.66 (0.53-0.79) | 0.65 (0.47-0.83) | 0.67 (0.50-0.84) | |
| Plasma viscosity (mPa.S) increasing | Myeloma | 0.98 (0.97-1.00) | 0.81 (0.80-0.82) | 0.94 (0.85-1.00) | 0.89 (0.81-0.96) | |
| Plasma viscosity (mPa.S) increasing | Oesophagus | 0.39 (0.17-0.61) | 0.48 (0.31-0.64) | 0.40 (0.23-0.58) | 0.49 (0.31-0.66) | |
| Plasma viscosity (mPa.S) increasing | Ovarian | 0.74 (0.55-0.94) | 0.67 (0.49-0.85) | 0.60 (0.37-0.82) | 0.54 (0.40-0.69) | |
| Plasma viscosity (mPa.S) increasing | Pancreas | 0.70 (0.58-0.82) | 0.64 (0.50-0.78) | 0.75 (0.64-0.86) | 0.74 (0.62-0.87) | |
| Plasma viscosity (mPa.S) increasing | Prostate | 0.55 (0.33-0.77) | 0.59 (0.40-0.79) | 0.70 (0.58-0.82) | 0.76 (0.64-0.87) | |
| Plasma viscosity (mPa.S) increasing | Stomach | 0.40 (0.24-0.57) | 0.48 (0.27-0.68) | 0.69 (0.56-0.81) | 0.67 (0.52-0.82) | |
| Plasma viscosity (mPa.S) increasing | Uterine | 0.78 (0.41-1.00) | 0.81 (0.80-0.82) | 0.80 (0.47-1.00) | 0.69 (0.55-0.83) | |
| NLR increasing | Bladder | 0.55 (0.48-0.62) | 0.55 (0.48-0.62) | 0.72 (0.68-0.76) | 0.70 (0.66-0.74) | |
| NLR increasing | Bowel | 0.58 (0.56-0.61) | 0.59 (0.57-0.62) | 0.69 (0.67-0.71) | 0.68 (0.66-0.69) | |
| NLR increasing | Brain | 0.52 (0.39-0.64) | 0.47 (0.35-0.60) | 0.45 (0.33-0.57) | 0.43 (0.31-0.55) | |
| NLR increasing | Breast | 0.50 (0.43-0.57) | 0.57 (0.50-0.63) | 0.51 (0.46-0.55) | 0.51 (0.46-0.55) | |
| NLR increasing | HandN | 0.59 (0.49-0.69) | 0.59 (0.49-0.70) | 0.64 (0.56-0.71) | 0.63 (0.55-0.71) | |
| NLR increasing | Kidney | 0.61 (0.56-0.66) | 0.58 (0.53-0.63) | 0.62 (0.58-0.66) | 0.60 (0.56-0.64) | |
| NLR increasing | Leukaemia | 0.41 (0.30-0.53) | 0.57 (0.47-0.68) | 0.60 (0.52-0.69) | 0.66 (0.58-0.74) | |
| NLR increasing | Liver | 0.55 (0.49-0.61) | 0.64 (0.59-0.69) | 0.69 (0.65-0.73) | 0.70 (0.66-0.73) | |
| NLR increasing | Lung | 0.60 (0.58-0.62) | 0.62 (0.59-0.64) | 0.68 (0.67-0.70) | 0.67 (0.66-0.69) | |
| NLR increasing | Lymphoma | 0.60 (0.55-0.65) | 0.64 (0.60-0.68) | 0.62 (0.59-0.65) | 0.62 (0.59-0.65) | |
| NLR increasing | Melanoma | 0.69 (0.60-0.77) | 0.57 (0.44-0.70) | 0.77 (0.68-0.87) | 0.75 (0.65-0.84) | |
| NLR increasing | Myeloma | 0.33 (0.24-0.41) | 0.46 (0.39-0.54) | 0.49 (0.43-0.54) | 0.53 (0.48-0.58) | |
| NLR increasing | Oesophagus | 0.57 (0.53-0.62) | 0.55 (0.50-0.59) | 0.69 (0.65-0.72) | 0.68 (0.65-0.71) | |
| NLR increasing | Ovarian | 0.59 (0.51-0.66) | 0.54 (0.46-0.62) | 0.50 (0.45-0.56) | 0.47 (0.42-0.52) | |
| NLR increasing | Pancreas | 0.51 (0.48-0.55) | 0.55 (0.52-0.58) | 0.62 (0.60-0.64) | 0.63 (0.61-0.65) | |
| NLR increasing | Prostate | 0.53 (0.49-0.57) | 0.56 (0.51-0.60) | 0.80 (0.78-0.82) | 0.81 (0.79-0.83) | |
| NLR increasing | Stomach | 0.54 (0.50-0.58) | 0.57 (0.52-0.61) | 0.68 (0.65-0.71) | 0.69 (0.66-0.72) | |
| NLR increasing | Thyroid | 0.64 (0.52-0.76) | 0.51 (0.34-0.67) | 0.52 (0.40-0.65) | 0.52 (0.40-0.65) | |
| NLR increasing | Uterine | 0.63 (0.51-0.76) | 0.58 (0.42-0.74) | 0.47 (0.37-0.57) | 0.42 (0.34-0.51) | |
| PLR increasing | Bladder | 0.41 (0.29-0.53) | 0.47 (0.32-0.61) | 0.71 (0.67-0.75) | 0.72 (0.68-0.75) | |
| PLR increasing | Bowel | 0.63 (0.59-0.67) | 0.69 (0.65-0.74) | 0.69 (0.67-0.70) | 0.70 (0.69-0.72) | |
| PLR increasing | Brain | 0.31 (0.05-0.57) | 0.53 (0.13-0.92) | 0.44 (0.32-0.56) | 0.44 (0.31-0.56) | |
| PLR increasing | Breast | 0.62 (0.49-0.74) | 0.69 (0.55-0.84) | 0.45 (0.41-0.49) | 0.50 (0.45-0.54) | |
| PLR increasing | HandN | 0.59 (0.38-0.81) | 0.60 (0.30-0.89) | 0.66 (0.58-0.75) | 0.67 (0.59-0.75) | |
| PLR increasing | Kidney | 0.75 (0.67-0.83) | 0.79 (0.72-0.85) | 0.65 (0.61-0.70) | 0.67 (0.62-0.71) | |
| PLR increasing | Leukaemia | 0.40 (0.21-0.59) | 0.29 (0.05-0.53) | 0.62 (0.55-0.70) | 0.59 (0.51-0.66) | |
| PLR increasing | Liver | 0.52 (0.40-0.63) | 0.60 (0.48-0.71) | 0.69 (0.65-0.72) | 0.69 (0.66-0.73) | |
| PLR increasing | Lung | 0.62 (0.58-0.66) | 0.68 (0.64-0.71) | 0.68 (0.66-0.70) | 0.70 (0.68-0.71) | |
| PLR increasing | Lymphoma | 0.68 (0.60-0.76) | 0.66 (0.57-0.75) | 0.63 (0.59-0.66) | 0.64 (0.61-0.68) | |
| PLR increasing | Melanoma | 0.54 (0.26-0.83) | 0.64 (0.42-0.87) | 0.72 (0.62-0.82) | 0.72 (0.62-0.81) | |
| PLR increasing | Myeloma | 0.36 (0.20-0.52) | 0.41 (0.24-0.57) | 0.53 (0.47-0.59) | 0.52 (0.47-0.58) | |
| PLR increasing | Oesophagus | 0.52 (0.44-0.60) | 0.54 (0.45-0.62) | 0.69 (0.66-0.72) | 0.70 (0.66-0.73) | |
| PLR increasing | Ovarian | 0.61 (0.44-0.78) | 0.63 (0.47-0.79) | 0.49 (0.43-0.54) | 0.56 (0.50-0.63) | |
| PLR increasing | Pancreas | 0.38 (0.33-0.43) | 0.45 (0.39-0.51) | 0.61 (0.59-0.64) | 0.62 (0.59-0.64) | |
| PLR increasing | Prostate | 0.53 (0.47-0.60) | 0.54 (0.46-0.62) | 0.82 (0.81-0.83) | 0.78 (0.77-0.79) | |
| PLR increasing | Stomach | 0.49 (0.42-0.57) | 0.59 (0.51-0.67) | 0.70 (0.67-0.73) | 0.71 (0.68-0.73) | |
| PLR increasing | Thyroid | 0.57 (0.43-0.71) | 0.60 (0.45-0.75) | 0.56 (0.44-0.68) | 0.55 (0.43-0.67) | |
| PLR increasing | Uterine | 0.52 (0.29-0.74) | 0.60 (0.29-0.90) | 0.38 (0.30-0.45) | 0.45 (0.35-0.54) | |
| mGPR increasing | Bladder | 0.64 (0.48-0.80) | 0.58 (0.46-0.70) | 0.75 (0.62-0.88) | 0.71 (0.61-0.81) | |
| mGPR increasing | Bowel | 0.65 (0.60-0.71) | 0.65 (0.60-0.70) | 0.71 (0.67-0.76) | 0.69 (0.65-0.74) | |
| mGPR increasing | Brain | 0.26 (0.10-0.42) | 0.44 (0.18-0.69) | 0.26 (0.13-0.39) | 0.35 (0.15-0.55) | |
| mGPR increasing | Breast | 0.55 (0.43-0.66) | 0.54 (0.43-0.65) | 0.49 (0.38-0.59) | 0.49 (0.39-0.58) | |
| mGPR increasing | HandN | 0.65 (0.42-0.87) | 0.69 (0.42-0.95) | 0.65 (0.28-1.00) | 0.65 (0.30-1.00) | |
| mGPR increasing | Kidney | 0.71 (0.58-0.84) | 0.64 (0.51-0.77) | 0.76 (0.67-0.84) | 0.67 (0.57-0.77) | |
| mGPR increasing | Leukaemia | 0.56 (0.37-0.75) | 0.51 (0.32-0.70) | 0.66 (0.42-0.91) | 0.59 (0.38-0.81) | |
| mGPR increasing | Liver | 0.66 (0.53-0.78) | 0.63 (0.51-0.75) | 0.79 (0.68-0.89) | 0.76 (0.65-0.87) | |
| mGPR increasing | Lung | 0.71 (0.67-0.74) | 0.65 (0.61-0.69) | 0.71 (0.67-0.76) | 0.70 (0.66-0.74) | |
| mGPR increasing | Lymphoma | 0.59 (0.51-0.68) | 0.62 (0.55-0.69) | 0.60 (0.52-0.68) | 0.60 (0.52-0.68) | |
| mGPR increasing | Melanoma | 0.45 (0.14-0.77) | 0.39 (0.17-0.61) | 0.64 (0.39-0.90) | 0.59 (0.32-0.86) | |
| mGPR increasing | Myeloma | 0.72 (0.55-0.90) | 0.62 (0.45-0.79) | 0.71 (0.58-0.83) | 0.60 (0.46-0.74) | |
| mGPR increasing | Oesophagus | 0.54 (0.46-0.63) | 0.63 (0.54-0.71) | 0.74 (0.67-0.80) | 0.73 (0.66-0.81) | |
| mGPR increasing | Ovarian | 0.49 (0.35-0.63) | 0.63 (0.44-0.83) | 0.40 (0.27-0.53) | 0.56 (0.39-0.73) | |
| mGPR increasing | Pancreas | 0.48 (0.41-0.54) | 0.51 (0.44-0.58) | 0.59 (0.53-0.65) | 0.59 (0.52-0.65) | |
| mGPR increasing | Prostate | 0.56 (0.48-0.65) | 0.58 (0.49-0.66) | 0.80 (0.75-0.85) | 0.77 (0.71-0.83) | |
| mGPR increasing | Stomach | 0.47 (0.38-0.56) | 0.60 (0.50-0.69) | 0.64 (0.56-0.73) | 0.69 (0.61-0.77) | |
| mGPR increasing | Thyroid | 0.50 (0.35-0.66) | 0.76 (0.57-0.95) | 0.60 (0.48-0.71) | 0.77 (0.59-0.95) | |
| mGPR increasing | Uterine | 0.58 (0.30-0.85) | 0.95 | 0.53 | 0.93 | |
| This table shows the AUC of unadjusted and age- and sex-adjusted one-year trend for individual cancer sites diagnosed over the six-month follow-up period after UWL. For some cancer sites, CIs are missing because there were insufficient events to estimate a standard error and therefore the CI – these AUCs in particular should be interpreted with caution.  Abbreviations: UWL=unexpected weight loss: ALT=alanine aminotransferase; AST=aspartate aminotransferase; RBC=red blood cell count; MCH=mean cell haemoglobin; MCHC=mean cell haemoglobin concentration; MCV=mean cell volume; RDW=red cell distribution width; MPV=mean platelet volume; WBC=white blood cell count; CRP=C-reactive protein; ESR=erythrocyte sedimentation rate; NLR=neutrophil-lymphocyte ratio; PLR=platelet-lymphocyte ratio; mGPR=modified Glasgow Prognostic ratio. | | | | | | |

# Table G: AUC (95% CI) of 1-year trend by number of repeat tests per person

| Blood test | No. of repeat tests per person | AUC unadjusted | AUC adjusted (age and sex) | |
| --- | --- | --- | --- | --- |
| Albumin | 2 | 0.59 (0.58-0.60) | 0.69 (0.68-0.70) | |
| Albumin | 3 | 0.58 (0.56-0.60) | 0.66 (0.64-0.68) | |
| Albumin | 4 | 0.54 (0.51-0.58) | 0.63 (0.60-0.66) | |
| Albumin | 5 | 0.54 (0.48-0.61) | 0.66 (0.60-0.71) | |
| Albumin | 6 | 0.54 (0.43-0.65) | 0.66 (0.59-0.73) | |
| Albumin | 7 | 0.51 (0.35-0.67) | 0.67 (0.57-0.76) | |
| Albumin | 8 | 0.59 (0.28-0.90) | 0.72 (0.50-0.93) | |
| Albumin | 9 | 0.39 (0.13-0.65) | 0.55 (0.40-0.70) | |
| Albumin | 10 | 0.60 (0.50-0.69) | 0.65 (0.57-0.73) | |
| ALP | 2 | 0.60 (0.59-0.61) | 0.69 (0.67-0.70) | |
| ALP | 3 | 0.59 (0.57-0.61) | 0.65 (0.63-0.67) | |
| ALP | 4 | 0.57 (0.53-0.61) | 0.65 (0.62-0.68) | |
| ALP | 5 | 0.56 (0.49-0.64) | 0.66 (0.61-0.71) | |
| ALP | 6 | 0.55 (0.44-0.66) | 0.69 (0.62-0.75) | |
| ALP | 7 | 0.56 (0.34-0.78) | 0.75 (0.61-0.89) | |
| ALP | 8 | 0.57 (0.37-0.77) | 0.67 (0.52-0.82) | |
| ALP | 9 | 0.51 (0.28-0.74) | 0.59 (0.46-0.72) | |
| ALP | 10 | 0.57 (0.48-0.66) | 0.65 (0.58-0.73) | |
| ALT | 2 | 0.52 (0.51-0.53) | 0.65 (0.64-0.66) | |
| ALT | 3 | 0.53 (0.51-0.55) | 0.63 (0.61-0.65) | |
| ALT | 4 | 0.53 (0.49-0.57) | 0.62 (0.59-0.65) | |
| ALT | 5 | 0.50 (0.42-0.57) | 0.64 (0.58-0.70) | |
| ALT | 6 | 0.47 (0.36-0.58) | 0.63 (0.56-0.71) | |
| ALT | 7 | 0.41 (0.27-0.55) | 0.70 (0.59-0.80) | |
| ALT | 8 | 0.50 (0.25-0.76) | 0.68 (0.49-0.87) | |
| ALT | 9 | 0.60 (0.44-0.76) | 0.71 (0.60-0.82) | |
| ALT | 10 | 0.51 (0.39-0.62) | 0.64 (0.56-0.72) | |
| AST | 2 | 0.53 (0.50-0.56) | 0.67 (0.64-0.69) | |
| AST | 3 | 0.51 (0.46-0.56) | 0.63 (0.59-0.68) | |
| AST | 4 | 0.47 (0.39-0.56) | 0.62 (0.52-0.71) | |
| AST | 5 | 0.50 (0.32-0.69) | 0.69 (0.53-0.84) | |
| AST | 6 | 0.43 (0.24-0.62) | 0.75 (0.59-0.92) | |
| AST | 7 | 0.05 | 0.90 | |
| AST | 8 | 0.60 | 0.75 | |
| AST | 9 | 0.32 (0.17-0.48) | 0.57 (0.21-0.93) | |
| AST | 10 | 0.69 (0.53-0.85) | 0.53 (0.37-0.68) | |
| Bilirubin | 2 | 0.55 (0.53-0.56) | 0.65 (0.64-0.66) | |
| Bilirubin | 3 | 0.52 (0.50-0.54) | 0.63 (0.61-0.65) | |
| Bilirubin | 4 | 0.50 (0.47-0.54) | 0.62 (0.59-0.65) | |
| Bilirubin | 5 | 0.57 (0.50-0.64) | 0.64 (0.58-0.70) | |
| Bilirubin | 6 | 0.49 (0.38-0.60) | 0.68 (0.61-0.75) | |
| Bilirubin | 7 | 0.43 (0.29-0.58) | 0.62 (0.52-0.73) | |
| Bilirubin | 8 | 0.55 (0.39-0.70) | 0.74 (0.58-0.89) | |
| Bilirubin | 9 | 0.63 (0.47-0.79) | 0.61 (0.49-0.73) | |
| Bilirubin | 10 | 0.61 (0.52-0.70) | 0.66 (0.58-0.73) | |
| RBC | 2 | 0.49 (0.47-0.50) | 0.67 (0.66-0.68) | |
| RBC | 3 | 0.50 (0.48-0.52) | 0.64 (0.62-0.65) | |
| RBC | 4 | 0.49 (0.46-0.52) | 0.63 (0.60-0.65) | |
| RBC | 5 | 0.51 (0.46-0.56) | 0.61 (0.57-0.65) | |
| RBC | 6 | 0.53 (0.46-0.60) | 0.63 (0.58-0.68) | |
| RBC | 7 | 0.50 (0.38-0.61) | 0.65 (0.56-0.74) | |
| RBC | 8 | 0.66 (0.49-0.83) | 0.63 (0.54-0.71) | |
| RBC | 9 | 0.58 (0.41-0.74) | 0.71 (0.59-0.83) | |
| RBC | 10 | 0.62 (0.52-0.71) | 0.67 (0.61-0.73) | |
| Haemoglobin | 2 | 0.57 (0.56-0.58) | 0.69 (0.68-0.70) | |
| Haemoglobin | 3 | 0.56 (0.54-0.58) | 0.66 (0.65-0.68) | |
| Haemoglobin | 4 | 0.57 (0.54-0.61) | 0.64 (0.61-0.66) | |
| Haemoglobin | 5 | 0.58 (0.53-0.62) | 0.64 (0.60-0.67) | |
| Haemoglobin | 6 | 0.63 (0.56-0.69) | 0.66 (0.61-0.71) | |
| Haemoglobin | 7 | 0.55 (0.44-0.66) | 0.63 (0.54-0.72) | |
| Haemoglobin | 8 | 0.51 (0.34-0.69) | 0.64 (0.54-0.74) | |
| Haemoglobin | 9 | 0.59 (0.44-0.73) | 0.65 (0.53-0.78) | |
| Haemoglobin | 10 | 0.60 (0.49-0.70) | 0.68 (0.61-0.74) | |
| Haematocrit | 2 | 0.51 (0.50-0.53) | 0.67 (0.66-0.68) | |
| Haematocrit | 3 | 0.52 (0.50-0.54) | 0.63 (0.62-0.65) | |
| Haematocrit | 4 | 0.52 (0.49-0.55) | 0.63 (0.60-0.65) | |
| Haematocrit | 5 | 0.47 (0.42-0.52) | 0.61 (0.57-0.65) | |
| Haematocrit | 6 | 0.49 (0.41-0.57) | 0.63 (0.58-0.69) | |
| Haematocrit | 7 | 0.49 (0.37-0.60) | 0.64 (0.55-0.72) | |
| Haematocrit | 8 | 0.56 (0.35-0.77) | 0.63 (0.54-0.73) | |
| Haematocrit | 9 | 0.64 (0.48-0.79) | 0.69 (0.58-0.81) | |
| Haematocrit | 10 | 0.43 (0.35-0.51) | 0.67 (0.61-0.73) | |
| MCH | 2 | 0.60 (0.58-0.62) | 0.70 (0.69-0.71) | |
| MCH | 3 | 0.58 (0.56-0.61) | 0.66 (0.64-0.68) | |
| MCH | 4 | 0.57 (0.53-0.60) | 0.67 (0.64-0.69) | |
| MCH | 5 | 0.56 (0.51-0.61) | 0.66 (0.62-0.70) | |
| MCH | 6 | 0.48 (0.39-0.56) | 0.61 (0.55-0.66) | |
| MCH | 7 | 0.53 (0.39-0.67) | 0.67 (0.57-0.77) | |
| MCH | 8 | 0.55 (0.35-0.75) | 0.65 (0.55-0.74) | |
| MCH | 9 | 0.66 (0.48-0.84) | 0.64 (0.49-0.80) | |
| MCH | 10 | 0.53 (0.44-0.62) | 0.64 (0.58-0.70) | |
| MCHC | 2 | 0.54 (0.53-0.56) | 0.67 (0.66-0.68) | |
| MCHC | 3 | 0.56 (0.54-0.58) | 0.63 (0.62-0.65) | |
| MCHC | 4 | 0.52 (0.48-0.55) | 0.62 (0.58-0.65) | |
| MCHC | 5 | 0.57 (0.51-0.62) | 0.62 (0.58-0.67) | |
| MCHC | 6 | 0.56 (0.49-0.64) | 0.64 (0.58-0.70) | |
| MCHC | 7 | 0.64 (0.54-0.74) | 0.64 (0.54-0.74) | |
| MCHC | 8 | 0.47 (0.30-0.65) | 0.64 (0.53-0.74) | |
| MCHC | 9 | 0.50 (0.30-0.70) | 0.73 (0.60-0.86) | |
| MCHC | 10 | 0.59 (0.51-0.68) | 0.66 (0.59-0.72) | |
| MCV | 2 | 0.60 (0.59-0.61) | 0.70 (0.69-0.71) | |
| MCV | 3 | 0.57 (0.55-0.59) | 0.66 (0.65-0.68) | |
| MCV | 4 | 0.56 (0.53-0.60) | 0.66 (0.63-0.69) | |
| MCV | 5 | 0.57 (0.52-0.62) | 0.65 (0.61-0.68) | |
| MCV | 6 | 0.51 (0.43-0.59) | 0.64 (0.58-0.69) | |
| MCV | 7 | 0.62 (0.51-0.74) | 0.68 (0.59-0.76) | |
| MCV | 8 | 0.61 (0.50-0.72) | 0.63 (0.54-0.72) | |
| MCV | 9 | 0.66 (0.49-0.84) | 0.69 (0.54-0.83) | |
| MCV | 10 | 0.48 (0.39-0.56) | 0.62 (0.55-0.68) | |
| RDW | 2 | 0.55 (0.53-0.57) | 0.67 (0.66-0.69) | |
| RDW | 3 | 0.55 (0.52-0.58) | 0.64 (0.61-0.66) | |
| RDW | 4 | 0.57 (0.53-0.62) | 0.62 (0.58-0.65) | |
| RDW | 5 | 0.55 (0.47-0.63) | 0.61 (0.54-0.67) | |
| RDW | 6 | 0.53 (0.41-0.64) | 0.67 (0.59-0.75) | |
| RDW | 7 | 0.44 (0.26-0.63) | 0.71 (0.56-0.86) | |
| RDW | 8 | 0.59 (0.44-0.73) | 0.62 (0.50-0.74) | |
| RDW | 9 | 0.62 (0.31-0.93) | 0.77 (0.64-0.91) | |
| RDW | 10 | 0.57 (0.47-0.68) | 0.60 (0.50-0.69) | |
| Platelets | 2 | 0.60 (0.59-0.62) | 0.71 (0.70-0.72) | |
| Platelets | 3 | 0.60 (0.58-0.62) | 0.68 (0.66-0.69) | |
| Platelets | 4 | 0.55 (0.51-0.59) | 0.65 (0.63-0.68) | |
| Platelets | 5 | 0.58 (0.52-0.63) | 0.65 (0.61-0.69) | |
| Platelets | 6 | 0.56 (0.46-0.66) | 0.66 (0.60-0.72) | |
| Platelets | 7 | 0.60 (0.48-0.73) | 0.65 (0.55-0.75) | |
| Platelets | 8 | 0.64 (0.50-0.78) | 0.71 (0.60-0.82) | |
| Platelets | 9 | 0.50 (0.22-0.79) | 0.57 (0.43-0.71) | |
| Platelets | 10 | 0.49 (0.38-0.59) | 0.62 (0.55-0.69) | |
| MPV | 2 | 0.54 (0.52-0.57) | 0.70 (0.67-0.72) | |
| MPV | 3 | 0.51 (0.46-0.56) | 0.63 (0.58-0.67) | |
| MPV | 4 | 0.58 (0.51-0.65) | 0.64 (0.58-0.70) | |
| MPV | 5 | 0.56 (0.45-0.67) | 0.51 (0.37-0.64) | |
| MPV | 6 | 0.59 (0.44-0.75) | 0.71 (0.57-0.86) | |
| MPV | 7 | 0.15 (0.06-0.24) | 0.12 (0.04-0.19) | |
| MPV | 8 | 0.98 | 0.96 | |
| MPV | 9 | 0.58 (0.20-0.96) | 0.86 (0.63-1.00) | |
| MPV | 10 | 0.58 (0.38-0.79) | 0.61 (0.39-0.83) | |
| WBC | 2 | 0.62 (0.60-0.63) | 0.71 (0.70-0.72) | |
| WBC | 3 | 0.59 (0.57-0.61) | 0.67 (0.65-0.68) | |
| WBC | 4 | 0.58 (0.54-0.61) | 0.65 (0.63-0.68) | |
| WBC | 5 | 0.60 (0.54-0.65) | 0.64 (0.60-0.68) | |
| WBC | 6 | 0.58 (0.50-0.66) | 0.65 (0.59-0.71) | |
| WBC | 7 | 0.57 (0.45-0.69) | 0.64 (0.54-0.74) | |
| WBC | 8 | 0.52 (0.37-0.67) | 0.59 (0.49-0.70) | |
| WBC | 9 | 0.37 (0.19-0.55) | 0.59 (0.45-0.72) | |
| WBC | 10 | 0.51 (0.42-0.59) | 0.59 (0.52-0.66) | |
| Basophils | 2 | 0.52 (0.50-0.53) | 0.66 (0.65-0.67) | |
| Basophils | 3 | 0.49 (0.46-0.51) | 0.64 (0.62-0.66) | |
| Basophils | 4 | 0.51 (0.47-0.54) | 0.62 (0.59-0.65) | |
| Basophils | 5 | 0.55 (0.50-0.61) | 0.61 (0.57-0.66) | |
| Basophils | 6 | 0.54 (0.46-0.62) | 0.67 (0.61-0.72) | |
| Basophils | 7 | 0.57 (0.43-0.70) | 0.63 (0.54-0.72) | |
| Basophils | 8 | 0.31 (0.18-0.45) | 0.66 (0.56-0.76) | |
| Basophils | 9 | 0.48 (0.30-0.66) | 0.68 (0.56-0.81) | |
| Basophils | 10 | 0.40 (0.30-0.50) | 0.66 (0.60-0.72) | |
| Eosinophils | 2 | 0.52 (0.50-0.54) | 0.67 (0.65-0.68) | |
| Eosinophils | 3 | 0.49 (0.46-0.52) | 0.64 (0.62-0.66) | |
| Eosinophils | 4 | 0.51 (0.46-0.55) | 0.62 (0.60-0.65) | |
| Eosinophils | 5 | 0.43 (0.37-0.50) | 0.62 (0.58-0.65) | |
| Eosinophils | 6 | 0.51 (0.38-0.64) | 0.64 (0.59-0.70) | |
| Eosinophils | 7 | 0.45 (0.31-0.60) | 0.62 (0.53-0.71) | |
| Eosinophils | 8 | 0.32 (0.09-0.55) | 0.65 (0.56-0.74) | |
| Eosinophils | 9 | 0.75 (0.56-0.95) | 0.71 (0.60-0.83) | |
| Eosinophils | 10 | 0.37 (0.26-0.47) | 0.67 (0.61-0.73) | |
| Lymphocytes | 2 | 0.51 (0.50-0.53) | 0.67 (0.66-0.68) | |
| Lymphocytes | 3 | 0.50 (0.48-0.52) | 0.64 (0.62-0.65) | |
| Lymphocytes | 4 | 0.51 (0.47-0.54) | 0.63 (0.60-0.65) | |
| Lymphocytes | 5 | 0.48 (0.43-0.53) | 0.62 (0.58-0.66) | |
| Lymphocytes | 6 | 0.53 (0.45-0.61) | 0.64 (0.58-0.69) | |
| Lymphocytes | 7 | 0.48 (0.36-0.60) | 0.63 (0.55-0.71) | |
| Lymphocytes | 8 | 0.36 (0.20-0.52) | 0.69 (0.59-0.79) | |
| Lymphocytes | 9 | 0.39 (0.20-0.58) | 0.66 (0.55-0.77) | |
| Lymphocytes | 10 | 0.53 (0.44-0.62) | 0.66 (0.59-0.72) | |
| Monocytes | 2 | 0.59 (0.57-0.60) | 0.70 (0.69-0.71) | |
| Monocytes | 3 | 0.58 (0.56-0.60) | 0.66 (0.64-0.68) | |
| Monocytes | 4 | 0.57 (0.54-0.61) | 0.65 (0.62-0.67) | |
| Monocytes | 5 | 0.53 (0.48-0.59) | 0.62 (0.58-0.66) | |
| Monocytes | 6 | 0.50 (0.43-0.58) | 0.63 (0.56-0.69) | |
| Monocytes | 7 | 0.62 (0.50-0.74) | 0.65 (0.55-0.75) | |
| Monocytes | 8 | 0.37 (0.19-0.54) | 0.59 (0.47-0.70) | |
| Monocytes | 9 | 0.29 (0.13-0.45) | 0.61 (0.49-0.73) | |
| Monocytes | 10 | 0.55 (0.46-0.65) | 0.63 (0.56-0.70) | |
| Neutrophils | 2 | 0.63 (0.61-0.64) | 0.71 (0.70-0.72) | |
| Neutrophils | 3 | 0.58 (0.56-0.61) | 0.67 (0.65-0.68) | |
| Neutrophils | 4 | 0.58 (0.54-0.61) | 0.66 (0.63-0.68) | |
| Neutrophils | 5 | 0.62 (0.57-0.67) | 0.64 (0.60-0.68) | |
| Neutrophils | 6 | 0.53 (0.46-0.60) | 0.61 (0.55-0.67) | |
| Neutrophils | 7 | 0.57 (0.46-0.69) | 0.63 (0.54-0.73) | |
| Neutrophils | 8 | 0.50 (0.29-0.71) | 0.62 (0.51-0.74) | |
| Neutrophils | 9 | 0.59 (0.41-0.76) | 0.69 (0.56-0.82) | |
| Neutrophils | 10 | 0.42 (0.34-0.50) | 0.60 (0.53-0.66) | |
| CRP | 2 | 0.63 (0.61-0.66) | 0.72 (0.70-0.74) | |
| CRP | 3 | 0.62 (0.58-0.66) | 0.69 (0.66-0.73) | |
| CRP | 4 | 0.66 (0.60-0.73) | 0.65 (0.57-0.73) | |
| CRP | 5 | 0.63 (0.51-0.74) | 0.65 (0.55-0.76) | |
| CRP | 6 | 0.41 (0.25-0.57) | 0.68 (0.54-0.82) | |
| CRP | 7 | 0.54 (0.18-0.91) | 0.59 (0.27-0.91) | |
| CRP | 8 | 0.68 (0.39-0.98) | 0.82 (0.64-1.00) | |
| CRP | 9 | 0.60 (0.43-0.77) | 0.76 (0.61-0.91) | |
| CRP | 10 | 0.56 (0.43-0.69) | 0.62 (0.48-0.75) | |
| ESR | 2 | 0.64 (0.61-0.66) | 0.72 (0.70-0.74) | |
| ESR | 3 | 0.62 (0.58-0.66) | 0.69 (0.66-0.73) | |
| ESR | 4 | 0.66 (0.60-0.73) | 0.65 (0.57-0.73) | |
| ESR | 5 | 0.62 (0.51-0.74) | 0.65 (0.55-0.75) | |
| ESR | 6 | 0.41 (0.25-0.57) | 0.68 (0.54-0.82) | |
| ESR | 7 | 0.54 (0.17-0.91) | 0.59 (0.27-0.90) | |
| ESR | 8 | 0.68 (0.39-0.97) | 0.82 (0.64-1.00) | |
| ESR | 9 | 0.60 (0.43-0.77) | 0.76 (0.61-0.91) | |
| ESR | 10 | 0.56 (0.43-0.69) | 0.62 (0.48-0.75) | |
| PV | 2 | 0.65 (0.60-0.70) | 0.72 (0.67-0.77) | |
| PV | 3 | 0.72 (0.61-0.83) | 0.64 (0.52-0.76) | |
| PV | 4 | 0.57 (0.37-0.78) | 0.62 (0.43-0.82) | |
| PV | 5 | 0.80 |  | |
| PV | 6 | 0.87 | 0.87 | |
| PV | 7 |  |  | |
| PV | 8 | 0.77 (0.31-1.00) | 0.95 (0.85-1.00) | |
| PV | 9 |  |  | |
| PV | 10 | 0.57 (0.36-0.79) | 0.72 (0.47-0.98) | |
| NLR | 2 | 0.58 (0.57-0.60) | 0.70 (0.69-0.71) | |
| NLR | 3 | 0.57 (0.54-0.59) | 0.66 (0.64-0.68) | |
| NLR | 4 | 0.54 (0.50-0.57) | 0.64 (0.61-0.66) | |
| NLR | 5 | 0.55 (0.50-0.60) | 0.63 (0.59-0.67) | |
| NLR | 6 | 0.45 (0.37-0.53) | 0.60 (0.54-0.65) | |
| NLR | 7 | 0.58 (0.46-0.70) | 0.65 (0.57-0.74) | |
| NLR | 8 | 0.59 (0.41-0.76) | 0.71 (0.58-0.83) | |
| NLR | 9 | 0.76 (0.65-0.87) | 0.70 (0.58-0.82) | |
| NLR | 10 | 0.46 (0.37-0.55) | 0.62 (0.55-0.68) | |
| PLR | 2 | 0.56 (0.54-0.59) | 0.69 (0.68-0.70) | |
| PLR | 3 | 0.57 (0.53-0.61) | 0.67 (0.65-0.68) | |
| PLR | 4 | 0.58 (0.52-0.65) | 0.65 (0.62-0.68) | |
| PLR | 5 | 0.61 (0.52-0.70) | 0.66 (0.62-0.70) | |
| PLR | 6 | 0.49 (0.30-0.68) | 0.64 (0.58-0.70) | |
| PLR | 7 | 0.44 (0.20-0.68) | 0.62 (0.52-0.72) | |
| PLR | 8 | 0.63 (0.40-0.87) | 0.77 (0.66-0.88) | |
| PLR | 9 | 0.27 (0.03-0.50) | 0.65 (0.53-0.77) | |
| PLR | 10 | 0.52 (0.33-0.71) | 0.64 (0.57-0.71) | |
| mGPR | 2 | 0.62 (0.60-0.65) | 0.71 (0.69-0.74) | |
| mGPR | 3 | 0.65 (0.59-0.70) | 0.66 (0.59-0.72) | |
| mGPR | 4 | 0.57 (0.46-0.67) | 0.56 (0.43-0.69) | |
| mGPR | 5 | 0.58 (0.40-0.76) | 0.60 (0.43-0.78) | |
| mGPR | 6 | 0.51 (0.28-0.74) | 0.66 (0.47-0.86) | |
| mGPR | 7 | 0.58 (0.24-0.92) | 0.81 (0.61-1.00) | |
| mGPR | 8 | 0.85 (0.70-1.00) | 0.90 (0.78-1.00) | |
| mGPR | 9 | 0.47 (0.25-0.69) | 0.68 (0.47-0.89) | |
| mGPR | 10 | 0.52 (0.37-0.67) | 0.56 (0.41-0.71) | |
| This table shows the AUC of unadjusted and age- and sex-adjusted one-year trend for cancer in six months following UWL in subgroups defined using the number of repeat tests per patient used to derive the trend. For some subgroups, the CIs are missing because there were insufficient events to estimate a standard error and therefore the CI – these AUCs in particular should be interpreted with caution  Abbreviations: UWL=unexpected weight loss: ALP=alkaline phosphatase; ALT=alanine aminotransferase; AST=aspartate aminotransferase; RBC=red blood cell count; MCH=mean cell haemoglobin; MCHC=mean cell haemoglobin concentration; MCV=mean cell volume; RDW=red cell distribution width; MPV=mean platelet volume; WBC=white blood cell count; PV=plasma viscosity; CRP=C-reactive protein; ESR=erythrocyte sedimentation rate; NLR=neutrophil-lymphocyte ratio; PLR=platelet-lymphocyte ratio; mGPR=modified Glasgow Prognostic ratio. | | | |  |
