# Supplementary material for: Blood test trend versus single threshold abnormality to discriminate cancer from non-cancer in patients with unexpected weight loss: A retrospective cohort study
Source: PLoS Med. 2026 Sep 17;23(9):e1004956. doi: 10.1371/journal.pmed.1004956 (PMC13585255; doi:10.1371/journal.pmed.1004956)
Supplement: Supplementary file 4 [file pmed.1004956.s006.docx]

| **No.** | **Reviewer comment** | **Author response** | **Changes made** |
| --- | --- | --- | --- |
|  | **Editor** |  |  |
| 1 | We ask every co-author listed on the manuscript to fill in a contributing author statement, making sure to declare all competing interests. If any of the co-authors have not filled in the statement, we will remind them to do so when the paper is revised. If all statements are not completed in a timely fashion this could hold up the re-review process. If new competing interests are declared later in the revision process, this may also hold up the submission. Should there be a problem getting one of your co-authors to fill in a statement we will be in contact. Please do not add or remove authors without first discussing this with the handling editor. You can see our competing interests policy here: <http://journals.plos.org/plosmedicine/s/competing-interests>. | Thanks for your comments. We have included a “Competing interests” section near the end of the manuscript and will encourage co-authors to complete the contributing author statement once you have sent the notification to each co-author. | Not applicable. |
| 2 | Please ensure that the funders and grant numbers match between the Financial Disclosure field and the Funding Information tab in your submission form. Note that the funders must be provided in the same order in both places as well.  - State the initials, alongside each funding source, of each author to receive each grant. - State what role the funders took in the study. If the funders had no role in your study, please state: “The funders had no role in study design, data collection and analysis, decision to publish, or preparation of the manuscript.” | Apologies that the funding details were inconsistent. We have now corrected the funding details on the submission system to match those in the manuscript, including the order. We have also added the initials of the author who received each grant in the manuscript. We have also added that the funders had no role in the study. | *Funding:*  “BDN and PSV were funded by the NIHR Policy Research Programme (Policy Research Unit on Cancer Awareness, Screening and Early Diagnosis, reference PR-PRU-NIHR206132) and a Cancer Research UK Fellowship (RCCPDF\100005). The funders had no role in study design, data collection and analysis, decision to publish, or preparation of the manuscript. The views expressed are those of the authors and not necessarily those of Cancer Research UK, the NIHR, or the Department of Health and Social Care.” |
| 3 | In your Data Availability Statement, please ensure you have provided all necessary access information in complete sentences. Access information should include the name of any data repositories used as well as digital object identifiers (DOIs), accession numbers or codes, or other persistent identifiers for all relevant data. If all data has been provided within your submission files, please use the statement "All relevant data is available within the manuscript and Supporting Information files." The Data Availability Statement will appear on the published version of your manuscript. | We have now added a Data Availability Statement after the Funding section and included the necessary information, as requested. | *Data Availability Statement:*  “The data used in this study is available from the Clinical Practice Research Datalink, but restrictions apply – the CPRD are required to approve data access.” |
| 4 | Please provide separate figure files in .tif or .eps format. For more information about figure files please see our guidelines:  <https://journals.plos.org/plosmedicine/s/figures> | We have now uploaded figures as separate .tif files. | Not applicable. |
| 5 | We have noticed that you have uploaded Supporting Information files, but you have not included a list of legends. Please add a full list of legends for your Supporting Information files after the references list. | Apologies for omitting this. We have now added a list of legends for the information in the Supporting Information file after the References. | *Supporting information file legends:*  “Table S1: Summary of blood test abnormality  Table S2: Sample size calculation  Table S3: Number of patients with repeat blood tests over each longitudinal window before UWL (n=275,205)…” |
| 6 | We expect all researchers with submissions to PLOS in which author-generated code underpins the findings in the manuscript to make all author-generated code available without restrictions upon publication of the work. In cases where code is central to the manuscript, we may require the code to be made available as a condition of publication. Authors are responsible for ensuring that the code is reusable and well documented. Please make any custom code available, either as part of your data deposition or as a supplementary file. Please add a sentence to your data availability statement regarding any code used in the study, e.g. "The code used in the analysis is available from Github [URL] and archived in Zenodo [DOI link]" Please review our guidelines at https://journals.plos.org/plosmedicine/s/materials-software-and-code-sharing and ensure that your code is shared in a way that follows best practice and facilitates reproducibility and reuse. Because Github depositions can  be readily changed or deleted, we encourage you to make a permanent DOI'd copy (e.g. in Zenodo) and provide the URL | We will add the R code we used to perform the data analysis to the GitHub location referenced in the manuscript once the revisions process has completed (as the code will likely change if further revisions are requested). We have also added the requested information in the data availability statement. | *Data Availability Statement*  "The R code used in the analysis is available from Github[14]" |
| 7 | Abstract: Please structure your abstract using the PLOS Medicine headings (Background, Methods and Findings, Conclusions). Please combine the Methods and Findings sections into one section | We have updated, as requested. | Abstract: Methods and Results section now combined. “Discussion” now changed to “Conclusion”. |
| 8 | At this stage, we ask that you include a short, non-technical Author Summary of your research to make findings accessible to a wide audience that includes both scientists and non-scientists. The Author Summary should immediately follow the Abstract in your revised manuscript. This text is subject to editorial change and should be distinct from the scientific abstract. Ideally each sub-heading should contain 2-3 single sentence, concise bullet points containing the most salient points from your study. In the final bullet point of 'What Do These Findings Mean?', please include the main limitations of the study in non-technical language. Please see our author guidelines for more information: <https://journals.plos.org/plosmedicine/s/revising-your-manuscript#loc-author-summary>. | We have now added an Author Summary section, as requested. | *Author summary:*  Why Was This Study Done?   - Abnormal blood test results, such as low haemoglobin or raised platelets, support cancer risk assessment in primary care. - Monitoring trends (changes over time) in blood test results could enhance cancer risk assessment. - No study has assessed the potential of blood test trends in patients who present with non-specific symptoms, such as unexpected weight loss (UWL).   What Did the Researchers Do and Find?   - We conducted a cohort study using electronic health records data for 275,205 patients with UWL attending English primary care. - We compared the discrimination of trends in 26 blood tests prior to UWL for a new cancer diagnosis with the abnormality for the same blood test that is used in clinical practice. - We found that age- and sex-adjusted blood test abnormalities and trends were more discriminative than unadjusted blood test trends and abnormalities, and for 40 test-cancer combinations, adjusted trends were more discriminative than the equivalent adjusted blood test abnormality, including 33 trends for cancer overall, mean cell volume trend for bowel cancer and lymphoma, white blood count and neutrophil trend for lung cancer, and aspartate aminotransferase, red blood cell count, haematocrit, and platelet-to-lymphocyte ratio trend for prostate cancer.   What Do These Findings Mean?   - Blood test results should be adjusted by age and sex to improve patient selection for cancer investigation in patients attending primary care with UWL. - Blood test trends over repeat tests could further enhance patient selection in some test-cancer combinations. - Our analysis uses routinely collected data so is limited to when patients had blood tests in clinical practice and to patients who had blood tests. |
| 9 | Please express the main results with 95% CIs as well as p values. When reporting p values please report as p<0.001 and where higher as the exact p value p=0.002, for example. Throughout, suggest reporting statistical information as follows to improve clarity for the reader "22% (95% CI [13%,28%]; p</=)". Please be sure to define all numerical values at first use. | We confirm that we have not reported p-values in the manuscript, only confidence intervals are presented, where applicable. We have also defined numerical values at first use. | Not applicable. |
| 10 | Please include page numbers and line numbers in the manuscript file. Use continuous line numbers (do not restart the numbering on each page). | We confirm that page numbers and continuous line numbers are used. | Not applicable. |
| 11 | Please cite the reference numbers in square brackets. Citations should precede punctuation | We confirm that square brackets have been used and that these precede punctuation. | Not applicable. |
| 12 | Please provide titles and legends for all figures and tables (including those in Supporting Information files). | We confirm that titles and, where applicable, legends are provided for each figure and table, including those in the supplementary file. We have added greater detail to the legends to make the tables and figures more standalone. | We have added greater detail to the legends for all figures and tables. |
| 13 | Please define all abbreviations used in each figure/table (including those in Supporting Information files). | Apologies for missing this. We have now added full spellings for abbreviations in tables and figures in the manuscript and supplementary file. | All abbreviations in tables and figures are now spelled out in full in below the table/figure. |
| 14 | Please consider avoiding the use of red and green in order to make your figure more accessible to those with color blindness. | We confirm that we have not used red and green in figures. | Not applicable. |
| 15 | Please note that supplementary material will be posted as supplied by the authors. Therefore, please amend it according to the relevant comments outlined here.  Please cite your Supporting Information as outlined here: <https://journals.plos.org/plosmedicine/s/supporting-information> | We confirm that each supplementary table and figure has been cited in the manuscript using the format Table S# and Figure S#, respectively. | Not applicable. |
| 16 | PLOS uses the numbered citation (citation-sequence) method and first six authors, et al | We confirm that we have used the numbered citation format and that at least the first six authors have been written. | Not applicable. |
| 17 | Please ensure that journal name abbreviations match those found in the National Center for Biotechnology Information (NCBI) databases (<http://www.ncbi.nlm.nih.gov/nlmcatalog/journals>), and are appropriately formatted and capitalised. | Journal names are now spelled out in full to ensure they match. | Journal names amended from abbreviated versions to fully spelled out versions. |
| 18 | Where website addresses are cited, please include the complete URL and specify the date of access (e.g. [accessed: 12/06/2023]). | We confirm that website citations include the complete URL and date of access in the reference list. | Not applicable. |
| 19 | Abstract: Please include the study design, population and setting, number of participants, years during which the study took place (enrollment and follow up), length of follow up, and main outcome measures. | We have now added the length of follow-up to the abstract and confirm that the other details are present. | *Abstract, Methods and findings*:  “The median (IQR) time between the first and last blood test per person was 5.2 (1.5-8.2) years cases and 4.3 (1.0-7.7) years for those cancer-free. The median (IQR) time between the last blood test and UWL per person was 0.1 (0.0-0.6) years for cases and 0.3 (0.0-1.1) years for those cancer-free.” |
| 20 | Please ensure that the study is reported according to the RECORD guideline (available from <https://www.record-statement.org)> and include the completed checklist as Supporting Information. Please add the following statement, or similar, to the Methods: "This study is reported as per the Reporting of Studies Conducted using Observational Routinely-Collected Data (RECORD) guideline (S1 Checklist)." When completing the checklist, please use section and paragraph numbers, rather than page numbers. | We have now included a combined STROBE and RECORD checklist in the submission. | RECORD checklist now included in the submission with the STROBE checklist.  *Methods, first paragraph*:  “Study reporting follows the STROBE, RECORD, and STARD guidelines. Populated checklists for each are in the Supplementary files.” |
| 21 | In the manuscript text, please indicate: (1) the specific hypotheses you intended to test, (2) the analytical methods by which you planned to test them, (3) the analyses you actually performed, and (4) when reported analyses differ from those that were planned, transparent explanations for differences that affect the reliability of the study's results. If a reported analysis was performed based on an interesting but unanticipated pattern in the data, please be clear that the analysis was data driven. | The manuscript describes our goals at the end of the introduction and analytic methods. Analyses were performed as planned. | Not applicable. |
| 22 | Please state in the Methods section whether the study had a prospective protocol or analysis plan. If a prospective analysis plan (from your funding proposal, IRB or other ethics committee submission, study protocol, or other planning document written before analyzing the data) was used in designing the study, please include the relevant document(s) with your revised manuscript as a Supporting Information file to be published alongside your study and cite it in the Methods section. A legend for this file should be included at the end of your manuscript. If no such document exists, please make sure that the Methods section transparently describes when analyses were planned, and when/why any data-driven changes to analyses took place. Changes in the analysis, including those made in response to peer review comments, should be identified as such in the Methods section of the paper, with rationale. | We did not have a prospective protocol or analysis plan. However, analyses were discussed and agreed a-priori, but not documented in a formal analysis plan. We have made this clearer in the Methods. | *Methods, Analysis, paragraph 1*:  “Analyses were planned a-priori and…” |
| 23 | Please ensure that the study is reported according to the STARD guideline (<https://www.equator-network.org/reporting-guidelines/stard/>) and include the completed STARD checklist as Supporting Information. Please add the following statement, or similar, to the Methods: "This study is reported as per the Standards for Reporting of Diagnostic Accuracy (STARD) guideline (S1 Checklist)." When completing the checklist, please use section and paragraph numbers, rather than page numbers. | We have now included a STARD checklist in the submission. | STARD checklist now included in the submission with the STROBE checklist.  *Methods, first paragraph*:  “Study reporting follows the STROBE, RECORD, and STARD guidelines. Populated checklists for each are in the Supplementary files.” |
| 24 | Please structure your Abstract according to STARD for Abstracts (<https://www.equator-network.org/reporting-guidelines/stard-abstracts/>). | We confirm that the applicable information in the checklist is reported in the Abstract. However, the suggested restructure in this comment is inconsistent with the suggested structure suggested in your comment number 6. We have left the Abstract structured as requested in your comment number 6. | Not applicable. |
| 25 | Please structure the Methods section using the following sub-headings: Study design, Participants, Test methods, Analysis. | We have updated the Methods section to follow these sub-headings | Methods section – headings updated |
| 26 | Please include a diagram to describe the flow of participants through the study (typically figure 1). | We confirm a flow diagram is reported in Figure 1 | Not applicable. |
|  |  |  |  |
|  | **Reviewer 1** |  |  |
| 1 | The primary estimand is not clearly defined. It remains ambiguous whether the objective is etiological (association between blood test trend and cancer diagnosis) or predictive/diagnostic (risk stratification and discrimination). The manuscript interchanges hazard ratios and AUC metrics without clearly specifying whether the modelling framework is intended for risk prediction or hypothesis testing. | Thanks for your comments. Apologies it was unclear – we can confirm that the study objective was to assess the role of blood test trend to inform cancer diagnosis. This was not a risk prediction modelling study. The HRs were included to report the effect size of trend and abnormality and the AUCs were reported as the high-level summary metric. We have made the objective clearer in the Abstract and in the Introduction. | *Introduction, last paragraph:*  Our aim was to explore the discrimination of blood test trends for a new diagnosis of cancer compared to the equivalent blood test abnormality in patients presenting with UWL to primary care, by cancer type and time period. |
| 2 | The manuscript does not report any evaluation of the proportional hazards assumption (e.g., Schoenfeld residuals), which is essential when Cox modelling is used. | We agree it is important to check the PH assumption. We have now made it clearer that we checked the PH assumption using plots of Schoenfeld residuals for every blood test in the adjusted 1-year trend models and abnormality models – we did not consider any model to violate to the PH assumption. | *Methods, Analysis, fourth paragraph:*  “For each blood test in the 1-year trend models and abnormality models, we examined the proportional hazards assumption using plots of Schoenfeld residuals – no model violated the proportional hazards assumption (plots are available from the authors).” |
| 3 | The use of Cox proportional hazards models with a fixed six-month follow-up window requires stronger justification. Given the short and bounded follow-up period, logistic regression could provide equivalent discrimination assessment without reliance on proportional hazards assumptions. | We agree that with a short outcome window, logistic regression could be more favourable than a time-to-event model. However, joint modelling traditionally requires the use of a time-to-event sub-model to make predictions – other sub-models, such as logistic, cannot easily replace the Cox model in the joint modelling framework. Two-stage models exist, such as Landmarking, where, unlike with joint modelling, the mixed-effects model is derived independently of the Cox model, so a logistic model could possibly be used instead of the Cox model. However, two-stage approaches are known to under-estimate the standard errors, as they fail to account for the uncertainty associated with estimating the longitudinal biomarker trajectory. We therefore opted to use established and easily accessible approaches in the joint modelling framework, i.e. Cox models. | Not applicable. |
| 4 | The handling of time origin and potential immortal time bias requires clarification. Patients were required to have at least one blood test within 10 years prior to UWL and, for trend analyses, at least two tests including one near UWL. This selection may introduce conditioning on future information relative to earlier time points. The temporal structure should be more clearly articulated to ensure no forward-looking bias is introduced into the risk models. | We can confirm that the index date (entry into the cohort and start of the 6m risk window) is the date of UWL. All predictors are derived from historic data prior to UWL. The 2+ test requirement is a data availability constraint for estimating trends and we agree it conditions on having sufficient longitudinal data, which could introduce selection bias toward patients with more frequent testing prior to UWL. However, our analysis was not intended to represent a prospective risk prediction setting – results from this cohort are interpreted as an assessment of trends among patients with repeat testing, not necessarily generalisable to all patients with blood tests. As future work, we intend to derive a prediction model and this would include all tested patients with UWL, not just those with 2+ tests. | *Discussion, Strengths and limitations, second paragraph:*  “Our trend analysis was also restricted to patients with 2 or more tests in each trend window. This could introduce selection bias, as patients with multiple blood tests must be alive for both tests and may have utilised healthcare services compared to those with one blood test. Patients with 2 or more tests are potentially therefore more likely to be diagnosed with cancer in primary care. However, when conducting our data checks, analyses showed that the cancer incidence in patients with only one test was comparable to patients with two or more (results are available from the authors). In future work, we intend to derive a prediction model and this would include all tested patients with UWL, not just those with 2 or more tests.” |
| 5 | The joint modelling specification requires more detail and justification. Although mixed-effects models with random intercept and slope are described, the functional form of the association between the longitudinal process and the survival outcome is not explicitly stated (e.g., current value parameterisation, slope parameterisation, shared random effects). The exact structure linking the longitudinal sub-model to the Cox model should be clarified. | We have now explicitly stated that this modelling framework uses latent shared random effects linking the longitudinal and time-to-event models. The manuscript also includes references that further explain the joint modelling framework (reference 17 and 18). | *Methods, Analysis, second paragraph:*  “The linked Cox model then included the blood test trend (as latent shared random effects from the mixed-effects component) as a predictor for cancer diagnosis” |
| 6 | The use of piecewise linear splines for time-to-UWL and age-at-UWL requires methodological justification. The rationale for knot placement at 6 months and 1, 2, and 3 years is not provided. It is unclear whether alternative knot configurations were explored or whether model fit diagnostics were conducted. | We can confirm that we explored non-linearity by fitting models that included either fractional polynomials, linear splines, or restricted cubic splines, with varying knot locations for each blood test for the latter two. We compared the AIC and BIC of each model and found that linear splines with the chosen knot locations often resulted in better fitting models than fractional polynomials and restricted cubic splines and splines with other knot locations. For consistency, we therefore opted for linear splines and these knot locations for all models. We have now made this clearer in the manuscript. | *Methods, Analysis, second paragraph:*  “Linear splines and knot locations were chosen based on visual inspection of LOWESS plots and comparison of AIC and BIC of models using fractional polynomials, restricted cubic splines, linear splines, and different knot locations.” |
| 7 | The decision to model each blood test separately limits interpretability and may inflate Type I error due to extensive multiplicity. Twenty-six blood tests across multiple time windows and cancer subtypes generate a large number of comparisons. No correction for multiple testing or false discovery control is reported. | You are correct that we did not report results adjusted for multiple testing. This is firstly because this study is hypothesis-generating, building a case for a future prediction model. Secondly, because we are not performing any hypothesis tests, so we did not consider or report p-values. We did however consider adjusting the 95% CIs to 99.952% CIs (based on the Bonferroni approach: 26 tests times 4 trend windows gives 104 tests, then a 5% significance level divided by 104 gives a 0.048% significance level). But given the very large sample and number of events, shifting from 95% to 99.952% limits only changed the CIs at the 3^rd^ or 4^th^ decimal point and beyond. So, we kept with 95% CIs, as these are easier for readers to comprehend. We have now added these details to the Strengths and limitations section. | *Discussion, Strengths and limitations, first paragraph:*  “This was further demonstrated when we assessed the influence of multiple testing on our findings: we adjusted the 95% CIs to 99.952% CIs, based on a Bonferroni adjustment of a 5% significance level to 0.048% due to 104 test and trend window combinations, but found that the CIs only changed from the 3^rd^ or 4^th^ decimal point onwards. We therefore retained 95% CIs.” |
| 8 | Discrimination assessment using AUC derived from Cox models requires clarification. It is not specified whether time-dependent ROC curves were used or whether a fixed-time AUC at six months was estimated. Standard AUC methods are not directly applicable to censored survival data without appropriate modification. | We can confirm that we used a fixed-time AUC at six months following UWL. We agree that standard methods are not directly applicable to Cox models, but we chose the AUC because this is a high-level summary metric and offers a sense of discrimination, which was our main goal. For a future predictive model, we will include further metrics, such as Somer’s D. | *Methods, Analysis, fifth paragraph:*  “Performance of trend and abnormality was assessed using the fixed-time area under the curve (AUC) at six months following UWL as a high-level summary metric of discrimination across multiple comparisons.” |
| 9 | No internal validation strategy is described. Given the extensive modelling and comparison of trends versus abnormalities, overfitting is a concern. There is no mention of bootstrapping, cross-validation, shrinkage, or optimism correction. | Apologies for the confusion, but we did not derive a predictive model, so internal validation was not considered, so no shrinkage was assessed and overfitting corrected for. We agree that the AUC from our analyses could still be subject to optimism, but our sample size was sufficiently large to ensure a well-powered analysis and reduces the potential optimism – as per your later comment, we have performed sample size calculations to make this clearer. | Not applicable. |
| 10 | Missing data handling is insufficiently described. Several baseline covariates (e.g., ethnicity, BMI, smoking) show substantial missingness. It is unclear whether these were excluded from modelling, included as missing categories, or addressed using multiple imputation. Even if not included in final models, their exclusion requires justification. | The only variables considered for modelling were the trend/abnormality, age, and sex – these were complete for all patients, so the modelling was not subject to missing data. Only age and sex were considered because these are easily accessible in primary care. The other variables, such as ethnicity and BMI, were only ever considered to describe the sample, with patients with missing data included in a “Missing” category in Table 1. | *Methods, Analysis, third paragraph:*  “The multivariable analysis subsequently adjusted cancer risk for age at UWL (years) and sex, as these are easily accessible in primary care systems. Age and sex were available for all patients. Other data, such as ethnicity and BMI, were considered only to describe the sample, with patients with missing data included in a “Missing” category.” |
| 11 | The requirement of at least two blood tests within a given window introduces selection bias toward patients with greater healthcare utilisation. This may induce collider bias if healthcare contact is associated with both blood testing frequency and cancer risk. The implications of restricting to 2+ tests for trend modelling should be discussed and sensitivity analyses considered. | We agree that the requirement of two or more tests could represent a group of patients with greater healthcare utilisation, especially as the trend-window gets narrower. We made this requirement because we wanted to explore the role of trend and people need at least two tests for a trend to be calculated. We also found that, for each test separately, the cancer incidence in patients with only one test was comparable to patients with two or more. For a future predictive model, to reduce collider/selection bias, we would not make a requirement of two or more tests and instead include patients with at least one test (it would consider the test value and/or the trend, so would work if a patient has only one test or multiple). A future predictive model would also include, for example, co-morbidities as covariates, to adjust for possible explanations for greater healthcare utilisation in some patients. We have now added a limitation on the potential for selection bias. | *Discussion, Strengths and limitations, second paragraph:*  “Our trend analysis was also restricted to patients with 2 or more tests in each trend window. This could introduce selection bias, as patients with multiple blood tests must be alive for both tests and may have utilised healthcare services compared to those with one blood test. Patients with 2 or more tests are potentially therefore more likely to be diagnosed with cancer in primary care. However, when conducting our data checks, analyses showed that the cancer incidence in patients with only one test was comparable to patients with two or more (results are available from the authors). In future work, we intend to derive a prediction model and this would include all tested patients with UWL, not just those with 2 or more tests” |
| 12 | The comparison between "trend" and "abnormality" may not be methodologically equivalent. The abnormality model uses a binary threshold, whereas the trend model uses continuous longitudinal modelling with random effects and spline functions. This structural difference may inherently favour trend-based modelling due to greater modelling flexibility. A fairer comparison would require aligning model complexity. | We agree that, theoretically, it is possible for the trend models to be favoured, as they offer more flexibility. However, in our study, we mostly see no difference in the (age- and sex-adjusted) discrimination of trend vs abnormality, suggesting this isn’t the case. We started with a binary threshold because this is what is used in clinical practice. | Not applicable. |
| 13 | Effect sizes for trends (e.g., HRs close to 1.00-1.08) suggest very small per-unit effects. Interpretation of the unit of change (per SD? per raw unit? per year?) is not sufficiently clear. Clinical interpretability of these HRs requires more explicit scaling. | You are correct that the HRs for trend suggest small effects. This was expected before we derived the models, as the LOWESS plots indicate small average-level differences in the blood test trend between patients with and without cancer. Apologies it was unclear how to interpret these HRs: they reflect how a one unit change in the patient-specific slope increases the outcome. For example, a 1 g/L per year decline in the patient-specific trend for albumin increases the hazard of cancer. We have added a footnote in Table 3 to explain this. We have also added Table S4, which shows HRs that have been rescaled to more clinically meaningful per-unit changes. Specifically, we rescaled to show 5 and 10 per-unit changes for all tests except basophils, monocytes, plasma viscosity, and mGPR, for which 0.1 and 0.5 per-unit changes were used, as these four tests are on the decimal scale for the units used. | *Table 3 footnote:*  “HRs are presented for a one unit change in the blood test per year and the associated hazard rate of cancer within 6 months.  Table S4 added.  *Results, Association between blood test trend and cancer diagnosis, Unadjusted analyses, paragraph two:*  “HRs for blood test trend were rescaled to represent unit changes per year that are more clinically meaningful (Table S4).”  *Results, Association between blood test trend and cancer diagnosis, Adjusted analyses, paragraph two:*  “HRs for blood test trend were rescaled to represent unit changes per year that are more clinically meaningful (Table S4).” |
| 14 | Competing risks (e.g., death within six months) are not addressed. Given the age distribution and comorbidity burden, death may preclude cancer diagnosis, potentially biasing hazard estimates. | In our dataset, approximately 4% of patients had died within the six-month follow-up period without a cancer diagnosis. Common rule of thumb approaches suggest a competing risk analysis if the proportion is greater than 10% or if it is equal to or greater than the proportion with cancer. Neither rule was satisfied in this study, so we did not consider a competing risks analysis to be warranted. | Not applicable. |
| 15 | The rationale for limiting outcome ascertainment to six months should be better justified. A shorter or longer window could materially affect discrimination performance and hazard estimates. | Six months was chosen because previous research has shown that risk of cancer diagnosis after UWL after six months returns to background levels expected at the patient’s age and sex. We have now added this information in the Outcome section and included a reference. | *Methods, Test methods, first paragraph:*  “Six months was chosen because previous research demonstrated that the risk of cancer diagnosis after six months following UWL returned to the background levels expected at the patient’s age and sex[15].” |
| 16 | Model calibration is not reported. While AUC evaluates discrimination, calibration metrics (e.g., calibration slope, calibration-in-the-large, calibration plots) are necessary to assess clinical utility in a diagnostic context. | Apologies again that it was unclear, but we did not derive and validate a risk prediction model in this work, so did not assess calibration metrics. We will however include calibration metrics and net benefit plots in future predictive models. As per our previous comment, we have made it clearer now that the objective was to assess the role of blood test trend to inform cancer diagnosis. | Not applicable. |
| 17 | The LOWESS graphical exploration is described but not integrated into model specification decisions. It would be helpful to clarify whether non-linear patterns observed informed spline selection. | Yes, we can confirm that the LOWESS graphs were used to inform the knot locations. We have made this clearer in the manuscript. | *Methods, Analysis, second paragraph:*  “Linear splines and knot locations were chosen based on visual inspection of LOWESS plots and comparison of AIC and BIC of models using fractional polynomials, restricted cubic splines, linear splines, and different knot locations.” |
| 18 | The manuscript does not specify the software and packages used for joint modelling. Given the complexity of joint models, reproducibility requires software identification and key tuning parameters. | Apologies for missing this. We have now added that R version 4.5.2 was used with RStudio and that JoineRML package was used to derive the joint models. | *Methods, Analysis, first paragraph:*  “Analyses were performed using RStudio (R version 4.5.2).”  *Methods, Analysis, second paragraph:*  “Joint modelling of longitudinal and time-to-event data was used to assess trends for cancer diagnosis using the JoineRML package.” |
| 19 | It is unclear whether laboratory measurements were standardised across time and laboratories beyond unit harmonisation. Changes in assay methodology over an 18-year period could introduce systematic measurement shifts. | The CPRD does not hold details of the laboratory that processed the blood samples. Therefore, no standardisation across laboratories was possible. We did however calculate descriptive statistics of each blood test by year, following unit standardisation, and found no patterns to suggest that further standardisation was necessary. | Not applicable. |
| 20 | The exclusion of negative laboratory values is mentioned but not justified. It should be clarified whether these represent data errors or physiologically implausible values. | Negative blood test values would be data entry errors. We have now clarified this in the manuscript. | *Methods, Test methods, clinical features:*  “Continuous results of these blood tests available over 10 years prior to UWL were obtained, with values less than zero dropped, as these were data entry errors, and…” |
| 21 | Age is modelled linearly (with splines in longitudinal sub-models), but no justification is provided for not exploring non-linear age effects in the Cox models for abnormality comparisons. | We included age in the models simply to adjust for it. Our goal was not to derive a prediction model, so we did not consider it necessary to model age in a way that best fits the data. | Not applicable. |
| 22 | The approach to deriving inflammatory ratios (NLR, PLR, mGPS) is described, but the impact of categorisation versus continuous modelling is not discussed. | The categorised scores are clinically relevant and coincide with how blood test abnormalities are used in practice. We have now made this clearer in the Methods. Discrimination of the categorised score was assessed using Cox models and the trend in the quantitative counterparts using the joint model. | *Methods, Test methods, Clinical features:*  “Each continuous blood test result was converted into a categorical result (normal/abnormal) using reference ranges used in clinical practice (Table S1). |
| 23 | The description of censoring could more clearly distinguish administrative censoring from event-based censoring. | We have now updated the Results section to report how many patients were censored before reaching the end of six-month follow-up, distinguishing between censoring due to death and administrative censoring. | *Results, Summary of patient data, second paragraph:*  “There were 6.5% (n=17,909) of patients who were censored before reaching the end of six-month follow-up (4.4%, n=12,221 deaths; 2.1%, n=5,688 de-registration with the practice, data cut date for the practice, 31/12/2018, or six months following UWL).” |
| 24 | Sub-group AUC analyses (by age, sex, stage, number of repeat tests) are mentioned, but statistical uncertainty and interaction testing procedures are not described. | These subgroup analyses are exploratory in nature and hypothesis-generating. Our goal with these were to get a sense for how well abnormality and trend work in different patient groups. We have now made this clearer in the manuscript. Apologies again, but our goal was not to derive a predictive model, so we did not consider it necessary to test for interactions between variables. To reduce emphasis on subgroup analyses, we have removed them all, except analyses by cancer site and by the number of repeat tests. | *Methods, Analysis, fifth paragraph:*  “As exploratory analyses, we looked at the AUC of abnormality and trend by cancer site and by the number of repeat tests used to derive the trend.” |
| 25 | There is no reporting of effective sample size within each trend window after applying the ≥2-test restriction. This is particularly important for shorter windows (e.g., 1-year trend). | We have now performed a sample size calculation for a sample of five blood tests (albumin, AST, haemoglobin, CRP, and plasma viscosity) in the 2+ test cohort in the 1-year trend window (as this is the smallest cohort). We have also explored varying levels of alpha and beta/power in our calculation, as our dataset is large. Our sample sizes exceed the calculated limits. We have included this in a new paragraph at the end of the Analysis section in the Methods. | *Methods, Analysis, Sample size:*  “We performed a sample size calculation for an AUC for five blood tests (albumin, AST, haemoglobin, CRP, and plasma viscosity – AST and plasma viscosity cohorts are the smallest cohorts) in the 1-year trend window and 2+ blood test cohort (Table S2). For each test, the null AUC was 0.5 and target AUC was 0.7. A two-sided 0.048% alpha, accounting for multiple testing (a Bonferroni adjustment to a 5% significance level considering 104 test-trend window combinations), and 80% power was used. The calculation also uses the ratio of the number of cancer-free patients to the number of cancer patients, which we based on our observed data. Based on these inputs, 578-625 patients, depending on the test, were required. We repeated the calculation using 90% and 99% power due to our large sample size, with the latter increasing the sample size to 1,055-1,125.” |
| 26 | The manuscript would benefit from a flow diagram specific to the trend-analysis cohorts for each time window to clarify denominators. | The number of patients/denominators included in each trend window for each test is in the right-hand panel of Table S3. We have made this clearer in the manuscript. Thanks again for your comments. | *Results, Summary of patient data, first paragraph:*  “Among those included in the trend analysis (those with 2+ tests) (sample sizes are in Table S3)…” |
|  |  |  |  |
|  | **Reviewer 2** |  |  |
| 1 | Line 35: spell out interquartile range (ICR). | Thanks for your comments. Apologies for missing this. We have now spelt out IQR in full | *Abstract, Methods and results:*  “…the median (interquartile range (IQR)) number of blood tests…” |
| 2 | Line 85: Would the authors be more specific about how UWL was measured? First patient report of UWL? Or indication in the chart of WL between the last and most recent measure? In the case of the latter, does the duration influence the date of measurement, or is it taken from the previous measure, or the most recent measure indicating UWL? In either case what is the magnitude of loss to qualify as UWL that is taken seriously? Not need to answer these questions specifically, but it would be good to operationalize the measure of UWL for those of us who are perhaps aware that there is a clear, well understood definition. | We included UWL that was recorded in the GP software as SNOMED-CT coded UWL (previously Read codes) – the decision to code is at the discretion of the GP. If a patient had multiple UWL codes, the first was selected as the index date. We have also described in the Strengths and limitations section that we used UWL codes that underwent prior validation, where UWL code usage was cross-referenced with change in quantitative weight values over time, and have included a reference to that validation study. | *Discussion, Strengths and limitations, paragraph 4:*  “However, we build on previous analyses using a validated list of UWL Read codes^[21]^, where UWL code usage was cross-referenced with change in quantitative weight values over time[15],…” |
| 3 | Line 167: spell out RBC and CRP. | We have now spelled out RBC and CRP. | *Results, Summary of patient data, first paragraph:*  “The most common blood test abnormalities on the most recent blood test were low red blood cell count (RBC)… and high C-reactive protein (CRP)…” |
| 4 | Line 180: spell out ESR. | We have now spelled out ESR. | *Results, Summary of patient data, first paragraph:*  “…and inflammatory markers CRP, erythrocyte sedimentation rate (ESR), and plasma viscosity” |
| 5 | Line 200:  spell out ALT.....the authors could spell out some of these abbreviations, say in Table 2 if they are blood tests, but I won't mention this again since I suspect the list will grow longer. However, the reader who are not clinicians need to know what these abbreviations represent. | We have now spelled out ALT. We have now gone through the manuscript and added the full spelling for abbreviations, where missed. We have | *Results, Association between blood test trend and cancer diagnosis, first paragraph:*  “…with no association for alanine transaminase (ALT)”  Further abbreviations spelled out at first use throughout the manuscript.  Abbreviations added as footnotes to Tables 1, 2, and 3 and Figures 1, 2, and 3. |
| 6 | Line 310: The authors assert that the unstructured nature of primary care records can be seen as a strength....is it the records, or the variability of encounters and the test that are chosen during those encounters? I would presume that whatever is done is somewhat recorded, especially laboratory tests. The statement implies that the unstructured nature of records is a strength over a highly structured record. Is this what is meant?  I see the point made beginning on line 313, so maybe rather than a strength, it is not a data liability? | Apologies that this was unclear. We meant that our data is a snapshot of clinical practices, where encounters and blood testing vary between patients. We agree that a structured dataset would be ideal, but we meant that it is a strength that our modelling strategy offers a pragmatic approach to use data available as-is to support cancer detection in practice. We have now re-worded this in the Discussion to make this clearer. | *Discussion, Strengths and limitations, second paragraph:*  “A further strength is that we utilised dynamic modelling to assess the association between blood test trend and cancer diagnosis, which can accommodate the unstructured nature of primary care records.” |
| 7 | In the discussion, where implications for research and clinical practice are discussed, the authors report that their findings illustrate that blood test abnormalities and trends have greater discrimination for cancer diagnosis overall and for specific cancers after adjustment for age and sex. If I'm a GP, and considering the implications of these data, I believe my first thought would be that these findings are potentially very useful, but  "a lot of moving parts that would be hopeless for me to manage on my own. The authors then say it would not straightforward to provide the adjusted rates by the laboratory, or if not, the  EHR could do the math, providing the GP, and perhaps go further to provide the GP with an alert about the possible indication for cancer. The authors then caution that "Although these changes  would require relatively simple modifications to existing processes and electronic health record functionality, implementation evaluations should consider their impact on clinical workflows, risk communication, clinician behaviour, cost effectiveness, and clinical outcomes."  Then, in closing, they say "Blood test abnormalities should be interpreted after age and sex adjustment to improve triage for cancer investigation in patients attending primary care with UWL. Evaluating historical trend leading up to the UWL offers further discrimination after adjustment for some test-cancer combinations."   It seems to me that this study has found something very important (A) and of immediate practical use (B), but it is not clear how to get from A to B, and they caution, we should not until consideration has been given to clinical workflows, risk communication, clinician behaviour, cost effectiveness, and clinical outcomes.  I would encourage the authors to be bolder and stress that these observations highlight the potential for a new tool to reduce delay in diagnosis, but have too many elements for GPs to process on their own. Thus, the need for EHR algorithms and perhaps an AI tool, which should be a very high immediate priority in order to proceed to the next step to determine the feasibility of their use in community health settings.   It still may not be clear whose responsibility it would be to take up the charge, but at least you would have made it clear that some health authority should embrace that responsibility. | We agree that we should emphasise the transition between our findings and practical implications more. Our plan is to derive and validate a risk prediction model that incorporates, age, sex, blood test abnormalities and trends, and potentially further covariates. Such a prediction model is intended to be implemented into the primary care electronic system to make cancer risk predictions and support GP decision making. We have now updated the Discussion section to make this clearer and have also added a sentence in the Conclusion. Thanks again for your comments. | *Discussion, Implications for research and clinical practice, second paragraph:*  “Rather, our findings highlight some test-cancer pairings for which trend has the potential to enhance the selection of patients with UWL for cancer investigation in primary care. Dynamic prediction models that utilise age, sex, and the combinations of blood test abnormalities and blood test trends identified in this manuscript could be combined with additional covariates, such as patient comorbidity. Such an approach could be implemented into primary care electronic systems after validation to support GP decision making.”  *Discussion, Conclusion:*  “Simple decision rules or a risk prediction model incorporating this data could support patient selection in primary care.” |
|  |  |  |  |
|  | **Reviewer 3** |  |  |
| 1 | Most of the results presented require the reader to check supplementary data, as often the summary in text is quite brief. I was wondering if narrowing down the focus of this paper  would help? There are results on median N of tests per patient, median N of tests per cohort, 1,3,5 and 10-year trends (increasing or decreasing, for specific blood tests), discriminative ability for all cancers, and for specific cancers, diagnostic performance for abnormalities and trends, with and without adjustment for sex and age. I am quite happy to get a rebuttal about this, but the amount of information at times made it challenging to understand the results. If narrowing down the focus is not possible, I think a good compromise would be to highlight in the supplementary figures/tables some of the key information described in the manuscript. For example, if trends were favoured for some tumour types, these "favoured trends" could be shown in bold, or highlighted in a different colour. This would help the reader navigate the supplementary data. Often, AUCs are very similar across all comparisons, and being able to clearly see the results being emphasised in the manuscript would be very helpful. | Thanks for your comments. We agree that the supplementary file is quite long. We have now updated the supplementary by removing many tables and figures, primarily large tables of subgroup analyses, which are now instead available on GitHub. | Supplementary file has shortened.  Tables/figures that have been removed are now available on GitHub, which is referenced.  Many in-text citations to supplementary files have been updated to reflect these changes. |
| 2 | In the results section, line 177, it is stated that "graphs of blood test trends over the 10-year study period are in the figures S2-S27". The evidence from these figures is then summarised in three sentences. It was only after looking at the supplementary data (and I thought that these figures were fantastic and very informative) that everything made more sense. I understand that these figures need to be supplementary, but I would recommend adding a bit more information to the paragraph (lines 177-182), perhaps adding some values, and explaining what was meant by limited data. | We have now moved Table 2 to the supplementary file (as per your later comment) and in its place, we have created a new plot that shows examples of LOWESS graph trends, so that readers would not have to rely solely on the supplementary file to understand this section.  We have also removed the bit about “limited data” when commenting on the trends in haematocrit and RDW. | Table 2 (blood test summary) moved out of the main manuscript and replaced by a new Figure 2 (examples of trend plots).  *Results, Summary of patient data, third paragraph:*  “Haematocrit and red cell distribution width (RDW) trends also exceeded the normal range limit in younger adults aged 50 years or below” |
| 3 | Lines 368-375: I think these sentences synthesise quite well the key findings re. investigated trends, but they are a bit lost in the long paragraph. I did wonder if these should be the first paragraph in this subsection (implications) as this information is more closely linked with the study aim compared to the information provided in the previous paragraph. | We agree that these sentences reflect key findings. We have therefore moved them to be the first and a standalone paragraph in the implications section. | *Discussion, Implications for research and clinical practice, first paragraph:*  “We identified scenarios in which blood test trends may warrant further evaluation to focus or augment the diagnostic work-up in patients presenting with UWL. Changes over a short 1-year period were most discriminative for specific cancers. Whilst these blood tests are commonly used in practice, they are not commonly used to test for the cancers we report an association for trend. Many of these cancers often have an existing, more specific, primary care test. Further research should therefore investigate associations between MCV trend and colorectal cancer in combination or as triage to Faecal Immunochemical Test (FIT) testing, AST, RBC, haematocrit, and PLR trend in relation to Prostate Specific Antigen (PSA) testing for prostate cancer, and WBC trend as triage to thoracic imaging for lung cancer.” |
| 4 | Lines 375-385: I would consider rewriting some of this. It may be a bit so soon to suggest that adjusted trends over 3 or more years may improve triage to specialist services, or future triage for multi-cancer testing. I would perhaps remove this bit and just acknowledge that any improvements seen for adjusted trends were modest (as stated later in the paragraph) and it would not be appropriate to recommend implementation (what is meant by socio-technical?) before their utility can be better ascertained, whether for all cancers, or for specific tests and tumour types. In lines 384-385, it may be worth reiterating that this possibility needs to be further investigated | We agree and have now amended some of the text in this section. We hope this is now worded more appropriately. | *Discussion, Implications for research and clinical practice, third paragraph:*  “Adjusted trends over three or more years in 12 blood test components showed superior discrimination to adjusted abnormality. However, the improvements were modest and there are significant implementation considerations to analysing blood test trends within the primary care electronic health record that need to be studied to permit real-time trend analysis. Our results will also need external validation. It may be that the more immediate and substantial gains made by focusing on age and sex adjustment of blood test abnormalities should be prioritised over the smaller additional gains made by adding trend analysis. This may not be the case for symptomatic presentations with less metabolic derangement, or for analysing blood test trend without accounting for symptoms, but this needs further investigation.” |
| 5 | Lines 388-390: The conclusion could have been a bit punchier. The last sentence could be expanded a bit (389-390): improvements related to trends are often seen  one year prior to UWL for some cancers, but the utility of incorporating trends to improve triage for patients with UWL need to be further investigated. | We agree with your suggestion and have now updated the Conclusion. | *Discussion, Conclusion:*  “Blood test abnormalities should be interpreted after age and sex adjustment to optimise patient selection for cancer investigation in patients attending primary care with UWL. Evaluating historical trend within one year prior to UWL offers further discrimination, after adjustment by age and sex, for some test-cancer combinations, but the utility of incorporating trend to improve patient triage requires further investigation. Simple decision rules or a risk prediction model incorporating this data could support patient selection in primary care.” |
| 6 | Authors and affiliations: I have noticed a typo you may wish to correct. Should it be Clare Bankhead instead of Clare Bankead? | Apologies for the error. This has now been corrected. | *Author list:*  “…Clare Bankhead…” |
| 7 | Abstract, methods: Apologies if I missed this in the main manuscript, were the 26 blood tests decided a priori or do they refer to all different blood tests identified in CPRD records? If the former, what were the criteria for choosing the tests? | The 26 tests were decided a-priori. We aimed to study commonly available tests (liver function test and full blood count), to maximise uptake and impact of abnormality and trend, and tests that are triggered by UWL presentation (inflammatory markers). We have now made this clearer in the Methods section | *Methods, Test methods, Clinical features:*  “These tests were decided a-priori, as our goal was to study commonly available tests, to maximise uptake and impact of abnormality and trend, and tests that are triggered by UWL presentation” |
| 8 | Introduction, lines 55-56. For the interest of the audience outside the UK, please specify that NICE guidelines are used in England and Wales | We have now updated the Introduction, as requested. | *Introduction, first paragraph:*  “Low albumin, raised platelets, raised calcium, and raised inflammatory markers increase the risk of cancer overall above the 3% threshold recommended by the National Institute for Health and Care Excellence (NICE) for urgent investigation for cancer in England and Wales” |
| 9 | Methods, lines 137-139: Please provide the rationale for choosing these timeframes. Were these based on clinical knowledge, previous studies, natural history of specific cancers, etc.? | Studies commonly use a look back period of 1 year to identify predictors, but we wanted to explore how different levels of trend influences diagnostic performance. We inspected our LOWESS plots, which showed that trends in cancer cases often begin to diverge from cancer-free patients around 3-5 years before the diagnosis. We therefore opted for 1-, 3-, and 5-year trend windows prior to UWL. We expected that earlier blood tests were unlikely to be helpful, but felt we needed to assess this to be sure. Hence, we also included a 10-year trend window. | *Methods, Analysis, third paragraph:*  “Our LOWESS plots suggest that cancer-related trends begin to diverge from cancer-free patients around 3-5 years before diagnosis, so we opted to investigate 1-, 3-, and 5-year trend windows prior to UWL. We expected that earlier blood tests were unlikely to be helpful and chose to assess this also by including a 10-year trend window.” |
| 10 | Methods, lines 147-148: Table S1 is very useful, but please also consider clarifying which organisation/country/health system established the norms/reference ranges used for the blood tests. | We have now added that the ranges were taken from the Oxford Handbook of Clinical Medicine. We have also added a footnote to Table S1 to explain this. | *Methods, Analysis, fourth paragraph:*  “Blood test abnormality on the co-occurring test was defined using reference ranges reported in the Oxford Handbook of Clinical Medicine (Table S1)”  *Table S1 footnote:*  “Blood test references ranges used to define abnormality were taken from: Oxford Academic. Oxford Handbook of Clinical Medicine - Reference intervals, etc. 2017 [Accessed 21 January 2026]” |
| 11 | Table 1: Were the ethnicity categories the ones provided by CPRD? Consider adding a footnote about what is included in each category. Each of these may include heterogenous groups, with varying cancer risk (I understand that larger groups are needed to carry out the analyses). | The ethnicity codes were primarily extracted from HES, with missing ethnicity data taken from CPRD, if available. Ethnicity data is more complete in HES than in CPRD, but HES does not provide very granular information. We extracted a minority of missing ethnicities from CPRD using SNOMED-CT codes (which are available on our GitHub with the other code lists), which are more granular than in HES, but had to group these to match what HES provides, i.e. the groupings in Table 1. | Not applicable. |
| 12 | Table 1: Please clarify if IMD1 is most and IMD 5 is least deprived, just to avoid any confusion from the reader. This also applies to some supplementary tables. | IMD1 is the least deprived and IMD5 is the most. We have now made this clearer in Table 1 and in the supplementary tables. | *Table 1:*  “1 (least deprived)”  “5 (most deprived)” |
| 13 | Table 2.: While this is relevant, I wonder if just providing some information in text would be sufficient? There are many excellent supplementary tables that could be in the manuscript instead. | We agree and have now moved Table 2 to the additional file to be uploaded onto GitHub. In its place, we have created a new figure that gives examples of LOWESS plot trends | Table 2 removed from the main manuscript.  Figure 2 added to illustrate examples of trends. |
| 14 | Page 20, line 298: "Strengths and limitations" instead of "Strengths and limitation"? | Apologies for the error. We have now corrected this. | *Discussion:*  “Strengths and limitations” |
| 15 | Discussion, lines 304-307: The statement "Weaker associations were found in small subgroups with smaller sample sizes" seems neutral, but since it follows sentence about stronger associations with larger sample sizes being identified for larger cancer groups, it implies that if sample sizes were larger for other cancer types, stronger associations were likely to be found. Perhaps consider a more neutral approach - if samples were larger, one would be able to test whether there were also stronger associations for other cancers. | Apologies this was unclear. We meant that larger samples would provide ‘more precise’ associations, not ‘stronger’ associations. We have now corrected the text. | *Discussion, Strengths and limitations, first paragraph:*  “Future work investigating the clinical utility of trend in more common symptoms than UWL may lead to more precise associations with trend, overall and by subgroup.” |
| 16 | Table S4 - Can the authors please clarify what is meant by symptom co-occurring? | Table S4 has been moved from the supplementary to the file available on GitHub. In that file, we have now added a footnote to explain that co-occurring symptoms are those that were recorded in the three months prior to one month after UWL. | *Tables 2-5 in the GitHub file (previously Tables S4-S7 in the supplementary file):*  ““Symptom co-occurring” – co-occurring symptoms were those recorded in the three months before to one month after the UWL date.” |
| 17 | Discussion: It may be worth adding somewhere that perhaps results could differ if trends were adjusted for other (or additional) patient characteristics such as lifestyle behaviours or ethnicity - bearing in mind the limitations regarding the amount of missing data for these variables. | We have now added some text on this in the Discussion. | *Discussion, Implications for research and clinical practice, fourth paragraph:*  “Future work could adjust the trend for additional variables beyond age and sex. For example, ethnicity, deprivation score, and smoking status. We did not adjust for these additional variables, as we prioritised complete and accurate data electronic health records data (age, sex, and blood tests) and our analysis is exploratory.” |
| 18 | In table S9 CIs are missing for some tests - a footnote explaining this would be useful. Is it because numbers are too small? | Yes, that is correct. Some blood test and cancer site combinations gave too small numbers for a SE and CI to be derived. We have now added a footnote in Table S9 to clarify. | *Table S6 (previously Table S9):*  “For some cancer sites, CIs are missing because there were insufficient events to estimate a standard error and therefore the CI.” |
| 19 | In Table S12 the AUCs and CIs are very similar for CRP and ESR. This may just be a coincidence, but since they are so close to each other in the table could the authors please check in case there is some repeated information there? | We have checked this table (now Table S7) and confirm that these AUCs and CIs for CRP and ESR are the often the same to 2 decimal places. From 3 decimal points and beyond, they differ, so they appear the same due to rounding. | Not applicable. |
| 20 | Overall: what made the authors choose to report on AUCs other than other measures of diagnostic performance? | We chose the AUC because this is a high-level summary metric. If our study was a prospective diagnostic test accuracy study, we would have also considered further metrics, such as predictive values. | *Methods, Analysis, fifth paragraph:*  “Performance of trend and abnormality was assessed using the fixed-time area under the curve (AUC) at six months following UWL as a high-level summary metric of discrimination across multiple comparisons.” |
