# Supplementary material for: Blood test trend versus single threshold abnormality to discriminate cancer from non-cancer in patients with unexpected weight loss: A retrospective cohort study
Source: PLoS Med. 2026 Sep 17;23(9):e1004956. doi: 10.1371/journal.pmed.1004956 (PMC13585255; doi:10.1371/journal.pmed.1004956)
Supplement: Supplementary file 5 [file pmed.1004956.s007.docx]

| **No.** | **Reviewer comment** | **Author response** | **Changes made** |
| --- | --- | --- | --- |
|  | **Editor** |  |  |
| 1 | Please state why you looked at data only to 2018, and not more recent data. Did any of the blood tests change over that period? You should comment on how these conclusions could be validated. Is the detection window of 6mo also a limitation? Maybe you should have considered within 1 yr? | Thanks for your comments. We looked at data up to 2018 because this corresponds to the date of the most recent NCRAS data downloaded for validated cancer outcomes. We have made this clearer in the Methods section.  Blood test processes have not changed since 2018 in a way that would influence our results.  We have made clearer in the Discussion section that external validation requires assessment of performance in further datasets.  Six months was chosen as the outcome window because previous research demonstrated that the risk of cancer diagnosis after six months following UWL returned to the background levels expected at the patient’s age and sex. Hence, we did not consider 1 year or longer. This detailed is described in the Methods, Outcome section, with a reference included to the prior research study. | *Methods, study design:*  “The end date of 2018 coincided with the most recent NCRAS data download for cancer outcomes.”  *Discussion, Implications for research and clinical practice, third paragraph:*  “Our results will also need external validation, by deriving performance measures in further primary care datasets.” |
| 2 | We think it would be improve clarity if you could add what the main objective of the study is to the Abstract. The Methods and Findings section of the Abstract does not (to my read) crystallize what they aimed to find out, and what you found. | We agree and have now updated the Background section in the Abstract to make the main objective clearer. | *Abstract, Background:*  “Unexpected weight loss (UWL) is a non-specific cancer symptom. Abnormalities in the most recent blood test contribute to cancer referral decisions in primary care. Interpreting historical changes (trends) in blood tests could enhance discrimination for undiagnosed cancer. We aimed to assess the discrimination of blood test trend for undiagnosed cancer and compare this to that of blood test abnormality in patients presenting to primary care with with UWL.” |
| 3 | Please confirm that your title complies with PLOS Medicine's style. Your title must be nondeclarative and not a question. It should begin with main concept if possible. "Effect of" should be used only if causality can be inferred, i.e., for an RCT. Please place the study design ("A randomized controlled trial," "A retrospective study," "A modelling study," etc.) in the subtitle (ie, after a colon). | We confirm that the title complies with the PLOS Medicine style. | *Not applicable.* |
| 4 | Please confirm that your abstract complies with our requirements, including format (three sections: Background, Methods and Findings, and Conclusions) and providing all the information relevant to this study type <https://journals.plos.org/plosmedicine/s/submission-guidelines#loc-abstract> | We confirm that the abstract complies with the PLOS Medicine requirements, including format and information provided for our study type. | *Not applicable.* |
| 5 | Please ensure that the Introduction ends with a clear description of the study question or hypothesis. | We confirm that the Introduction ends with a description of the study question. | *Not applicable.* |
| 6 | Please ensure that all abbreviations are defined at first use throughout the text (including STROBE, RECORD, and STARD). | We have now ensured that all abbreviations are defined at first use. | *Throughout the manuscript:*  Definitions of the following abbreviations have now been added at first use: STROBE, RECORD, STARD, SNOMED-CT, ICD10, LOWESS, AIC, and BIC |
| 7 | Please confirm that all numbers presented in the abstract are present and identical to numbers presented in the main manuscript text. | We confirm that the numbers presented in the abstract are present and identical to numbers presented in the main manuscript text. | *Not applicable.* |
| 8 | Please disclose any use of AI tools and technologies to the study or article content at the end of the Methods. Please specify the name(s) and version(s) of any tool(s) used and a description of their use, including how the AI-assisted or generated content was evaluated by authors. Please also include a statement specifying who retains responsibility for the final manuscript. Please see our guidelines for more information: <https://journals.plos.org/plosmedicine/s/ethical-publishing-practice#loc-artificial-intelligence-tools-and-technologies>. | We confirm that no AI tool was used at any point in this study. | *Not applicable.* |
| 9 | The funding statement should include: specific grant numbers, initials of authors who received each award, URLs to sponsors’ websites. Also, please state whether any sponsors or funders (other than the named authors) played any role in study design, data collection and analysis, the decision to publish, or preparation of the manuscript. If they had no role in the research, include this sentence: “The funders had no role in study design, data collection and analysis, decision to publish, or preparation of the manuscript.” | We have now added the URLs to the websites. All other details are as suggested, where relevant. | *Funding section:*  “BDN and PSV were funded by the NIHR Policy Research Programme (Policy Research Unit on Cancer Awareness, Screening and Early Diagnosis, reference PR-PRU-NIHR206132) (<https://www.nihr.ac.uk/funding-programmes/policy-research>) and a Cancer Research UK Clinical Careers Committee Postdoctoral Fellowship (RCCPDF\100005) (<https://www.cancerresearchuk.org/for-researchers/apply-for-and-manage-your-funding/how-we-make-funding-decisions/research-careers-committee>). FDRH and CWD were supported by the NIHR ARC OTV (<https://www.arc-oxtv.nihr.ac.uk/>). The funders had no role in study design, data collection and analysis, decision to publish, or preparation of the manuscript. The views expressed are those of the authors and not necessarily those of Cancer Research UK, the NIHR, or the Department of Health and Social Care.” |
| 10 | Please review your text for claims of novelty or primacy (e.g. 'for the first time') and remove this language. In addition, please check that any use of statistical terms (such as trend or significant) are supported by the data, and if not please remove them. | We have now removed claims of novelty or primacy. This applied only to the strengths and limitations section.  We confirm that statistical terms, such as trend or significant, are supported by the data. | *Discussion, Strengths and limitations section:*  OLD: “To our knowledge, this is the first large-scale analysis…”  NEW: “This is a large-scale analysis…” |
| 11 | Please remove the 'conclusions' subheading from the discussion. Please also remove any other subheadings from the discussion. | We confirm that we have now removed the sub-headings in the Discussion section. | *Discussion section:*  All sub-headings have now been removed. |
| 12 | Statistical reporting: Please revise throughout the manuscript, including tables and figures. - Please report statistical information as follows to improve clarity for the reader ""22% (95% CI [13,28]; p</=)"". - Please separate upper and lower bounds with commas instead of hyphens as the latter can be confused with reporting of negative values. - Please repeat statistical definitions (HR, CI etc.) for each set of parentheses." | We have now reported statistical information as requested, by using square brackets within curly brackets, separating upper and lower bounds with a comma instead of hyphen, and repeating statistical definitions within each set of parentheses. We did not however add “CI” within each bracket in Table 3, as we believe that this will make the table visually unappealing and the table title already clearly indicates that the parentheses show the CI. We hope this is acceptable. | *Throughout the manuscript:*  Curly brackets within curly brackets now changed to square brackets within curly brackets.  IQRs and CIs now use a comma to separate the upper and lower bounds  Statistical definitions (SD, IQR, HR, and CI) are now repeated within each set of parentheses (except in Table 3). |
| 13 | In the last sentence of the Abstract Methods and Findings section, please describe the main limitation(s) of the study's methodology. | We have now added in the key limitations in this section, as requested. | *Abstract, Methods and findings section:*  “A limitation of our modelling strategy is that it did not take into account the bias in who was tested and our trend analysis was also restricted to patients with 2 or more tests in each trend window.” |
| 14 | In the abstract, please include the important dependent variables that are adjusted for in the analyses. | We confirm the abstract states that age and sex were adjusted for in the analysis. | *Not applicable.* |
| 15 | PLOS Medicine requires that the de-identified data underlying the specific results in a published article be made available, without restrictions on access, in a public repository or as Supporting Information at the time of article publication, provided it is legal and ethical to do so. Please see the policy at   <http://journals.plos.org/plosmedicine/s/data-availability>  and FAQs at  <http://journals.plos.org/plosmedicine/s/data-availability#loc-faqs-for-data-policy>  " | We can confirm that we do not have the legal right to share any of the underlying data. The data is owned by a third party (CPRD). Due to privacy laws and the data user agreement between Oxford and CPRD, data access is subject to approval via CPRD’s Research Data Governance Team. We have updated the Data Availability Statement to make this clearer and offer guidance on how to obtain data access. | *Data Availability Statement section:*  “The data used in this study is not freely or publicly available. The data is owned and available from the CPRD. Due to privacy laws and the data user agreement between Oxford and CPRD, researchers are not authorised to share individual patient data. Data access is subject to approval via CPRD’s Research Data Governance Team, who, under the Health Research Authority Review Board, are required to review and approve access to the patient data before it can be shared. A data request application for protocol number 22_001798 can be made on the CPRD website: <https://www.cprd.com/> ^[28]^. The R code used in the analysis is available from GitHub^[14]^.” |
| 16 | PLOS defines the “minimal data set” to consist of the data set used to reach the conclusions drawn in the manuscript with related metadata and methods, and any additional data required to replicate the reported study findings in their entirety. Authors do not need to submit their entire data set, or the raw data collected during an investigation. Please submit the following data:  The values behind the means, standard deviations and other measures reported; The values used to build graphs; The points extracted from images for analysis. | As per our response above, we do not have the legal right to share any of the underlying data. This includes the data/values behind the means, standard deviations, and other measures reported. | *Not applicable.* |
| 17 | The Data Availability Statement (DAS) requires revision. For each data source used in your study:   a) If the data are freely or publicly available, note this and state the location of the data: within the paper, in Supporting Information files, or in a public repository (include the DOI or accession number).  b) If the data are owned by a third party but freely available upon request, please note this and state the owner of the data set and contact information for data requests (web or email address). Note that a study author cannot be the contact person for the data.  c) If the data are not freely available, please describe briefly the ethical, legal, or contractual restriction that prevents you from sharing it. Please also include an appropriate contact (web or email address) for inquiries (again, this cannot be a study author). | We confirm that the data is not freely available and is owned by a third party (CPRD). Due to privacy laws and the data user agreement between Oxford and CPRD, we researchers are not authorised to share individual patient data. Data access is subject to approval via CPRD’s Research Data Governance Team, who are required to review and approve access to the patient data before it can be shared. We have updated the Data Availability Statement. | *Data Availability Statement section:*  “The data used in this study is not freely or publicly available. The data is owned and available from the CPRD. Due to privacy laws and the data user agreement between Oxford and CPRD, researchers are not authorised to share individual patient data. Data access is subject to approval via CPRD’s Research Data Governance Team, who, under the Health Research Authority Review Board, are required to review and approve access to the patient data before it can be shared. A data request application for protocol number 22_001798 can be made on the CPRD website: <https://www.cprd.com/> ^[28]^. The R code used in the analysis is available from GitHub^[14]^.” |
| 18 | For studies in which a novel model is central to the manuscript's findings, as is the case here, authors are responsible for providing the source code needed to replicate the study's findings in a repository (such as GitHub, SourceForge or Bitbucket) or a cloud computing service (such as Code Ocean).  Please include a citable digital object identifier (DOI) or other persistent identifier of the code (such as through linkage with Zenodo). Protection of authors’ intellectual property will not be cause for exception. Please explain in the manuscript’s Data Availability Statement how readers can access the shared code. | We will provide the R software code in a GitHub repository and have pre-emptively provided a reference to the repository in the Data Availability Statement. We have not yet uploaded the code to GitHub, as we were waiting for further peer review comments on our manuscript before uploading our code. We will upload the code upon acceptance, as we will then be more confident that no further changes will be requested by reviewers. | *Not applicable.* |
| 19 | Thank you for agreeing to make your data available. At this time, please provide the link to the data repository and accession numbers required for access. | As per our response above, we do not have the legal right to share any of the underlying data. We will however share our R software code – the link to the repository is already provided in the Data Availability Statement. | *Not applicable.* |
| 20 | Please include the statement on code availability in the data availability statement. Please see guidance here:https://journals.plos.org/plosmedicine/s/code-sharing-guidance#loc-data-availability-statement-examples | We confirm the statement on code availability is present in the Data Availability Statement. | *Not applicable.* |
| 21 | Please provide the name(s) of the institutional review board(s) that provided ethical approval. | Ethical approval was provided by the Research Data Governance and Independent Scientific Advisory Committee through the CPRD on behalf of the Health Research Authority. This is described in the Ethics approval section. | *Not applicable.* |
| 22 | Please revise for use of patient-centered language. Please note that patient-centered language is constructed with the use of post-modified nouns (e.g. 'patients with psoriasis’ (or similar) instead of ‘psoriasis patients’) putting the person first in the sentence structure. | We have now updated the manuscript to use patient-centred language. | *Throughout the manuscript:* |
| 23 | Please include an acknowledgment of study participants. | We have now added an acknowledgement to patients who contributed data. | *Acknowledgements:*  “The authors would also like to thank the patients who provided data that contributed to the analysis.” |
| 24 | Please provide titles and legends for all figures and tables (including those in Supporting Information files). Please define all acronyms used in each figure or table in its corresponding legend. | We confirm that a title and legend is provided for all figures and tables, including those in the Supporting Information file. Acronyms are defined in the legend, where applicable. | *Not applicable.* |
| 25 | Please ensure that where relevant figures include 95% CIs. | We confirm that 95% CIs are included, where relevant. | *Not applicable.* |
| 26 | Please show graph axes beginning at zero. If this is not possible, please show a break in the axis. | We have now added axis breaks in all figures, where they do not already include zero, except In Figure 2. In Figure 2, it would be misleading to include zero on the y-axis, as it is biologically impossible for these test results to have a zero value, with the exception of mGPR (bottom right panel), where the y-axis already begins at zero. | *Figures 3 and 4:*  Axis breaks added, where applicable. |
| 27 | When a p value is given, please specify the statistical test used to determine it in the legend. | We confirm that we have not reported p-values in the manuscript. | *Not applicable.* |
| 28 | Where data points are discrete, please ensure that they are depicted in the figures as discrete data and not as a continuous line. | We confirm that discrete data are depicted as discrete in figures. | *Not applicable.* |
| 29 | Please provide the unadjusted comparisons as well as the adjusted comparisons in all relevant Tables | We confirm that both unadjusted and adjusted comparisons are provided. | *Not applicable.* |
| 30 | Please specify the variables controlled for in all relevant Tables | We have now added in the legend of Table 3 that adjustment was for age and sex. The remaining tables and figures already specify the variables controlled for. | *Table 3 legend:*  “^2^Adjusted HRs for trend have been adjusted for age at UWL and sex.” |
|  |  |  |  |
|  | **Reviewer 1** |  |  |
| 1 | The manuscript now reflects a robust and valuable contribution to subject, I am happy to recommend this work for publication and look forward to seeing its positive impact in the field | Thank you. | *Not applicable.* |
|  |  |  |  |
|  | **Reviewer 3** |  |  |
| 1 | Figure 1 - Please check if the date here is correct, i.e."31/12/2019". Should it be 2018 instead? | Thanks for your comment. We confirm that 2019 is correct. CPRD data was available up to December 2019, but was cut-off at December 2018 because this date corresponded to the latest NCRAS data-cut available for cancer outcomes. We have made this clearer in the footnote of Figure 1. | *Figure 1 legend:*  “Primary care patient data was available from January 2000 to December 2019, but was cut-off at December 2018, as this was the NCRAS data-cut date for cancer outcomes” |
