# Supplementary material for: Blood test trend versus single threshold abnormality to discriminate cancer from non-cancer in patients with unexpected weight loss: A retrospective cohort study
Source: PLoS Med. 2026 Sep 17;23(9):e1004956. doi: 10.1371/journal.pmed.1004956 (PMC13585255; doi:10.1371/journal.pmed.1004956)
Supplement: Peer review history

# Editor report: Decision Letter 0

**DOI:** 10.1371/journal.pmed.1004956.r001

**Authors:** Heather Van Epps

5 Feb 2026

Dear Dr Nicholson,

Thank you for submitting your manuscript entitled "Blood test trend vs single threshold abnormality to discriminate cancer from non-cancer in patients with unexpected weight loss in primary care: a diagnostic accuracy, longitudinal cohort study." for consideration by PLOS Medicine.

Your manuscript has now been evaluated by the PLOS Medicine editorial staff, and I am writing to let you know that we would like to send your submission out for external peer review.

However, before we can send your manuscript to reviewers, we need you to complete your submission by providing the metadata that is required for full assessment. To this end, please login to Editorial Manager where you will find the paper in the 'Submissions Needing Revisions' folder on your homepage. Please click 'Revise Submission' from the Action Links and complete all additional questions in the submission questionnaire.

For clinical studies, please upload a copy of your trial study protocol as a supporting information file. The study protocol should be the version submitted for approval to the institutional review board or ethics committee, should include any amendments to the study protocol, as well as the date of their approval by the institutional review or ethics committee. Please also detail any deviations from the study protocol in the Methods section of your manuscript. The editors will consider the protocol and study conduct prior to a final decision for external review.

Please re-submit your manuscript within two working days, i.e. by Feb 09 2026 11:59PM.

Login to Editorial Manager here: https://www.editorialmanager.com/pmedicine

Once your full submission is complete, your paper will undergo a series of checks in preparation for peer review. Once your manuscript has passed all checks it will be sent out for review.

Feel free to email us at plosmedicine@plos.org if you have any queries relating to your submission.

Kind regards,

Heather Van Epps, PhD

Consulting Editor

PLOS Medicine

# Peer review: Decision Letter 1

**DOI:** 10.1371/journal.pmed.1004956.r002

**Authors:** Alexandra Tosun

13 Apr 2026

Dear Dr Nicholson,

Many thanks for submitting your manuscript "Blood test trend vs single threshold abnormality to discriminate cancer from non-cancer in patients with unexpected weight loss in primary care: a diagnostic accuracy, longitudinal cohort study." (PMEDICINE-D-26-00455R1) to PLOS Medicine. The paper has been reviewed by subject experts and a statistician; their comments are included below and can also be accessed here: [LINK]

As you will see, the reviewers agree that the topic is important, but the statistical review identifies major methodological and interpretive weaknesses, while the subject reviewers note that the clinical relevance is insufficiently clear and the presentation potentially overly complex. After discussing the paper with the editorial team and an academic editor with relevant expertise, I'm pleased to invite you to revise the paper in response to the reviewers' comments. We plan to send the revised paper to some or all of the original reviewers, and we cannot provide any guarantees at this stage regarding publication.

In addition to these revisions, you may need to complete some formatting changes, which you will receive in a follow up email. A member of our team will be in touch with a set of requests shortly. If you do not receive a separate email within a few days, please assume that checks have been completed, and no additional changes are required.

When you upload your revision, please include a point-by-point response that addresses all of the reviewer and editorial points, indicating the changes made in the manuscript and either an excerpt of the revised text or the location (eg: page and line number) where each change can be found. Please also be sure to check the general editorial comments at the end of this letter and include these in your point-by-point response. When you resubmit your paper, please include a clean version of the paper as the main article file and a version with changes tracked as a marked-up manuscript. It may also be helpful to check the guidelines for revised papers at http://journals.plos.org/plosmedicine/s/revising-your-manuscript for any that apply to your paper.

We ask that you submit your revision by May 04 2026. However, if this deadline is not feasible, please contact me by email, and we can discuss a suitable alternative.

Don't hesitate to contact me directly with any questions (atosun@plos.org).

Best regards,

Alexandra

Alexandra Tosun, PhD

Senior Editor

PLOS Medicine

atosun@plos.org

-----------------------------------------------------------

Comments from the academic editor:

This is a potentially important topic, particularly as the volume of data in national health registries continues to expand and clinical models can become increasingly complex. The findings are intuitive and warrant dissemination to the scientific community, even if their practical implementation may be challenging.

-----------------------------------------------------------

Comments from the reviewers:

Reviewer #1: Major Comments

1) The primary estimand is not clearly defined. It remains ambiguous whether the objective is etiological (association between blood test trend and cancer diagnosis) or predictive/diagnostic (risk stratification and discrimination). The manuscript interchanges hazard ratios and AUC metrics without clearly specifying whether the modelling framework is intended for risk prediction or hypothesis testing.

2) The manuscript does not report any evaluation of the proportional hazards assumption (e.g., Schoenfeld residuals), which is essential when Cox modelling is used.

3) The use of Cox proportional hazards models with a fixed six-month follow-up window requires stronger justification. Given the short and bounded follow-up period, logistic regression could provide equivalent discrimination assessment without reliance on proportional hazards assumptions.

4) The handling of time origin and potential immortal time bias requires clarification. Patients were required to have at least one blood test within 10 years prior to UWL and, for trend analyses, at least two tests including one near UWL. This selection may introduce conditioning on future information relative to earlier time points. The temporal structure should be more clearly articulated to ensure no forward-looking bias is introduced into the risk models.

5) The joint modelling specification requires more detail and justification. Although mixed-effects models with random intercept and slope are described, the functional form of the association between the longitudinal process and the survival outcome is not explicitly stated (e.g., current value parameterisation, slope parameterisation, shared random effects). The exact structure linking the longitudinal sub-model to the Cox model should be clarified.

6) The use of piecewise linear splines for time-to-UWL and age-at-UWL requires methodological justification. The rationale for knot placement at 6 months and 1, 2, and 3 years is not provided. It is unclear whether alternative knot configurations were explored or whether model fit diagnostics were conducted.

7) The decision to model each blood test separately limits interpretability and may inflate Type I error due to extensive multiplicity. Twenty-six blood tests across multiple time windows and cancer subtypes generate a large number of comparisons. No correction for multiple testing or false discovery control is reported.

8) Discrimination assessment using AUC derived from Cox models requires clarification. It is not specified whether time-dependent ROC curves were used or whether a fixed-time AUC at six months was estimated. Standard AUC methods are not directly applicable to censored survival data without appropriate modification.

9) No internal validation strategy is described. Given the extensive modelling and comparison of trends versus abnormalities, overfitting is a concern. There is no mention of bootstrapping, cross-validation, shrinkage, or optimism correction.

10) Missing data handling is insufficiently described. Several baseline covariates (e.g., ethnicity, BMI, smoking) show substantial missingness. It is unclear whether these were excluded from modelling, included as missing categories, or addressed using multiple imputation. Even if not included in final models, their exclusion requires justification.

11) The requirement of at least two blood tests within a given window introduces selection bias toward patients with greater healthcare utilisation. This may induce collider bias if healthcare contact is associated with both blood testing frequency and cancer risk. The implications of restricting to 2+ tests for trend modelling should be discussed and sensitivity analyses considered.

12) The comparison between "trend" and "abnormality" may not be methodologically equivalent. The abnormality model uses a binary threshold, whereas the trend model uses continuous longitudinal modelling with random effects and spline functions. This structural difference may inherently favour trend-based modelling due to greater modelling flexibility. A fairer comparison would require aligning model complexity.

13) Effect sizes for trends (e.g., HRs close to 1.00-1.08) suggest very small per-unit effects. Interpretation of the unit of change (per SD? per raw unit? per year?) is not sufficiently clear. Clinical interpretability of these HRs requires more explicit scaling.

14) Competing risks (e.g., death within six months) are not addressed. Given the age distribution and comorbidity burden, death may preclude cancer diagnosis, potentially biasing hazard estimates.

15) The rationale for limiting outcome ascertainment to six months should be better justified. A shorter or longer window could materially affect discrimination performance and hazard estimates.

16) Model calibration is not reported. While AUC evaluates discrimination, calibration metrics (e.g., calibration slope, calibration-in-the-large, calibration plots) are necessary to assess clinical utility in a diagnostic context.

Minor Comments

17) The LOWESS graphical exploration is described but not integrated into model specification decisions. It would be helpful to clarify whether non-linear patterns observed informed spline selection.

18) The manuscript does not specify the software and packages used for joint modelling. Given the complexity of joint models, reproducibility requires software identification and key tuning parameters.

19) It is unclear whether laboratory measurements were standardised across time and laboratories beyond unit harmonisation. Changes in assay methodology over an 18-year period could introduce systematic measurement shifts.

20) The exclusion of negative laboratory values is mentioned but not justified. It should be clarified whether these represent data errors or physiologically implausible values.

21) Age is modelled linearly (with splines in longitudinal sub-models), but no justification is provided for not exploring non-linear age effects in the Cox models for abnormality comparisons.

22) The approach to deriving inflammatory ratios (NLR, PLR, mGPS) is described, but the impact of categorisation versus continuous modelling is not discussed.

23) The description of censoring could more clearly distinguish administrative censoring from event-based censoring.

24) Sub-group AUC analyses (by age, sex, stage, number of repeat tests) are mentioned, but statistical uncertainty and interaction testing procedures are not described.

25) There is no reporting of effective sample size within each trend window after applying the ≥2-test restriction. This is particularly important for shorter windows (e.g., 1-year trend).

26) The manuscript would benefit from a flow diagram specific to the trend-analysis cohorts for each time window to clarify denominators.

Reviewer #2: PMEDICINE-D-26-00455R1 Blood test trend vs single threshold abnormality to discriminate cancer from non-cancer in patients with unexpected weight loss in primary care: a diagnostic accuracy, longitudinal cohort study.

Line 35: spell out interquartile range (ICR)

Line 85: Would the authors be more specific about how UWL was measured? First patient report of UWL? Or indication in the chart of WL between the last and most recent measure? In the case of the latter, does the duration influence the date of measurement, or is it taken from the previous measure, or the most recent measure indicating UWL? In either case what is the magnitude of loss to qualify as UWL that is taken seriously? Not need to answer these questions specifically, but it would be good to operationalize the measure of UWL for those of us who are perhaps aware that there is a clear, well understood definition.

Line 167: spell out RBC and CRP.

Line 180: spell out ESR

Line 200: spell out ALT.....the authors could spell out some of these abbreviations, say in Table 2 if they are blood tests, but I won't mention this again since I suspect the list will grow longer. However, the reader who are not clinicians need to know what these abbreviations represent.

Line 310: The authors assert that the unstructured nature of primary care records can be seen as a strength....is it the records, or the variability of encounters and the test that are chosen during those encounters? I would presume that whatever is done is somewhat recorded, especially laboratory tests. The statement implies that the unstructured nature of records is a strength over a highly structured record. Is this what is meant? I see the point made beginning on line 313, so maybe rather than a strength, it is not a data liability?

In the discussion, where implications for research and clinical practice are discussed, the authors report that their findings illustrate that blood test abnormalities and trends have greater discrimination for cancer diagnosis overall and for specific cancers after adjustment for age and sex. If I'm a GP, and considering the implications of these data, I believe my first thought would be that these findings are potentially very useful, but "a lot of moving parts that would be hopeless for me to manage on my own. The authors then say it would not straightforward to provide the adjusted rates by the laboratory, or if not, the EHR could do the math, providing the GP, and perhaps go further to provide the GP with an alert about the possible indication for cancer. The authors then caution that "Although these changes would require relatively simple modifications to existing processes and electronic health record functionality, implementation evaluations should consider their impact on clinical workflows, risk communication, clinician behaviour, cost effectiveness, and clinical outcomes." Then, in closing, they say "Blood test abnormalities should be interpreted after age and sex adjustment to improve triage for cancer investigation in patients attending primary care with UWL. Evaluating historical trend leading up to the UWL offers further discrimination after adjustment for some test-cancer combinations."

It seems to me that this study has found something very important (A) and of immediate practical use (B), but it is not clear how to get from A to B, and they caution, we should not until consideration has been given to clinical workflows, risk communication, clinician behaviour, cost effectiveness, and clinical outcomes.

I would encourage the authors to be bolder and stress that these observations highlight the potential for a new tool to reduce delay in diagnosis, but have too many elements for GPs to process on their own. Thus, the need for EHR algorithms and perhaps an AI tool, which should be a very high immediate priority in order to proceed to the next step to determine the feasibility of their use in community health settings. It still may not be clear whose responsibility it would be to take up the charge, but at least you would have made it clear that some health authority should embrace that responsibility.

Reviewer #3: This is a well-written paper with clear abstract and introduction, clear methods and mostly clear results section, and a substantial amount of supplementary data (248 pages). Authors investigated whether historical changes in blood tests (trends) enhanced cancer risk discrimination (abstract)/stratification (methods) in patients presenting in primary care with unexpected weight loss (UWL) - compared to abnormalities in the most recent blood test. Unadjusted and adjusted (age and sex) AUCs were calculated. Strengths include confirming cases using linked national cancer registration data, and the longitudinal cohort design using robust primary care data from the Clinical Practice Research Datalink (CPRD). It is very clear that a great amount of time was spent to prepare the submitted files, and care was taken to ensure consistent formatting across all tables and figures. I admire the level of transparency as authors shared as much as they could within the constraints of the used CPRD data. However, I do wonder if the amount of reported information was at times detrimental to understanding the key results and the key messages from the manuscript. I would recommend some revisions to the main text (and potentially supplementary data), as described below.

Major Issues

1.Most of the results presented require the reader to check supplementary data, as often the summary in text is quite brief. I was wondering if narrowing down the focus of this paper would help? There are results on median N of tests per patient, median N of tests per cohort, 1,3,5 and 10-year trends (increasing or decreasing, for specific blood tests), discriminative ability for all cancers, and for specific cancers, diagnostic performance for abnormalities and trends, with and without adjustment for sex and age. I am quite happy to get a rebuttal about this, but the amount of information at times made it challenging to understand the results. If narrowing down the focus is not possible, I think a good compromise would be to highlight in the supplementary figures/tables some of the key information described in the manuscript. For example, if trends were favoured for some tumour types, these "favoured trends" could be shown in bold, or highlighted in a different colour. This would help the reader navigate the supplementary data. Often, AUCs are very similar across all comparisons, and being able to clearly see the results being emphasised in the manuscript would be very helpful.

2.In the results section, line 177, it is stated that "graphs of blood test trends over the 10-year study period are in the figures S2-S27". The evidence from these figures is then summarised in three sentences. It was only after looking at the supplementary data (and I thought that these figures were fantastic and very informative) that everything made more sense. I understand that these figures need to be supplementary, but I would recommend adding a bit more information to the paragraph (lines 177-182), perhaps adding some values, and explaining what was meant by limited data.

3.Lines 368-375: I think these sentences synthesise quite well the key findings re. investigated trends, but they are a bit lost in the long paragraph. I did wonder if these should be the first paragraph in this subsection (implications) as this information is more closely linked with the study aim compared to the information provided in the previous paragraph.

4.Lines 375-385: I would consider rewriting some of this. It may be a bit so soon to suggest that adjusted trends over 3 or more years may improve triage to specialist services, or future triage for multi-cancer testing. I would perhaps remove this bit and just acknowledge that any improvements seen for adjusted trends were modest (as stated later in the paragraph) and it would not be appropriate to recommend implementation (what is meant by socio-technical?) before their utility can be better ascertained, whether for all cancers, or for specific tests and tumour types. In lines 384-385, it may be worth reiterating that this possibility needs to be further investigated.

5.Lines 388-390: The conclusion could have been a bit punchier. The last sentence could be expanded a bit (389-390): improvements related to trends are often seen one year prior to UWL for some cancers, but the utility of incorporating trends to improve triage for patients with UWL need to be further investigated.

Minor issues

6.Authors and affiliations: I have noticed a typo you may wish to correct. Should it be Clare Bankhead instead of Clare Bankead?

7.Abstract, methods: Apologies if I missed this in the main manuscript, were the 26 blood tests decided a priori or do they refer to all different blood tests identified in CPRD records? If the former, what were the criteria for choosing the tests?

8.Introduction, lines 55-56. For the interest of the audience outside the UK, please specify that NICE guidelines are used in England and Wales

9.Methods, lines 137-139: Please provide the rationale for choosing these timeframes. Were these based on clinical knowledge, previous studies, natural history of specific cancers, etc.?

10.Methods, lines 147-148: Table S1 is very useful, but please also consider clarifying which organisation/country/health system established the norms/reference ranges used for the blood tests.

11.Table 1: Were the ethnicity categories the ones provided by CPRD? Consider adding a footnote about what is included in each category. Each of these may include heterogenous groups, with varying cancer risk (I understand that larger groups are needed to carry out the analyses).

12.Table 1: Please clarify if IMD1 is most and IMD 5 is least deprived, just to avoid any confusion from the reader. This also applies to some supplementary tables.

13.Table 2.: While this is relevant, I wonder if just providing some information in text would be sufficient? There are many excellent supplementary tables that could be in the manuscript instead.

14.Page 20, line 298: "Strengths and limitations" instead of "Strengths and limitation"?

15.Discussion, lines 304-307: The statement "Weaker associations were found in small subgroups with smaller sample sizes" seems neutral, but since it follows sentence about stronger associations with larger sample sizes being identified for larger cancer groups, it implies that if sample sizes were larger for other cancer types, stronger associations were likely to be found. Perhaps consider a more neutral approach - if samples were larger, one would be able to test whether there were also stronger associations for other cancers..

16.Table S4 - Can the authors please clarify what is meant by symptom co-occurring?

17.Discussion: It may be worth adding somewhere that perhaps results could differ if trends were adjusted for other (or additional) patient characteristics such as lifestyle behaviours or ethnicity - bearing in mind the limitations regarding the amount of missing data for these variables.

18.In table S9 CIs are missing for some tests - a footnote explaining this would be useful. Is it because numbers are too small?

19.In Table S12 the AUCs and CIs are very similar for CRP and ESR. This may just be a coincidence, but since they are so close to each other in the table could the authors please check in case there is some repeated information there?

20.Overall: what made the authors choose to report on AUCs other than other measures of diagnostic performance?

Any attachments provided with reviews can be seen via the following link: [LINK]

--------------------------------------------------------- ---

General editorial requests:

(Note: not all will apply to your paper, but please check each item carefully)

*We ask every co-author listed on the manuscript to fill in a contributing author statement, making sure to declare all competing interests. If any of the co-authors have not filled in the statement, we will remind them to do so when the paper is revised. If all statements are not completed in a timely fashion this could hold up the re-review process. If new competing interests are declared later in the revision process, this may also hold up the submission. Should there be a problem getting one of your co-authors to fill in a statement we will be in contact. Please do not add or remove authors without first discussing this with the handling editor. You can see our competing interests policy here: http://journals.plos.org/plosmedicine/s/competing-interests.

*Please upload any figures associated with your paper as individual TIF or EPS files with 300dpi resolution at resubmission; please read our figure guidelines for more information on our requirements: http://journals.plos.org/plosmedicine/s/figures. While revising your submission, we strongly recommend that you use PLOS's NAAS tool (https://ngplosjournals.pagemajik.ai/artanalysis) to test your figure files. NAAS can convert your figure files to the TIFF file type and meet basic requirements (such as print size, resolution), or provide you with a report on issues that do not meet our requirements and that NAAS cannot fix.

After uploading your figures to PLOS's NAAS tool - https://ngplosjournals.pagemajik.ai/artanalysis, NAAS will process the files provided and display the results in the "Uploaded Files" section of the page as the processing is complete.

If the uploaded figures meet our requirements (or NAAS is able to fix the files to meet our requirements), the figure will be marked as "fixed" above. If NAAS is unable to fix the files, a red "failed" label will appear above.

When NAAS has confirmed that the figure files meet our requirements, please download the file via the download option, and include these NAAS processed figure files when submitting your revised manuscript.

*Please ensure that the paper adheres to the PLOS Data Availability Policy (see http://journals.plos.org/plosmedicine/s/data-availability), which requires that all data underlying the study's findings be provided in a repository or as Supporting Information. For data residing with a third party, authors are required to provide instructions with contact information (web or email address) for obtaining the data. Please note that a study author cannot be the contact person for the data. PLOS journals do not allow statements supported by "data not shown" or "unpublished results." For such statements, authors must provide supporting data or cite public sources that include it.

*We expect all researchers with submissions to PLOS in which author-generated code underpins the findings in the manuscript to make all author-generated code available without restrictions upon publication of the work. In cases where code is central to the manuscript, we may require the code to be made available as a condition of publication. Authors are responsible for ensuring that the code is reusable and well documented. Please make any custom code available, either as part of your data deposition or as a supplementary file. Please add a sentence to your data availability statement regarding any code used in the study, e.g. "The code used in the analysis is available from Github [URL] and archived in Zenodo [DOI link]" Please review our guidelines at https://journals.plos.org/plosmedicine/s/materials-software-and-code-sharing and ensure that your code is shared in a way that follows best practice and facilitates reproducibility and reuse. Because Github depositions can be readily changed or deleted, we encourage you to make a permanent DOI'd copy (e.g. in Zenodo) and provide the URL.

FORMATTING - GENERAL

*Abstract: Please structure your abstract using the PLOS Medicine headings (Background, Methods and Findings, Conclusions). Please combine the Methods and Findings sections into one section.

*At this stage, we ask that you include a short, non-technical Author Summary of your research to make findings accessible to a wide audience that includes both scientists and non-scientists. The Author Summary should immediately follow the Abstract in your revised manuscript. This text is subject to editorial change and should be distinct from the scientific abstract. Ideally each sub-heading should contain 2-3 single sentence, concise bullet points containing the most salient points from your study. In the final bullet point of 'What Do These Findings Mean?', please include the main limitations of the study in non-technical language. Please see our author guidelines for more information: https://journals.plos.org/plosmedicine/s/revising-your-manuscript#loc-author-summary.

*Please express the main results with 95% CIs as well as p values. When reporting p values please report as p<0.001 and where higher as the exact p value p=0.002, for example. Throughout, suggest reporting statistical information as follows to improve clarity for the reader "22% (95% CI [13%,28%]; p</=)". Please be sure to define all numerical values at first use.

*Please include page numbers and line numbers in the manuscript file. Use continuous line numbers (do not restart the numbering on each page).

*Please cite the reference numbers in square brackets. Citations should precede punctuation.

FIGURES AND TABLES

*Please provide titles and legends for all figures and tables (including those in Supporting Information files).

*Please define all abbreviations used in each figure/table (including those in Supporting Information files).

*Please consider avoiding the use of red and green in order to make your figure more accessible to those with color blindness.

SUPPLEMENTARY MATERIAL

*Please note that supplementary material will be posted as supplied by the authors. Therefore, please amend it according to the relevant comments outlined here.

*Please cite your Supporting Information as outlined here: https://journals.plos.org/plosmedicine/s/supporting-information

REFERENCES

*PLOS uses the numbered citation (citation-sequence) method and first six authors, et al.

*Please ensure that journal name abbreviations match those found in the National Center for Biotechnology Information (NCBI) databases (http://www.ncbi.nlm.nih.gov/nlmcatalog/journals), and are appropriately formatted and capitalised.

*Where website addresses are cited, please include the complete URL and specify the date of access (e.g. [accessed: 12/06/2023]).

*Please also see https://journals.plos.org/plosmedicine/s/submission-guidelines#loc-references for further details on reference formatting.

STUDY TYPE-SPECIFIC REQUESTS

(Note: not all will apply to your paper, but please check each item carefully)

*Abstract: Please include the study design, population and setting, number of participants, years during which the study took place (enrollment and follow up), length of follow up, and main outcome measures.

*Please ensure that the study is reported according to the RECORD guideline (available from https://www.record-statement.org) and include the completed checklist as Supporting Information. Please add the following statement, or similar, to the Methods: "This study is reported as per the Reporting of Studies Conducted using Observational Routinely-Collected Data (RECORD) guideline (S1 Checklist)." When completing the checklist, please use section and paragraph numbers, rather than page numbers.

*In the manuscript text, please indicate: (1) the specific hypotheses you intended to test, (2) the analytical methods by which you planned to test them, (3) the analyses you actually performed, and (4) when reported analyses differ from those that were planned, transparent explanations for differences that affect the reliability of the study's results. If a reported analysis was performed based on an interesting but unanticipated pattern in the data, please be clear that the analysis was data driven.

*Please state in the Methods section whether the study had a prospective protocol or analysis plan. If a prospective analysis plan (from your funding proposal, IRB or other ethics committee submission, study protocol, or other planning document written before analyzing the data) was used in designing the study, please include the relevant document(s) with your revised manuscript as a Supporting Information file to be published alongside your study and cite it in the Methods section. A legend for this file should be included at the end of your manuscript. If no such document exists, please make sure that the Methods section transparently describes when analyses were planned, and when/why any data-driven changes to analyses took place. Changes in the analysis, including those made in response to peer review comments, should be identified as such in the Methods section of the paper, with rationale.

*Please ensure that the study is reported according to the STARD guideline (https://www.equator-network.org/reporting-guidelines/stard/) and include the completed STARD checklist as Supporting Information. Please add the following statement, or similar, to the Methods: "This study is reported as per the Standards for Reporting of Diagnostic Accuracy (STARD) guideline (S1 Checklist)." When completing the checklist, please use section and paragraph numbers, rather than page numbers.

*Please structure your Abstract according to STARD for Abstracts (https://www.equator-network.org/reporting-guidelines/stard-abstracts/).

*Please structure the Methods section using the following sub-headings: Study design, Participants, Test methods, Analysis.

*Please include a diagram to describe the flow of participants through the study (typically figure 1).

# Author comment: Author response to Decision Letter 2

**DOI:** 10.1371/journal.pmed.1004956.r003

5 May 2026

Attachment Submitted filename: Reviewer responses V1.0.docx

# Peer review: Decision Letter 2

**DOI:** 10.1371/journal.pmed.1004956.r004

**Authors:** Katarzyna Starowicz

3 Jun 2026

Dear Dr. Nicholson,

Thank you very much for re-submitting your manuscript "Blood test trend vs single threshold abnormality to discriminate cancer from non-cancer in patients with unexpected weight loss in primary care: a diagnostic accuracy, longitudinal cohort study." (PMEDICINE-D-26-00455R2) for review by PLOS Medicine.

I have discussed the paper with my colleagues and the academic editor and it was also seen again by two reviewers. There are remaining editorial and production issues that need to be addressed in full and are listed at the end of this email. You must provide point-by-point response to each of them. Some of them might not be applicable, in that case please respond "not applicable". Not providing this in form of rebuttal will significantly delay your manuscript.

Any accompanying reviewer attachments can be seen via the link below. Please take these into account before resubmitting your manuscript:

[LINK]

***Please note while forming your response, if your article is accepted, you may have the opportunity to make the peer review history publicly available. The record will include editor decision letters (with reviews) and your responses to reviewer comments. If eligible, we will contact you to opt in or out.***

In revising the manuscript for further consideration here, please ensure you address the specific points made by each reviewer and the editors. In your rebuttal letter you should indicate your response to the reviewers' and editors' comments and the changes you have made in the manuscript. Please submit a clean version of the paper as the main article file. A version with changes marked must also be uploaded as a marked up manuscript file.

Please also check the guidelines for revised papers at http://journals.plos.org/plosmedicine/s/revising-your-manuscript for any that apply to your paper. If you haven't already, we ask that you provide a short, non-technical Author Summary of your research to make findings accessible to a wide audience that includes both scientists and non-scientists. The Author Summary should immediately follow the Abstract in your revised manuscript. This text is subject to editorial change and should be distinct from the scientific abstract.

In addition to these revisions, you may need to complete some formatting changes, which you will receive in a follow up email. A member of our team will be in touch with a set of requests shortly. If you do not receive a separate email within a few days, please assume that checks have been completed, and no additional changes are required.

We expect to receive your revised manuscript within 1 week. Please email us (plosmedicine@plos.org) if you have any questions or concerns.

We ask every co-author listed on the manuscript to fill in a contributing author statement. If any of the co-authors have not filled in the statement, we will remind them to do so when the paper is revised. If all statements are not completed in a timely fashion this could hold up the re-review process. Should there be a problem getting one of your co-authors to fill in a statement we will be in contact. YOU MUST NOT ADD OR REMOVE AUTHORS UNLESS YOU HAVE ALERTED THE EDITOR HANDLING THE MANUSCRIPT TO THE CHANGE AND THEY SPECIFICALLY HAVE AGREED TO IT.

Please ensure that the paper adheres to the PLOS Data Availability Policy (see http://journals.plos.org/plosmedicine/s/data-availability), which requires that all data underlying the study's findings be provided in a repository or as Supporting Information. For data residing with a third party, authors are required to provide instructions with contact information for obtaining the data. PLOS journals do not allow statements supported by "data not shown" or "unpublished results." For such statements, authors must provide supporting data or cite public sources that include it.

To enhance the reproducibility of your results, we recommend that you deposit your laboratory protocols in protocols.io, where a protocol can be assigned its own identifier (DOI) such that it can be cited independently in the future. Additionally, PLOS ONE offers an option to publish peer-reviewed clinical study protocols. Read more information on sharing protocols at https://plos.org/protocols?utm_medium=editorial-email&utm_source=authorletters&utm_campaign=protocols

Please review your reference list to ensure that it is complete and correct. If you have cited papers that have been retracted, please include the rationale for doing so in the manuscript text, or remove these references and replace them with relevant current references. Any changes to the reference list should be mentioned in the rebuttal letter that accompanies your revised manuscript.

Please note, when your manuscript is accepted, an uncorrected proof of your manuscript will be published online ahead of the final version, unless you've already opted out via the online submission form. If, for any reason, you do not want an earlier version of your manuscript published online or are unsure if you have already indicated as such, please let the journal staff know immediately at plosmedicine@plos.org.

If you have any questions in the meantime, please contact me or the journal staff on plosmedicine@plos.org.

We look forward to receiving the revised manuscript by Jun 10 2026 11:59PM.

Sincerely,

Katarzyna Starowicz, PhD

Associate Editor

PLOS Medicine

kstarowicz@plos.org

------------------------------------------------------------

Comments and Requests from Editors:

Please state why you looked at data only to 2018, and not more recent data. Did any of the blood tests change over that period? You should comment on how these conclusions could be validated. Is the detection window of 6mo also a limitation? Maybe you should have considered within 1 yr?

*We think it would be improve clarity if you could add what the main objective of the study is to the Abstract. The Methods and Findings section of the Abstract does not (to my read) crystallize what they aimed to find out, and what you found.

* Please confirm that your title complies with PLOS Medicine's style. Your title must be nondeclarative and not a question. It should begin with main concept if possible. "Effect of" should be used only if causality can be inferred, i.e., for an RCT. Please place the study design ("A randomized controlled trial," "A retrospective study," "A modelling study," etc.) in the subtitle (ie, after a colon).

* Please confirm that your abstract complies with our requirements, including format (three sections: Background, Methods and Findings, and Conclusions) and providing all the information relevant to this study type https://journals.plos.org/plosmedicine/s/submission-guidelines#loc-abstract

* Please ensure that the Introduction ends with a clear description of the study question or hypothesis.

* Please ensure that all abbreviations are defined at first use throughout the text (including STROBE, RECORD, and STARD).

* Please confirm that all numbers presented in the abstract are present and identical to numbers presented in the main manuscript text.

* Please disclose any use of AI tools and technologies to the study or article content at the end of the Methods. Please specify the name(s) and version(s) of any tool(s) used and a description of their use, including how the AI-assisted or generated content was evaluated by authors. Please also include a statement specifying who retains responsibility for the final manuscript. Please see our guidelines for more information: https://journals.plos.org/plosmedicine/s/ethical-publishing-practice#loc-artificial-intelligence-tools-and-technologies.

* The funding statement should include: specific grant numbers, initials of authors who received each award, URLs to sponsors’ websites. Also, please state whether any sponsors or funders (other than the named authors) played any role in study design, data collection and analysis, the decision to publish, or preparation of the manuscript. If they had no role in the research, include this sentence: “The funders had no role in study design, data collection and analysis, decision to publish, or preparation of the manuscript.”

* Please review your text for claims of novelty or primacy (e.g. 'for the first time') and remove this language. In addition, please check that any use of statistical terms (such as trend or significant) are supported by the data, and if not please remove them.

* Please remove the 'conclusions' subheading from the discussion. Please also remove any other subheadings from the discussion.

* Statistical reporting: Please revise throughout the manuscript, including tables and figures.

- Please report statistical information as follows to improve clarity for the reader ""22% (95% CI [13,28]; p</=)"".

- Please separate upper and lower bounds with commas instead of hyphens as the latter can be confused with reporting of negative values.

- Please repeat statistical definitions (HR, CI etc.) for each set of parentheses."

* In the last sentence of the Abstract Methods and Findings section, please describe the main limitation(s) of the study's methodology.

* In the abstract, please include the important dependent variables that are adjusted for in the analyses.

* PLOS Medicine requires that the de-identified data underlying the specific results in a published article be made available, without restrictions on access, in a public repository or as Supporting Information at the time of article publication, provided it is legal and ethical to do so. Please see the policy at

http://journals.plos.org/plosmedicine/s/data-availability and FAQs at

http://journals.plos.org/plosmedicine/s/data-availability#loc-faqs-for-data-policy "

* PLOS defines the “minimal data set” to consist of the data set used to reach the conclusions drawn in the manuscript with related metadata and methods, and any additional data required to replicate the reported study findings in their entirety. Authors do not need to submit their entire data set, or the raw data collected during an investigation. Please submit the following data:

The values behind the means, standard deviations and other measures reported;

The values used to build graphs;

The points extracted from images for analysis.

* The Data Availability Statement (DAS) requires revision. For each data source used in your study:

a) If the data are freely or publicly available, note this and state the location of the data: within the paper, in Supporting Information files, or in a public repository (include the DOI or accession number).

b) If the data are owned by a third party but freely available upon request, please note this and state the owner of the data set and contact information for data requests (web or email address). Note that a study author cannot be the contact person for the data.

c) If the data are not freely available, please describe briefly the ethical, legal, or contractual restriction that prevents you from sharing it. Please also include an appropriate contact (web or email address) for inquiries (again, this cannot be a study author).

* For studies in which a novel model is central to the manuscript's findings, as is the case here, authors are responsible for providing the source code needed to replicate the study's findings in a repository (such as GitHub, SourceForge or Bitbucket) or a cloud computing service (such as Code Ocean). Please include a citable digital object identifier (DOI) or other persistent identifier of the code (such as through linkage with Zenodo). Protection of authors’ intellectual property will not be cause for exception. Please explain in the manuscript’s Data Availability Statement how readers can access the shared code.

* Thank you for agreeing to make your data available. At this time, please provide the link to the data repository and accession numbers required for access.

* Please include the statement on code availability in the data availability statement. Please see guidance here:https://journals.plos.org/plosmedicine/s/code-sharing-guidance#loc-data-availability-statement-examples

* Please provide the name(s) of the institutional review board(s) that provided ethical approval.

* Please revise for use of patient-centered language. Please note that patient-centered language is constructed with the use of post-modified nouns (e.g. 'patients with psoriasis’ (or similar) instead of ‘psoriasis patients’) putting the person first in the sentence structure.

* Please include an acknowledgment of study participants.

* Please provide titles and legends for all figures and tables (including those in Supporting Information files). Please define all acronyms used in each figure or table in its corresponding legend.

* Please ensure that where relevant figures include 95% CIs.

* Please show graph axes beginning at zero. If this is not possible, please show a break in the axis.

* When a p value is given, please specify the statistical test used to determine it in the legend.

* Where data points are discrete, please ensure that they are depicted in the figures as discrete data and not as a continuous line.

* Please provide the unadjusted comparisons as well as the adjusted comparisons in all relevant Tables

* Please specify the variables controlled for in all relevant Tables

-------------------------------------------

Comments from Reviewers:

Reviewer #1: Thank you for thoroughly addressing all my comments and providing detailed clarifications in your revised manuscript. I appreciate the effort you have put into enhancing the rigor and transparency of your analyses.

The manuscript now reflects a robust and valuable contribution to subject, I am happy to recommend this work for publication and look forward to seeing its positive impact in the field.

Reviewer #3: The authors have addressed my queries very well. I have read through the responses, and checked the manuscript again. I only have one minor comment that the authors may wish to check:

Figure 1 - Please check if the date here is correct, i.e."31/12/2019". Should it be 2018 instead?

Any attachments provided with reviews can be seen via the following link:

[LINK]

# Author comment: Author response to Decision Letter 3

**DOI:** 10.1371/journal.pmed.1004956.r005

8 Jun 2026

Attachment Submitted filename: Reviewer_responses_V1.0_auresp_3.docx

# Editor report: Decision Letter 3

**DOI:** 10.1371/journal.pmed.1004956.r006

**Authors:** Katarzyna Starowicz

15 Jun 2026

Dear Dr Nicholson,

On behalf of my colleagues and the Academic Editor, Eleanor L Watts, I am pleased to inform you that we have agreed to publish your manuscript "Blood test trend vs single threshold abnormality to discriminate cancer from non-cancer in patients with unexpected weight loss in primary care: a diagnostic accuracy, longitudinal cohort study." (PMEDICINE-D-26-00455R3) in PLOS Medicine.

Before your manuscript can be formally accepted you will need to complete some formatting changes, which you will receive in a follow up email. Please be aware that it may take several days for you to receive this email; during this time no action is required by you. Once you have received these formatting requests, please note that your manuscript will not be scheduled for publication until you have made the required changes.

In the meantime, please log into Editorial Manager at http://www.editorialmanager.com/pmedicine/, click the "Update My Information" link at the top of the page, and update your user information to ensure an efficient production process.

PRESS

We frequently collaborate with press offices. If your institution or institutions have a press office, please notify them about your upcoming paper at this point, to enable them to help maximise its impact. If the press office is planning to promote your findings, we would be grateful if they could coordinate with medicinepress@plos.org. If you have not yet opted out of the early version process, we ask that you notify us immediately of any press plans so that we may do so on your behalf.

We also ask that you take this opportunity to read our Embargo Policy regarding the discussion, promotion and media coverage of work that is yet to be published by PLOS. As your manuscript is not yet published, it is bound by the conditions of our Embargo Policy. Please be aware that this policy is in place both to ensure that any press coverage of your article is fully substantiated and to provide a direct link between such coverage and the published work. For full details of our Embargo Policy, please visit http://www.plos.org/about/media-inquiries/embargo-policy/.

To enhance the reproducibility of your results, we recommend that you deposit your laboratory protocols in protocols.io, where a protocol can be assigned its own identifier (DOI) such that it can be cited independently in the future. Additionally, PLOS ONE offers an option to publish peer-reviewed clinical study protocols. Read more information on sharing protocols at https://plos.org/protocols?utm_medium=editorial-email&utm_source=authorletters&utm_campaign=protocols

Thank you again for submitting to PLOS Medicine. We look forward to publishing your paper.

Sincerely,

Katarzyna Starowicz, PhD

Associate Editor

PLOS Medicine
